# Therapeutic and toxic blood concentrations of nearly 1,000 drugs and other xenobiotics

**DOI:** 10.1186/cc11441

**Published:** 2012-07-26

**Authors:** Martin Schulz, Stefanie Iwersen-Bergmann, Hilke Andresen, Achim Schmoldt

**Affiliations:** 1 Drug Commission of German Pharmacists (AMK), Jaegerstrasse 49/50, 10117 Berlin, Germany; 2 Department of Pharmacology, Goethe-University Frankfurt, Biocenter Niederursel, Max-von-Laue-Strasse 9, 60438 Frankfurt/Main, Germany; 3 Department of Legal Medicine, Universitätsklinikum Hamburg-Eppendorf, Butenfeld 34, 22529 Hamburg, Germany

## Abstract

**Introduction:**

In order to assess the significance of drug levels measured in intensive care medicine, clinical and forensic toxicology, as well as for therapeutic drug monitoring, it is essential that a comprehensive collection of data is readily available. Therefore, it makes sense to offer a carefully referenced compilation of therapeutic and toxic plasma concentration ranges, as well as half-lives, of a large number of drugs and other xenobiotics for quick and comprehensive information.

**Methods:**

Data have been abstracted from original papers and text books, as well as from previous compilations, and have been completed with data collected in our own forensic and clinical toxicology laboratory. The data presented in the table and corresponding annotations have been developed over the past 20 years and longer. A previous compilation has been completely revised and updated. In addition, more than 170 substances, especially drugs that have been introduced to the market since 2003 as well as illegal drugs, which became known to cause intoxications, were added. All data were carefully referenced and more than 200 new references were included. Moreover, the annotations providing details were completely revised and more than 100 annotations were added.

**Results:**

For nearly 1,000 drugs and other xenobiotics, therapeutic ("normal") and, if data were available, toxic and comatose-fatal blood-plasma concentrations and elimination half-lives were compiled in a table.

**Conclusions:**

In case of intoxications, the concentration of the ingested substances and/or metabolites in blood plasma better predicts the clinical severity of the case when compared to the assumed amount and time of ingestion. Comparing and contrasting the clinical case against the data provided, including the half-life, may support the decision for or against further intensive care. In addition, the data provided are useful for the therapeutic monitoring of pharmacotherapies, to facilitate the diagnostic assessment and monitoring of acute and chronic intoxications, and to support forensic and clinical expert opinions.

## Introduction

In 2010, more than 3.95 million closed encounters regarding unintentional and intentional exposures were logged by the American Association of Poison Control Centers' National Poison Data System and 2.38 million were related to human exposures. Although the total encounters showed a 7.7% decline from 2009, human exposures with more serious outcomes increased 4.5%. Among the top four substance classes most frequently involved in all human exposures were two drug classes: analgesics (11.5%), cosmetics/personal care products (7.7%), household cleaning substances (7.3%) and sedatives/hypnotics/antipsychotics (6.0%) [1]. And a recent review describes poisoning as the second leading cause of injury-related morbidity and mortality in the United States, with more than 2.4 million toxic exposures reported each year [2]. According to the UK's National Poisons Information Service Annual Report 2010/2011, poisoning is an extremely common cause of hospital admissions in the National Health Service, being numerically similar to admissions for myocardial infarction [3].

In the case of intoxication or poisoning, the concentration of the ingested substance and/or metabolite better predicts the clinical severity of the case and the potential outcome when compared to the assumed amount and time of ingestion. In addition, it is recommended that plasma concentrations of drugs having a narrow therapeutic range or with a highly variable response (such as in psychiatry) have to be measured. This accounts for anti-epileptics, cardiac glycosides, aminoglycosides, anti-arrhythmics, theophylline, immunosuppressants, lithium, antipsychotics and antidepressants, and antiretrovirals, as well as for an increasing number of cytostatics and antimycotics, among others. Apart from acute and chronic intoxications, it is indicated to draw blood samples for the following reasons: if doses are high and borderline, if signs of overdosage occur although the dose is within normal range (for example, genetic polymorphism), if there is no efficacy although the dose is correct or if non-adherence can be expected.

In general, plasma concentrations of drugs at steady state are retrievable from the dosage and pharmacokinetic data. However, sufficient pharmacokinetic data are often not available. Moreover, searching, retrieving, reading, analysing and interpreting the relevant toxicological and critical care literature in the case of acute intoxications in daily intensive care practice is time-consuming and may delay or even mislead optimal clinical decisions. Therefore, it makes sense to offer a carefully referenced compilation of therapeutic and toxic plasma concentration ranges, as well as half-lives, of a large number of drugs and other xenobiotics for quick and comprehensive information.

## Materials and methods

The data presented in the table and the corresponding annotations (Additional file 1) have been developed over the past 20 years and longer. A previous compilation [4] has been completely revised and updated. In addition, more than 170 substances, especially drugs that have been introduced to the market since then, as well as illegal drugs, which became known to cause intoxications, were added. All data were carefully referenced and more than 200 new references were included. Moreover, the annotations providing details were completely revised and more than 100 annotations were added (see Additional file 1).

Reviews, text books, compilations of other authors (mainly [5-31]) and, most importantly, original publications concerning individual drugs and case reports have been used to set up and keep the database updated (see Additional file 1). Experience gained over more than 25 years from working in the clinical and forensic toxicological field contributed to the data presented (see Additional file 1).

The substances were selected by clinical and toxicological aspects, by frequency of prescribing or (mis-)use and other matters in the area of internal intensive care medicine as well as in clinical and forensic toxicology.

There is an increase in determining antibiotic, antiretroviral and antimycotic concentrations using analytical and chemical methods and there are special cases which are closely monitored, although therapeutic concentrations depend on the susceptibility of the microorganisms and tissue concentrations are often more reliable.

The following clinical categories were used for grouping analytical data:

Therapeutic: blood-plasma concentrations (in general, trough at steady state) observed following therapeutically effective doses; no or only minimal side effects (drugs); "normal": concentrations associated with no or only minimal toxic effects (other xenobiotics).

Toxic: blood-plasma concentrations which produce toxicity/clinically relevant side effects/symptoms.

Comatose-fatal: blood-plasma (comatose) concentrations and whole blood (fatal) concentrations reported to have caused coma and death, respectively. Whether published data for deaths refer to levels measured ante-mortem or post-mortem (femoral or heart blood) is, however, often unknown.

As no patient interventions were performed and this compilation does not contain data on human experimental research performed by the authors, ethical review board approval is waived and informed consent does not apply.

## Results and discussion

For nearly 1,000 drugs and other xenobiotics, therapeutic ("normal") and, if data were available, toxic and comatose-fatal plasma concentrations and elimination half-lives were compiled in one table (see Additional file 1). The compilation primarily includes data for centrally-active substances, such as hypnotics, sedatives, anxiolytics, antipsychotics, lithium, antidepressants, analgesics, anti-epileptics and stimulants as well as non-steroidal anti-inflammatory drugs (NSAIDs), corticosteroids, antihistamines, antibiotics, antimycotics, antiretrovirals, diuretics, ACE-inhibitors, sartanes, beta blockers, calcium-channel blockers, cardiac glycosides, anti-arrhythmics, anti-asthmatics, and local anesthetics, among others.

In addition, and if data were available, other relevant xenobiotics, such as controlled substances, illegal and recreational drugs, heavy metals and pesticides among others, were listed. To the best of our knowledge, this compilation is the most current and comprehensive single source of data necessary to support clinical decision making in case of acute or chronic intoxications with drugs and other xenobiotics.

When screening the data, it became obvious that for many well known drugs there is insufficient pharmacokinetic data available. However, for current substances, little data are published about intoxications and their plasma concentrations.

In general, therapeutic plasma concentration ranges or concentrations found after therapeutic doses refer to trough levels (C_min_) at steady state. Inter-individual deviation is, however, high. Therefore, any data listed can only be taken as an orientation.

Often, it is not possible to find the threshold between the therapeutic and toxic concentration for the specific patient. This is, for instance, the case if tolerance develops (this is especially true for opioids) or drug/drug-interactions or additional diseases are involved. In order to keep the overall context clear, we preferred not to go into further details.

Data about comatose or even fatal plasma concentrations consciously orient on life threatening or lethal intoxications which occurred at low plasma concentrations so that actual and potential dangers in clinical cases are not being underestimated. Many intoxicated patients survived even with significantly higher concentrations.

It is also difficult to relate the concentrations to the clinical picture because the interval between intake of the drug and drawing a blood sample is generally unknown. In any case, it is more reliable to have the correct concentration measured rather than how much drug/substance has probably been taken. Statements about case histories are often not reliable. And often, it is not known how much drug has been absorbed after intake of charcoal, due to vomiting and/or irrigation of the stomach.

For a variety of data representing lethal cases, it is not known whether ante- or post-mortem, that is, (venous) femoral or heart blood levels were measured. If this information is unknown, we refrained from mentioning this detail.

Elimination half-lives are statistically more reliable than data gathered in case of intoxications. Yet even with this data, substantial deviation can be expected. In addition, most pharmacokinetic parameters are retrieved from healthy subjects after application of relatively low doses. The data indicated generally deal with the terminal elimination half-life, which most of the time is higher than the half-life of the intended biological effect (see annotations in the Additional file 1).

## Conclusions

In case of intoxications, the concentration of the ingested substance and/or metabolite in blood plasma much better predicts the clinical severity of the case when compared to the assumed amount and time of ingestion. Comparing and contrasting the clinical case against the data provided, including the half-life, may support the decision for or against further intensive care. In addition, the data provided are useful for the therapeutic monitoring of pharmacotherapies, to facilitate the diagnostic assessment and monitoring of acute and chronic intoxications as well as to support forensic and clinical expert opinions.

## Key messages

• Acute clinical intoxications are frequent.

• The concentration of the ingested substance better predicts the clinical severity of the case when compared to the assumed amount and time of ingestion.

• The most current and comprehensive single source of data necessary to support the clinical decision making in case of acute or chronic intoxications for nearly 1,000 drugs and other xenobiotics is provided.

• Comparing and contrasting the clinical case against the data provided, including the half-life, may support the decision for or against further intensive care.

## Abbreviations

appr.: approximately; AUC: area under the (plasma concentration-time) curve; BAT: biological tolerance value (in the work area); C_min_: minimum (trough) plasma concentration (usually at steady state); C_max_: maximum (peak) plasma concentration; EM: extensive metaboliser; h: hours; mol wt: molecular weight; NSAID: non-steroidal anti-inflammatory drug; PM: poor (slow) metaboliser; Ref.; reference(s); SD: standard deviation; rec: recombinant; t_max_: time to peak plasma concentration (C_max_); t_½_: in general: terminal elimination half-life (if not stated otherwise); TDM: therapeutic drug monitoring.

## Competing interests

The authors declare that they have no competing interests.

## Authors' contributions

MS had the original idea, critically analysed the available data, reviewed other compilations and textbooks, searched and retrieved the original papers and drafted the manuscript. AS critically analysed the available data, reviewed other compilations and textbooks, and analysed and provided the data of several thousand cases analysed in our toxicological laboratory. MS and AS developed and published the previous compilations. SIB and HA critically reviewed all data, searched and analysed all available review sources, and provided data as well as references for more than 170 new substances including case reports analysed in our toxicological laboratory. All authors read and approved the final manuscript.

## Authors' information

MS started his career in pharmacology and toxicology in 1984 as a PhD-student at the Department of Legal Medicine, University Medical Centre Hamburg-Eppendorf. He is both a board-certified pharmacologist and drug information specialist and the current Chairman of the Drug Commission of German Pharmacist and an Adjunct Professor at the Department of Pharmacology, Goethe-University Frankfurt. SIB, PhD in toxicology, is a board-certified forensic toxicologist at the Department of Legal Medicine, University Medical Centre Hamburg-Eppendorf. HA, PhD in toxicology, is both a board-certified forensic and clinical toxicologist and the current Head of the toxicological laboratories at the Department of Legal Medicine, University Medical Centre Hamburg-Eppendorf. AS, MD and PhD in toxicology, is the Professor Emeritus and former head of the toxicological laboratories at the Department of Legal Medicine, University Medical Centre Hamburg-Eppendorf, Germany.

## Supplementary Material

Additional file 1 **Therapeutic ("normal"), toxic, and comatose-fatal blood-plasma concentrations (mg/L) in man**. A table containing therapeutic ("normal"), toxic, and comatose-fatal blood-plasma concentrations (mg/L) of nearly 1,000 drugs and other xenobiotics in humans, including annotations and references. Click here for file
