# Supplementary material for: Therapeutic and toxic blood concentrations of nearly 1,000 drugs and other xenobiotics
Source: Crit Care. 2012 Jul 26;16(4):R136. doi: 10.1186/cc11441 (PMC3580721; doi:10.1186/cc11441)
Supplement: Additional file 1 — Therapeutic ("normal"), toxic, and comatose-fatal blood-plasma concentrations (mg/L) in man. A table containing therapeutic ("normal"), toxic, and comatose-fatal blood-plasma concentrations (mg/L) of nearly 1,000 drugs and other xenobiotics in humans, including annotations and references. [file cc11441-S1.DOC]

**Therapeutic (“normal”), toxic, and comatose-fatal blood-plasma concentrations (m**g/L) in man

| **Substance** | **Blood-plasma concentration (mg/L)** | | | **t_½_ (h)** | **Ref.** |
| --- | --- | --- | --- | --- | --- |
|  | **therapeutic (“normal”)** | **toxic (from)** | **comatose-fatal (from)** |  |  |
| Abacavir (ABC) | 0.9-3.9^308^ |  |  | appr. 1.5 | [1,2] |
| Acamprosate | appr. 0.25-0.7^231^ | 1^311^ |  | 13-20^232^ | [3], [4], [5] |
| Acebutolol^1^ | 0.2-2 (0.5-1.26)^1^ |  | 15-20 | 3-11 | [6], [7], [8] |
| Acecainide | see (N-Acetyl-) Procainamide |  |  |  |  |
| Acecarbromal(um) | 10-20 (sum) | 25-30 |  |  |  |
| Acemetacin | see Indomet(h)acin |  |  |  |  |
| Acenocoumarol | 0.03-0.1^197^ | 0.1-0.15 |  | 3-11 | [9], [3], [10], [11] |
| Acetaldehyde | 0-30 | 100-125 |  |  | [10], [11] |
| Acetaminophen | see Paracetamol |  |  |  |  |
| Acetazolamide | (4-) 10-20^267^ | 25-30 |  | 2-6 (-13) | [3], [12], [13], [14], [11] |
| Acetohexamide | 20-70 | 500 |  | 1.3 | [15] |
| Acetone | (2-) 5-20 | 100-400; 2000^8^ | 550 | (6-)8-31 | [11], [16], [17] |
| Acetonitrile |  |  | 0.77 | 32 | [11] |
| Acetyldigoxin | 0.0005-0.0008^3^ | 0.0025-0.003 | 0.005 | 40-70 | [18], [19], [20], [21], [22], [23], [24], [25], [26], [27] |
| Acetylsalicylic acid (ASS, ASA) | 20-200^2^ | 300-350^2^ | (400-) 500^2^ | 3-20^2; 37^ | [28], [29], [30], [31], [32], [33], [34] |
| Acitretin | appr. 0.01-0.05^112^ |  |  | 2-4^6^ | [35], [36] |
| Acrivastine | -0.07 |  |  | 1-2 | [8] |
| Acyclovir | 0.4-1.5^203^ |  |  | 2-5^83^ | [37], [3], [38], [39], [10] |
| Adalimumab (TNF-antibody) | appr. 5-9 |  |  | 14^6^ | [40] |
| Adipiodone(-meglumine) | 850-1200 |  |  | 0.5 | [41] |
| Äthanol | see Ethanol |  |  | -^139^ |  |
| Agomelatine | 0.007-0.3^310^ | 0.6^311^ |  | 1-2 | [4] |
| Ajmaline | (0.1-) 0.53-2.21 (?) |  | 5.5^8^ | 1.3-1.6, 5-6 | [3], [42] |
| Albendazole | 0.5-1.5^92^ |  |  | 8-9^92^ | [43], [44], [45], [46] |
| Albuterol | see Salbutamol |  |  |  |  |
| Alcuronium | 0.3-3^353^ |  |  | 3.3±1.3 | [47] |
| Aldrin | -0.0015 | 0.0035 |  | 50-167^6^ (as dieldrin) | [11], [48] |
| Alendronate (Alendronic acid) | < 0.005^322^ |  |  | -^6^ | [49], [50], [51] |
| Alfentanil | 0.03-0.6^4^ |  |  | 0.6-2.3^96^ | [52], [53], [54], [55] |
| Alfuzosine | 0.003-0.06 |  |  | 3-9 | [8] |
| Alimemazine (Trimeprazine) | 0.05-0.4 | 0.5 | 1-3.2 | 8 | [56], [57] |
| Alizapride | 0.1-2 |  |  | 2-3 | [15] |
| Allobarbital | 2-5 | 10 | 20 | 40-48 | [47], [11], [58] |
| Allopurinol^354^ | 2-19 |  |  | 0.5-3 | [11] |
| Almotriptan | 0.05-0.07 |  |  | 3-4 | [47] |
| Alphaprodine | 0.87-1 |  |  | 1.6-2.6 | [11] |
| Alprazolam | 0.005-0.05 (-0.08)^65^ | 0.1-0.4 | 252 | 6-20 | [56], [3], [59], [60], [61], [62], [63] |
| Alprenolol^48^ | 0.025-0.14 | 1-2 | 40-48 | 2-7 | [7] |
| 4-Hydroxyalprenolol | 0.04-0.06 |  |  |  | [8] |
| Aluminium | < 0.005^234^ | 0.05-0.15 | 4.4^8^ | appr. 0.5 | [64], [65], [66], [11] |
| Amantadine | (0.06-) 0.2-0.6 (-1) | 1; 2.4^8^ | 2.1-4.8; 21^8^ | 9-15 | [48] |
| Amfebutamone | see Bupropion |  |  |  |  |
| Amikacin | 10-25^76^ | 30 |  | 2-3 | [67] |
| Aminobenzoic acid | 300-600 | 600 |  |  | [66] |
| Aminoglutethimide | (0.05-) 7.5-25 |  |  | 10-15 | [3], [8], [11] |
| Aminophenazone | 10-20 |  |  | appr. 2-4 | [15] |
| 4-Aminopyridine (Fampridine) | 0.025-0.075 | 0.14^8^; 0.2 |  | 3-3.5 | [68], [10], [11] |
| 5-Aminosalicylic acid (5-AS, 5-ASA) | see Mesalazine |  |  |  |  |
| Amiodarone^261^ | (0.5-) 1-2 (-2.5) | 2.5-3 |  | 30-120^6^ | [69], [64], [3], [70], [71] |
| Amisulpride | 0.1-0.4 | 0.64^311^ | 9.3^8^; 41.7^8^ | 12-20 | [4], [72], [15], [8] |
| Amitriptyline^7; 48^ | 0.05-0.3 | 0.5-0.6 | 1.5-2 | 30-50 | [73], [74], [56], [75], [76], [77], [78], [79], [80], [81], [82], [83] |
| Amlodipine | 0.003-0.015 | 0.088^8; 165^ | 0.1-0.2^8; 166^ | 34-50 | [84], [85], [86] |
| Ammonia | 0.5-1.7 |  |  |  | [11] |
| Amobarbital | 1-5 | (5-6) 10-30 | 13-96 | 15-30 | [87], [48] |
| Amodiaquine | -0.05^270^ |  |  | -^270^ | [88] |
| Amoxapine | 0.18-0.6^151^ | 3 | 5 | 8 | [9] |
| Amoxicillin | 0.5-1 (5-15) |  |  | 1-2 | [15] |
| Amphetamine | 0.02-0.1 | 0.2 | 0.5-1 | 4-8 (7-34)^344^ | [89], [66] |
| Amphotericin B | (0.1-) 0.2-3 | (3-) 5-10 |  | 24-48^110^ | [47], [90], [91], [92], [93] |
| Ampicillin | 0.02-2 (2-20) |  |  | 1 | [67] |
| Amrinone | 1-2 (-4) |  |  | 3-12 | [3], [94], [95] |
| Amsacrine | (0.1-) 1-5.5 |  |  | 5-7 | [96] |
| Anileridine | < 0.5 |  | 0.9^8^ |  | [11], [48] |
| Aniline | -0.02^328^ (urine) | 0.13^8; 355^ | 6 | (2-) 3-4 (-7) | [11], [48], [97], [47], [98], [99] |
| Antimony | -0.01^329^ | 0.2 |  |  | [64], [100] |
| Antipyrine | see Phenazone |  |  |  |  |
| Apomorphine | 0.002-0.02^204^ |  |  | appr. 0.75 | [3], [10] |
| Aprindine^48^ | 1-2 | 2-3 |  | 13-50 |  |
| Aprobarbital | 4-20 | 30-40 | 50 | 14-34 | [47], [11], [58] |
| Aripiprazole | 0.15-0.5 | 1^311; 345^ |  | 60-80 | [101], [4], [102] |
| Arsenic | 0.002-0.07^283^ | 0.05-0.25 | 9-15 |  | [103], [64], [87], [11], [100] |
| Asenapine | 0.002-0.005 | 0.01^311^ |  | 24 | [4] |
| Articaine | < 1.5-2 (?) |  |  | 0.3 (-1) | [104] |
| Ascorbic acid (Vitamin C) | 4-15 |  |  | -^6^ | [105], [3], [106], [107],[108] |
| Astemizole | 0.002-0.05^43^ | 14^8^ |  | appr. 20^6; 42; 43^ | [47], [3], [109] |
| Atazanavir (ATV) | > 0.15^293^ |  |  | 6.5-8.6 | [110], [2], [111] |
| Atenolol | 0.1-1 (-2)^77^ | 2-3 | 27^8^ | 4-14^9^ | [3], [42], [7], [112], [64] |
| Atomoxetine | 0.2-1^317^ | 2^311^ |  | appr. 4^356^ | [4] |
| Atovaquone | 13.9 ± 6.9 (> 15) |  |  | 2-3^6^ | [113] |
| Atracurium(besylate) | 0.1-0.5 (-5) |  |  | appr. 0.5 |  |
| Atropine | 0.002-0.025^155^ | 0.03-0.1 | 0.2 | 2-6.5, 13-38 | [66], [114], [11] |
| Azapropazone (Apazone) | 40-90 |  |  | 8-24 |  |
| Azathioprine^10^ | 0.05-2 |  |  | 1-4^11^ |  |
| Azelastine | 0.002-0.003(-0.01) |  |  | 22-25 | [15] |
| Azithromycin | appr. 0.04-1 |  |  | 50-60 (2-4^6^) | [115], [116], [117], [118], [119] |
| Aztreonam | 1-10 (50-250) |  |  | 1.5-2 | [11] |
| Baclofen | 0.08-0.4 (-0.6) | 1.1-3.5 | 6-9.6 | 6.8±0.7 | [47], [9], [11] |
| Bambuterol | see Terbutaline |  |  |  |  |
| Barbexaclone | active metabolite = phenobarbital (see Table) |  |  |  | [12] |
| Barbital | 2-20 | 20-50 | 50 | 57-120 |  |
| Barium | -0.001 |  |  | 10-18 | [47] |
| Bendrofluazide | 0.05-0.1 |  |  | appr. 3 | [42] |
| Benoxaprofen | -50 |  |  | 19-39 | [3] |
| Benperidol | 0.001-0.01 | 0.02^311^ |  | 4-8 | [4], [15], [8] |
| Benzbromarone | 2-10 |  |  | 2-4 |  |
| Benzene | -0.0002^271^ |  | 0.95 | 9-24 | [47], [58] |
| Benzonatate |  | 2.5 |  | appr. 1-3 | [8] |
| Benzoylecgonine^357^ | -0.1 |  | 1 | 4-5 | [8] |
| Benzphetamine | 0.025-0.5 | 0.5 | 14^8^ |  | [47], [8], [11] |
| Benztropine | 0.01-0.18 | 0.05 | 0.2-0.7 |  | [15], [8], [11] |
| Benzyl alcohol |  | 18^8, 194^ |  | -^195^ | [3], [120], [121] |
| Benzylpenicillin | 1.2-12 |  |  | 1 | [67], [41] |
| Bepridil | 0.6-2.5 |  |  | 33-42 (30-130) | [122], [8] |
| Beryllium | -0.0003 |  |  |  | [58] |
| Betacarotene | 4-6^196^ |  |  |  | [123], [124] |
| Betaxolol | 0.005-0.05 |  | 36^8^ | 14-22 | [47], [125], [7] |
| Bethanidine | 0.02-0.5 |  |  | 9-10 | [3], [58] |
| Bevantolol | 0.2-2 |  |  | 2 | [15], [8] |
| Bezafibrate | -15 |  |  | 2 | [3] |
| Bicalutamide | 1.5-17.5 (-25)^163^ |  |  | (3-) 7-10^6^ | [126] |
| Biperiden | 0.05-0.1? |  | 0.25^8^ | 18-24 | [56] |
| Bismut(h) | < 0.05 (-0.1) | 0.05-0.1 |  | -^6^ | [47], [64] |
| Bisoprolol | 0.01-0.1 |  |  | 10-12 | [7] |
| Bopindolol | 0.001-0.015^54^ |  |  | 4-8^54^ | [7] |
| Borate | 0-7 | 20 | 200 | 12-27 | [47], [66] |
| Boron | 0.8-6 | 20-50 | 50-150 |  | [58] |
| Bornaprine | 0.0007-0.0072^313^ | 0.014^311^ |  | appr. 30 | [4] |
| Brallobarbital (Brallobarbitone) | 4-8 | 8-10 | 15 | 20-40 |  |
| Bretylium | 0.8-2.4 |  |  | 6-11 | [3] |
| Brodifacoum |  | 0.02 | 0.03-0.17; acute: 3.9^8^ | 20-60^6^ | [58], [127] |
| Bromadiolon |  | 0.02 |  | 3-6^6^ (early), 10-24^6^ (late phase) | [11], [58] |
| Bromazepam | (0.05-) 0.08-0.2 | 0.3-0.4 | (1-) 2 | 8-22 | [59] |
| Bromide | 75-100 (-300) | 500-1500; 3000^8; 242^ | 2000 | 12-13^6^ | [58], [128], [129], [130] |
| Bromisoval | 10-20 | 30-40 |  | appr. 4^28, 105^ |  |
| Bromocriptine | 0.0001-0.0003 (-0.004)^314^ | 0.008^311^ |  | appr. 38 | [4] |
| Bromoxynil |  | 20 |  |  | [58] |
| Bromperidol | 0.012-0.02 | 0.03^311^ |  | 20-36 |  |
| Brompheniramine | 0.005-0.015 | 0.2^8^ |  | 2-10 (-20) | [15] |
| Brotizolam | 0.001-0.01 (-0.02) | 0.02^311^ | 0.01-0.03^8^ | 4-10 | [47], [131] |
| Budipine | appr. 0.1-0.3 |  |  | 30 | [15] |
| Buflomedil | appr. 0.2-0.5 (-1.0) | 15-25 | 25-50; 275^8^ | 2-4 | [42], [11], [58] |
| Bunitrolol | 0.001-0.015 |  |  | 2-6 | [7] |
| Bupivacaine | (0.25-) 0.5-1.5 (-2) | 2-4 |  | 0.5-3 | [123], [132], [133], [134] |
| Bupranolol | -^44^ |  |  | 2-4 | [7] |
| Buprenorphine^340^ | 0.0005-0.005 (-0.01)^285^ | 0.03-0.1^339^ | 0.008-0.029 | 3-5 (i.v.); 18-49 (sublingual); appr. 19 (buccal) | [47], [135], [123], [136], [137], [138], [139], [140], [141] |
| Bupropion (Amfebutamone) | 0.01-0.02; 0.05-0.1^152^ | 1.2-2^246^ | 4^8^; 4.2^8^; 7.3^8^ | (4-) 10-20 | [47], [142], [143], [4], [144], [145], [146], [80], [147], [148] |
| Buspirone^312^ | 0.001-0.004 | 0.008^311^ |  | 2-3 | [4] |
| Busulfan | > 0.9^291^ |  |  | 2-4 | [149], [150], [151], [152], [153], [154] |
| Butabarbital | see Secbutabarbital |  |  |  |  |
| Butalbital | 1-5 | 10-15 | 15-30 | 30-40 | [58] |
| Butanone | -10 | 500 |  |  |  |
| Butaperazine | 0.02-0.3 (-0.7) |  |  | 12 | [15] |
| Butorphanol | 0.0006-0.002 |  |  | 4-9 | [15], [8] |
| Butriptyline | 0.07-0.15 | 0.4-0.5 |  |  | [15] |
| Butylscopolamine | -0.7 |  |  | 4-5 | [8] |
| Cabergoline | 58-144 pg/mL^315^ | 390 pg/mL^311^ |  | 63-68 | [4] |
| Cadmium | <0.0003-0.0065 | 0.015-0.05 |  | appr. 16 years | [47], [58], [100] |
| Caffeine (Coffein) | (2-) 4-10 | 15-20 | 80-180 | 2-10 | [47], [123], [155], [156], [157], [64] |
| Calcifediol | 0.01-0.05 |  |  |  | [66] |
| Camazepam | 0.1-0.6 | 2 |  | 20-24 | [59] |
| Camphor |  | 0.3-0.4 | 1.7 | 2-8 | [47], [15], [8], [158] |
| Candesartan | 0.08-0.18 |  |  | 5-7 | [15] |
| Canrenone | see Spironolactone |  |  |  |  |
| Captopril | 0.05-0.5 (-1) | 5-6 | 60 | 1-2 | [159], [84], [42] |
| Carazolol^23^ | -0.015 |  |  | 9 | [7] |
| Carbachol | appr. 0.01? |  | 3.6^8; 287^ |  | [3], [160] |
| Carbamazepine^12^ | 2-8 (4-12) | 10 | 20 | 12-60 (7-35)^140^ | [161], [162], [56], [163], [164], [165], [166], [12] |
| Carbaryl |  | 5 | 6-27 |  | [47], [167] |
| Carbenoxolone | appr. 5-30 |  |  | 8-20 | [66] |
| Carbidopa | 0.02-0.2^316^ | 0.4^311; 316^ |  | 2 | [4] |
| Carbimazole | 0.5-3.4^95^ |  |  | 3-6^95^ |  |
| Carbinoxamine | appr. 0.02-0.04 |  |  | appr. 10-15 | [168] |
| Carboc(h)romene | 0.8-2.4 (-3) |  |  | 0.2-1.5 |  |
| Carbon monoxide | -^200^ | 25-30 % | 50-60 % |  | [123], [10], [58] |
| Carbon tetrachloride | -0.07 | 0.12^8^ ; 7.1^8 ; 269^ ; 11^8^ ; 10-50 | 100-200 | Appr. 24 ; 42.6^8^ | [169], [58], [170], [171], [172], [173] |
| Carboplatin | max. 10-25 |  |  | 2.5-6^106^ |  |
| Carbromal(um)^13^ | 2-10 | 15-20 | 40 | 7-15 |  |
| Carisoprodol | 10-30 | 40; 30-50^104^ | 50^8^; 110^104^ | 8 | [56], [58] |
| ß-Carotine | see Betacarotene |  |  |  |  |
| Carteolol | 0.01-0.1 |  |  | 3-7 | [7] |
| Carvedilol | appr. 0.02-0.15 (-0.3) |  |  | 6-10 | [3], [95], [8] |
| Cathine | appr. 0.71^370^ |  |  | 3-8 | [47] |
| Cefaclor | 13-35 (i.v. -900) |  |  | 0.5-1 (-2) | [3], [8], [11] |
| Cefadroxil | -30 |  |  | 1-2 | [8] |
| Cefalexin | -65 |  |  | 1-1.5 | [41] |
| Cefaloridine | 20-80 |  |  | 1.5 | [41] |
| Cefalotin | See Cephalotin |  |  |  |  |
| Cefamandole | 1-5 (10-40-150) |  |  | 0.5-1.2 | [41], [58] |
| Cefazolin | -150 |  |  | 1.5-2 | [67], [41] |
| Cefdinir | -4 |  |  | 16 | [8] |
| Cefepime | -160 |  |  | 2 | [8] |
| Cefetamet | -7 |  |  | 2-3 | [8] |
| Cefixime | -7 |  |  | 3-4 | [8] |
| Cefmenoxime | -200 |  |  | 1-2 | [8] |
| Cefodizime | -400 |  |  | 2-4 | [8] |
| Cefoperazone | -250 |  |  | 1-2 (-5) | [174] |
| Cefotaxime | 0.5-2 (10-50; i.v. -225) |  |  | 1-1.5 | [67] |
| Cefotetan | 65-90 |  |  | 3.5 | [41] |
| Cefotiam | -150^71^ |  |  | 0.7-1.5 (-2) | [41], [175] |
| Cefoxitin | -150 |  |  | 0.7-1 | [41] |
| Cefpodoxime | -7 |  |  | 2-3 | [8] |
| Cefsulodin | 20-100 |  |  | 1.6-1.9 | [3], [8], [11] |
| Ceftazidime | 20-40 (50-200) |  |  | 1-4 | [3], [8], [11] |
| Ceftibuten | appr. 3-20 |  |  | 2-4 |  |
| Ceftizoxime | 40-160 |  |  | 6-9 | [41] |
| Ceftriaxone | 15-75 |  |  | 6.5-8.5 | [67] |
| Cefuroxime | 0.5-1 (10-60 ; i.v. -180)^243^ |  |  | 1.1-1.3 | [3], [176], [177], [10], [58] |
| Celecoxib^48^ | 0.36-0.8^371^ |  |  | 11-16 | [47] |
| Celiprolol | 0.05-0.5 (-1) |  |  | 3-6 | [58] |
| Cephalothin (Cefalotin) | -30 |  |  | 0.5-0.6 | [3], [41] |
| Cerivastatin | 0.002-0.04 |  |  | 1.5-3 | [15], [8] |
| Cetirizine | appr. 0.02-0.3 | 2-5 |  | 7-9 | [47], [15], [8] |
| Chinidine | see Quinidine |  |  |  |  |
| Chinine | see Quinine |  |  |  |  |
| Chloralhydrate^14^ | 1.5-15 | 40-50 | 60-100 | 8-30 |  |
| Chlorambucil | 0.15-0.3 (-1.0) |  |  | 1.5-3 | [15] |
| Chloramphenicol | 5-10 (-15)^59^ | 25 |  | 2-6 | [178], [11], [58] |
| Chlordane | -0.001 | 0.0025 | 1-7 | 88^6^ | [11], [58] |
| Chlordecone |  | 0.5 |  | 63-148^6^ | [47] |
| Chlordiazepoxide^15^ | 0.4-3 | 3.5-10 (-15); 20.5^8^ | 20^8^; 26^8^ | 6-27 | [179], [47], [56], [180], [11], [58] |
| Chlormethiazole | see Clomethiazole |  |  |  |  |
| Chlormezanone | (3-) 5-9 (-14) | appr. 20 | 18^8^; 53^8^ | 20-30 | [43], [56], [181] |
| Chlorobutanol |  | 75 |  |  | [66] |
| Chloroform | 20-50 | appr. 70 | 33^8^; 64^8^; 69^8^; 91^8^ | 1.5 | [47], [11], [58], [182] |
| Chlorophacinone |  | 0.1 |  | 6-23^6^ |  |
| Chloroquine | 0.02-0.5 | 1 | 3 | dose-dependent^6^ | [183], [56], [30] |
| Chlorothiazide | appr. 6 |  |  | 0.5-2 | [3], [11], [58] |
| Chlorphen(ir)amine | 0.003-0.017 |  | 1.1^8^ | (12-) 15-25 (-43)^358^ | [47], [3], [11], [58] |
| Chlorpromazine^66^ | 0.03-0.1 (-0.5) | 1-2 | 3-4 | 10-30 | [56], [3], [184], [185] |
| Chlorpropamide | 30-150 | 200-750 |  | 25-60 | [66], [58] |
| Chlorprothixene | 0.02-0.3 | 0.4 | 0.8 | 8-12 | [47], [4] |
| Chlorpyrifos |  | 0.2 | 1.3 (0.4-3.5)^276^ | 27 | [47], [186] |
| Chlortalidone | 0.15-0.3 (-1.4) | appr. 2 |  | 44-48 (35-70) | [47] |
| Chlortetracycline | 1-5 (-10) | 30 |  | 5-6 | [9], [13], [14] |
| Chromium | -0.00035 |  | 32^8^ | 3-4 years | [47] |
| Cibenzoline | 0.2-0.4 (-0.9) | (0.5-) 1 |  | 7-8^83^ | [3], [187] |
| Cicletanine | appr. 1-2 |  |  | 5-23 | [3], [8] |
| Ciclosporine A (CsA) | < 0.1-0.15-0.25 | 0.3-0.4^16^ |  | 10-27^169^ | [188], [189], [190], [191], [192], [193] |
| Cidofovir | appr. 7-43 |  |  | 2.5 | [8], [3] |
| Cilazapril(-)at | 0.003-0.09 |  |  | 30-50 | [8] |
| Cimetidine | 0.25-3 (0.75-4) | 30-50 | 110^8^ | 1.5-4 | [194], [195] |
| Cinnarizine | 0.04-0.33 | 7.4^8^ |  | 12-34 | [47] |
| Cinoxacin | appr. 15 |  |  | 1.5-4 |  |
| Ciprofloxacin | 2.5-4 | 11.5^8^ |  | 3-6 | [196], [197], [198], [199] |
| Cisaprid | 0.04-0.08 |  |  | 6-12 | [8] |
| Citalopram | 0.05- 0.11 | 0.22^311^ | 5-6^160^ | appr. 33^170^ | [8], [200], [201], [202], [4], [203], [204], [205],[206], [207] |
| Cladribine | appr. 0.006 |  |  | 0.1-0.2 (6.4-19.7) | [208], [209], [210] |
| Clarithromycin | appr. 0.2-2 |  |  | 3-7^217^ | [115], [211], [8], [118], [212], [213] |
| Clemastine | appr. 0.001-0.002 (?) |  |  | appr. 8 | [8], [214] |
| Clenbuterol | 0.0003-0.0006 | 0.003^8^ |  | 30-35 | [47] |
| Clindamycin | appr. 0.5 |  |  | 2-3 |  |
| Clobazam^17^ | 0.03-0.3 | 0.5^311^ |  | 18-42 | [47], [4], [59], [215] |
| Clobutinol | appr. 0.05-0.2 |  |  | 23-34 | [3], [8] |
| Clodronate (Clodronic acid) |  |  |  | -^6^ | [49] |
| Clofibrate | 50-250 |  |  | 10-18 |  |
| Clomethiazole (Chlormethiazole) | 0.1-5 | (2.8-) 4-15 | 50 | 3-7 | [3], [4], [216], [9], [13],[217] |
| Clomipramine^48, 85^ | (0.02-) 0.09-0.25 (-0.4)^226^ | 0.4-0.6 | 1-2 | 20-26^86^ | [218], [219], [220], [76], [221], [222], [80], [223] |
| Clonazepam | (0.004-) 0.02-0.08^150^ | 0.1 |  | 20-60 | [161], [60], [224] |
| Clonidine | 0.001-0.002 (-0.004) | 0.025-0.05 (0.009^8^) | 0.23^8^ | 5-20 | [47], [225], [226], [227] |
| Clopenthixol | 0.002-0.015 | 0.1-0.3 |  | 15-25 | [8], [11] |
| Clopidogrel | 0.001-0.006 |  |  | 1.4-3.6 | [47] |
| Clorazepate^15^ | see Nordazepam |  |  | 1-2 | [164] |
| Clotiazepam | 0.1-0.7 |  |  | 3-15 |  |
| Cloxacillin | 5-30 (-85) |  |  | 0.5-1 (0.3-2) |  |
| Clozapine^136^ | (0.1-) 0.35-0.6 (>0.35 ?) | 0.6-1 (9.5^8^) | 1.2^8^; 2^8^; 5.2^8^ | 6-14 | [101], [228], [229], [230], [231], [232], [233], [234], [72], [235], [236], [237], [238], [239], [240] |
| Cobalt | 0.0001-0.0022 |  |  | 2 (early); 38 (late phase) | [47], [11], [58] |
| Cocaine | 0.05-0.3 | (0.25-) 0.5-1 | 0.9-2.1 | 0.5-1^18^ | [47], [241], [242], [13], [14], [58] |
| Codeine^48^ | 0.03-0.25 | 0.5-1^339^ | 1.8 | 3-4 | [3], [243], [244] |
| Coffein(e) | see Caffeine |  |  |  |  |
| Colchicine | 0.0003-0.0025 | 0.005 (0.019^8^) | 0.009^8^; 0.024^8^ | 11-32^143^ | [245], [246], [247], [248], [10] |
| Colistin | 1-5 |  |  | 2-5 |  |
| Copper | 0.6-1.5 | 2 | 5 | 26^6^ | [66] |
| Cotrimoxazole | see Sulfamethoxazole and Trimethoprim |  |  |  |  |
| Coumatetralyl |  | 0.12^8^ |  |  | [249] |
| Cresol (Methylphenols) |  | appr. 50 | 120 |  | [58] |
| Cromolyn (Cromoglycate) | appr. -0.01 |  |  | 1-1.5 | [3] |
| Cyanide | -^177^ | 0.5 | 1-3 | appr. 19^184^ | [3], [250], [251], [252], [253], [254], [255], [130], [256] |
| Cyclizine | 0.1-0.25 | 0.75-1 | 15 | 24 | [11], [58] |
| Cyclobarbital | 2-6 | 10 | 20 | 8-17 |  |
| Cyclobenzaprine | appr. 0.003-0.04 | 0.4 |  | 18 (9-40)^253^ | [11], [58], [257] |
| Cyclohexane | -0.4 |  |  |  |  |
| Cyclophosphamide | 10-25 |  |  | 4-8 (1.3-16) | [3], [8] |
| Cyclopropane | 80-180 |  |  |  | [11], [58] |
| Cyclosporine | see Ciclosporine |  |  |  |  |
| Cyproheptadine | appr. -0.05 |  | 0.47^8^ | 8-9 | [47], [3] |
| Cyproterone acetate |  |  |  | 30-40 | [126] |
| Cysteamine | appr. > 20 µmol/L^282^ |  |  | appr. 1 | [258], [3] |
| Cytarabine | 0.05-0.5 |  |  | 0.1-0.2 (1.9-2.5) | [209], [9] |
| 2,4-D | see 2,4-Dichloro-phenoxyacetic acid |  |  |  |  |
| Danazol | appr. -0.2 |  |  | 4.5 | [3] |
| Dantrolene | (0.1-) 0.4-1.5 (-3) |  |  | 4-12 | [9], [3], [259] |
| Dapsone^48^ | 0.5-2 | 10 | 18^8^ | 25-31 | [260], [261] |
| Darunavir (DRV) | > 3.3 (1.255-7.368)^301^ |  |  | appr. 15 | [262], [110], [2] |
| DEET | see N,N-Diethyl-3-methylbenzamide |  |  |  |  |
| Deferoxamine (Desferrioxamine) | 3-15 |  |  | 4-6 |  |
| Demoxepam | 0.5-0.74 | 1 | 2.7 |  | [66] |
| Desipramine ^48, 69^ | 0.01-0.5 (0.12-0.25) | 0.5-1 | 3 | 15-25^70^ | [74], [263], [78], [79], [80], [264] |
| Desloratadine^372^ | 0.002-0.006^373^ |  |  | 17-27 | [47] |
| Desmethyldiazepam (N-Desmethyldiazepam) | see Nordazepam |  |  |  | [59] |
| Desvenlafaxine | 0.1-0.4 | 0.6^311^ |  | 11 | [4] |
| Detajmium | 0.01-0.7 |  | 1.8^8^ | 13-14 | [8] |
| Dexamethasone | appr. 0.05-0.265^247^ |  |  | 2.5-9.5 | [3], [265] |
| Dexfenfluramine^351^ | appr. 0.03-0.06 | 0.15-0.25 |  | appr. 18 | [66], [11], [58] |
| Dexketoprofen^374^ | appr. 3.7 |  |  | 0.5-2 | [47] |
| Dexmethylphenidate^342^ | 0.013-0.023^318^ | 0.044^311; 318^ |  | appr. 2 | [4] |
| Dextromethorphan^48^ | 0.01-0.04 | 0.1 | 3 | 2-4 | [266], [267], [268], [269] [270] |
| Dextromoramide^350^ | 0.075-0.15 | 0.2^339^ | 0.9 | 1.5-4-7 | [47], [271], [8], [272], [58] |
| Dextropropoxyphene^305^ | 0.05-0.3 (-0.5) | 0.6-1 | 1-2 | 10-30 | [30], [48] |
| Diacetylmorphine or Diamorphine (DAM) | see Heroin (and Morphine) |  |  | 2-5 min | [273], [274], [275], [276], [277], [278], [279], [280], [281] |
| 3,4-Diaminopyridin (DAP) | < 0.04^213^ | 0.1 (?) |  | 0.3-2^214^ | [282] |
| Diazepam^19^ | 0.1-2 (-2.5) | 3-5 |  | 24-48 | [241], [283], [284], [3], [285], [59], [224], [13], [14], [286] |
| Diazinon |  | 0.05-0.1 (-0.5) |  |  | [58] |
| Diazoxide | 10-20 (-50) | 50 (-100) |  | 20-36 (-48) | [3], [84], [42], [10], [58] |
| Dibenzepine | 0.025-0.15 (0.1-0.5) | 3^359^ | 18^359^ | 3.5-5 | [11], [58] |
| Dichloromethane |  | 200 | 280 | 0.6 (early); 4-8 (late phase) | [47] |
| 2,4-Dichlorophenoxyacetic acid (2,4-D) | - | appr. 100 | 200; 392^8^; 720^8^ | appr. 18^182^ | [3], [11], [58] |
| Dichlorvos |  |  | 29 | 0.16 | [47] |
| Diciclomine | see Dicyclomine |  |  |  | [8] |
| Diclofenac | 0.5-3 | 50; 60^8^ |  | 1-2 | [287], [288], [289] |
| Dicoumarol | 8-30 | 40-50 |  | 1-4^6^ | [8], [11] |
| Dicyclomine (Dicycloverin) | -0.1 | appr. 0.2 | 0.5 | 1.8-2 | [8] |
| Didanosine (DDI) | appr. 1-30 µmol/L |  |  | appr. 1.4 | [290], [291], [38], [2], [3] |
| Dieldrin | -0.0015 | 0.15-0.3 |  | 2-12 months | [47], [11], [58] |
| Diethylcarbamazine | > 0.8-1.0 |  |  | 4-15 | [292] |
| Diethylene glycol | - | 200-500 | 2000 | 3-4 | [47] |
| N,N-Diethyl-3-methylbenzamide (N,N-Diethyl-m-toluamide; DEET) | - | >1 mmol/L |  | appr. 2.5 |  |
| Diethylpentenamide (Valdetamide) | 2-10 | 20 | 45 | 6-7 |  |
| Diethylpropion | 0.003-0.007 (-0.2) | 2 | 5.4^8^ | 4-8^23^ | [3], [8], [58] |
| Difenacoum |  | 0.5 |  | 11-42^6^ | [47] |
| Diflunisal | 40-100 (-200) | 300-500 | 600 | 5-12^83^ | [3], [9], [58], [293] |
| Digitoxin | 0.01-0.025 | 0.03 | 0.04 | 140-200 | [294], [295] |
| Digoxin | 0.0005-0.0008 (-0.002) | 0.0025-0.003 | 0.005 | 40-70 | [18], [19], [20], [21], [22], [23], [24], [25], [26], [27] |
| Dihydralazine | see Hydralazine |  |  |  |  |
| Dihydrocodeine | 0.03-0.25 | 0.5-1^339^ | 2 | 3-4 | [14], [58] |
| Dihydroergotamine | 0.001-0.01 |  |  | 7-9 (> 30?) | [3], [8] |
| Diltiazem | 0.03-0.13 (-0.25)^157^ | 0.8-1 | 2-6; 7^8^; 8^8^ | 2-6 (4-9) | [3], [84], [58] |
| Dimenhydrinate | see Diphenhydramine |  |  |  |  |
| Dimethadione^360^ | (350-) 700-1000 | 1000 |  | 5-10^6^ | [3], [8], [12], [11] |
| Dimethindene | 0.01-0.05 |  |  | appr. 6 | [3], [15], [8] |
| Dimethoate |  |  | 355.5 (160-674)^277^ |  | [186] |
| N,N-Dimethyltryptamine | 0.001-0.1 |  |  |  | [58] |
| 4,6-Dinitro-2-methylphenol |  | 40 |  |  |  |
| Dinitro-O-cresol (DNOC) | 1-5 | 30-60 | 75 |  | [58] |
| Diphenhydramine | 0.05-0.1 (-1) | 1-2(-4) | 5-10 | 4-10, 20-60 | [296], [297], [114], [58] |
| Diphenoxylate | appr. 0.01 |  |  | 2-3 |  |
| Dipipanone | appr. -0.05 | 0.2 |  |  |  |
| Diprophylline | see Dyphylline |  |  |  |  |
| Dipyridamole | 0.1-1.5 | 4 |  | 11-13 | [9], [13], [14] |
| Dipyrone | see Metamizole |  |  |  |  |
| Diquat |  | 0.1-0.4 |  |  | [11] |
| Disopyramide | 2-7^78^ | 8 |  | 5-8 | [70], [11], [58], [64] |
| Disulfiram | 0.05-0.4 | 0.5^311^ ; 5 | 8 | appr. 5-7 |  |
| Dixyrazine | appr. 0.3^249^ |  | 5.5^8^; 9.4^8^ |  | [4], [298] |
| Domperidone | appr. 0.01-0.1 |  |  | 12-16 | [3], [299], [300], [301], [302] |
| Donepezil^207^ | appr. 0.03-0.075 | 0.075^311^ |  | 70-100 | [4], [303], [304] |
| Dothiepin (Dosulepin)^20^ | 0.02-0.1 | (0.3-) 0.8 | 1 | 11-40 | [305], [306] |
| Doxacurium | 0.01-0.3 |  |  | 1-2^83^ |  |
| Doxapram | (1.5-) 2-5 | 9^268^ |  | 2.4-9.9 | [307], [3], [11], [58] |
| Doxazosin | 0.01-0.15 |  |  | 10-22 | [95] |
| Doxepin^21^ | 0.01-0.2 (0.03-0.1) | 0.5-1 | 2-4 | 8-25 | [308], [73], [309], [77], [310], [80], [264] |
| Doxorubicin (Adriamycin) | 0.006-0.02 |  |  | 20-48 | [3], [8], [58] |
| Doxycycline | 1-5 (-10) | 30 |  | 7-20 |  |
| Doxylamine^289^ | 0.05-0.2 | 1-2 | 5 | 9-11 | [47], [311], [87] |
| Dronabinol (Delta-9-tetrahydrocannabinol, THC) | 0.005-0.01 (-0.05)^137^ |  |  | 50-100 | [241], [312], [313] |
| Droperidol | appr. -0.05 |  |  | 1.5-2.5^237^ | [3], [314] |
| Drotrecogin alfa | mean 0.072 |  |  | 1.6 | [315] |
| Duloxetine | 0.03-0.12 | 0.24^311^ |  | 9-19 | [4], [223] |
| Dyphylline | 6.5-14 (-20) | 40 |  | 2 | [11], [58] |
| Edrophonium | 0.15-0.2 | appr. 0.15 |  | 1.3-2.4 | [3], [316], [58] |
| Efavirenz (EFV) | > 1.0^297^ |  |  | 40-55 | [110], [2], [111] |
| Eletriptan | 0.06-0.23 |  |  | 3-7 | [47] |
| Enfuvirtide | 2.6-3.4 |  |  | 3-4 |  |
| Emetine | 0.005-0.075 | 0.5 | 2.4^8^ | 24-48 | [8] |
| Enalapril^52^ | 0.01-0.05 (-0.1) |  |  | 8-11 | [159], [84] |
| Encainide^48^ | -^175^ |  |  | 1.5-3.5^176^ | [3] |
| Endrin | -0.003 | 0.01-0.03 |  |  | [58] |
| Enoxacin | 1-4 |  |  | 3-6 | [93] |
| Enoximone | ≥ 0.2 (3-4 ?) |  |  | 4-7 | [3], [95], [8] |
| Enprofylline | 1-5 | 10 |  | appr. 2 | [8] |
| Entacapone | 0.4-1.0 (-7.0) |  |  | (0.5-) 1.5-3.5 | [3], [15], [8] |
| Ephedrine | 0.02-0.2 | 1 | 5^8^ | 3-11 | [269], [87], [8], [57], [58] |
| Epirubicin | 0.01-0.05 |  |  | 24-52 |  |
| Eprosartan | 0.4-1.0 (-1.85) |  |  | 5-9 | [3], [15], [8] |
| Eptastigmine | 0.0002-0.006 |  |  | appr. 1 | [8] |
| Ergotamine | 0.00036-0.00042^375^ | 0.00082^376^ |  | 1.5-2.5 | [47] |
| Erythromycin | 0.5-6 (peak 4-12) | 12-15 |  | 1-3 | [11], [58] |
| Escitalopram^343^ | 0.015-0.08 | 0.16^311^ |  | 26.3±10.8 | [4], [223] |
| Esmolol | 0.15-2 |  |  | 4-16 min | [7] |
| Estazolam | 0.055-0.2 |  |  | 10-24 | [3], [58] |
| Eszopiclone^341^ | appr. 0.087 |  |  | 4-9 | [47] |
| Et(h)amsylate | 15-20 |  |  | 2.5-4 |  |
| Ethacrynic acid | 0.05-0.1 |  |  | 1-4 | [3], [9] |
| Ethadione | 500-1000 | 1000 |  |  | [8] |
| Ethambutol | 0.5-6.5 | 6-10 |  | 2.5-3.5 | [317], [58] |
| Ethanol |  | 1000-2000 | 3500-4000 | -^139^ |  |
| Ethchlorvynol | 0.5-8 | 20 | 50 | 10-25 (-35) | [3], [58] |
| Ethinamate | 1.5-10 | 50-100 | 200^8^ | appr. 2 |  |
| Ethosuximide | 30-100 (40-60) | 150-200 | 250 | 30-60 | [161], [164], [12], [64] |
| Ethyl chloride |  | 200-300 |  |  | [87] |
| Ethylene glycol |  | 200-500 | 2000 | 11-19 (31^8 ; 278^) | [318], [319], [320], [321], [322], [323], [324], [325], [66], [326] |
| Ethylmorphine^48^ | 0.3-0.6^377^ |  | 0.3-2.9 | 2-3 | [47] |
| Etidocaine | 0.5-1.5 | 1.6-2 |  | 2-3 | [327] |
| Etidronat (Etidronic acid) |  |  |  | -^6^ | [49] |
| Etilefrine | appr. 0.06 |  |  | 2-3.5 |  |
| Etizolam^48^ | appr. 0.008-0.02 | 0.03^8^ |  | 7-15 | [47] |
| Etodolac | 10-20 (> 14^229^) |  |  | 6-8 | [328], [3], [15], [8] |
| Etomidate | 0.1-0.5 (-1) |  |  | 3.9±1.1 (2-11) | [3], [9] |
| Etoposide | 2-6 (peak 8-14) |  |  | 4-11 | [3], [11] |
| Etoricoxib | 1.3-3.6 |  |  | 20-36 | [47] |
| Etravirine (ETR) | 0.275 (0.081-2.98)^302^ |  |  | appr. 41 | [47], [110], [2] |
| Everolimus | 0.003-0.008 (-0.014)^275^ |  |  | 28±7 | [329], [330], [331], [332] |
| Ezetimibe | >0.015 |  |  | appr. 30 | [333], [334] |
| Famotidine | 0.02-0.2 | 0.42^8^ |  | 2-4.5 | [3], [335]. [336] |
| Fampridine | see 4-Aminopyridine |  |  |  |  |
| Felbamate | (30-) 50-110^164^ | 150-200 |  | 15-23 | [337], [4], [12], [338], [339] |
| Felbinac | appr. 0.4-1^326^ |  |  | 10-17 | [3], [340] |
| Felodipine | 0.001-0.012 | 0.01 |  | 22-27^88^ | [341], [84], [8] |
| Fenbufen | appr. -60 |  |  | 10-12 | [3] |
| Fendiline | 0.02-0.15 |  |  | appr. 20 | [47] |
| Fenfluramine | 0.04-0.3 | 0.5 – 0.7 | 6 | 18-25 | [47] |
| Fenitrothion |  |  | 1.1 | 33-64 | [47] |
| Fenofibrate | 5-30^241^ |  |  | 20-22 | [3], [342] |
| Fenoldopam | 0.003-0.06 |  |  | 0.1 | [15], [8] |
| Fenoprofen | (25-) 30-60 |  |  | 2-3 | [343] |
| Fenoterol | (0.001-) 0.01-0.04 |  |  | appr. 7 |  |
| Fentanyl | 0.003-0.3^4^ | -^339^ | 0.003-0.02^8; 103^ | 1-3.5 (transdermal patch: appr. 17) | [335], [47], [344], [345], [52], [346], [54], [347], [348], [349], [350], [351] |
| Fenthion |  |  | 4.9 (0.6-16.6^279^) | 12 | [47] ,[186] |
| Fexofenadine | appr. -0.3 ^191^ |  |  | 14-18 | [8], [352] |
| Finasteride | 0.008-0.01 |  |  | 5-7 | [353] |
| Flecainide^48^ | (0.2-) 0.4-0.8 | 1-2 | 2.6^8^; 13^8^ | 10-20 | [354], [70] |
| Fleroxacin | 1-4 |  |  | 8-13 |  |
| Flucloxacillin | 3-30 |  |  | 1-2 | [11], [58] |
| Fluconazole | appr. 1-5 (-15) | 20; 95^8^ |  | 22-31^83^ | [355],[356], [357], [358], [11] |
| Flucytosine | 35-70 (20-50) | 100 |  | 3-5 | [91] |
| Flumazenil^22^ | (0.01-) 0.02-0.1 | 0.5 |  | 1-2 |  |
| Flunarizine | 0.025-0.2 | 0.3 |  | -^6^ | [42] |
| Flunitrazepam^23^ | 0.005-0.015 | 0.05 |  | 10-20 (-30) | [359], [56], [59] |
| Fluoride | 0.095-0.190 (-0.285)^39^ | 0.5-2 | 3 | 2-9 | [47] ,[123], [360], [361], [130], [10], [58], [362] |
| 5-Fluorouracil | 0.05-0.3 | 0.4-0.6 |  | <0.5 | [3], [11], [58] |
| Fluoxetine | 0.12-0.5^130^ | 1 | 6^8^ | 2-6^6 ; 130^ | [263], [75], [202], [363], [4], [264], [364] |
| Flupenthixol | 0.001-0.01 | 0.015^311^ |  | 20-40 | [4] |
| Fluphenazine | 0.001-0.01 | 0.015^311^ |  | 10-18^45^ | [4] |
| Flupirtine | 0.5-1.5 | appr. 3-4 |  | 7-11 | [365] |
| Flurazepam^24^ | 0.02-0.1 | 0.2-0.5 | 0.8 ; 24^8^ | appr. 2^24^ |  |
| Flurbiprofen | 5-15 |  |  | 3-4 | [366] |
| Fluspirilen | 0.0001-0.0022 | 0.0044^311^ |  | 7-14^6^ | [4] |
| Flutamide^60^ | 0.4-1.5^60^ |  |  | 7-20^60^ | [43], [367] |
| Fluvastatin | 0.05-0.4 |  |  | 1-3 | [47] |
| Fluvoxamine | 0.06-0.23 | 0.5^311^-0.65; 1.97^8^ | 2.8^8^ | (8-) 15-22 (-28) | [47], [368], [202], [4], [369] |
| Fosamprenavir (FPV) | > 0.4^292^ |  |  | 7.7^319^ | [110], [2], [111] |
| Fosphenytoin^336^ | see Phenytoin | 30 |  | 0.13-0.25 | [12] |
| Frovatriptan | 0.004-0.008 |  |  | 20-30 | [47] |
| Furosemide (Frusemide) | 2-5 (-10) | 25-30 |  | 1-3 | [3], [58] |
| Fusidinic acid | 30-200 |  |  | 4-6 |  |
| Gabapentin | appr. 0.5-6 (-20-30)^185^ | 25^311^; 45^8^; 85^8^; 105^8^ |  | 5-9 | [47] ,[370], [371], [372], [373], [374], [375], [12], [376], [377], [378], [379], [380], [381], [382], [48] |
| Galant(h)amine | appr. 0.03-0.06 | 0.09^311^ |  | 6-8 | [383], [4], [384] |
| Gallopamil | 0.02-0.1 |  | 8^8^ | 3-8 | [385], [84] |
| Gamma-hydroxybutyricacid (gamma-hydroxybutyrate, gamma-butyrolactone, GHB, liquid ecstasy)^48^ | see 4-Hydroxybutyrate |  |  |  | [386], [387] |
| Ganciclovir | (0.29-0.51) 0.5-5^107^ | 3-5 |  | 2-4^83^ | [388], [38], [39] |
| Gemcitabine | 15-20 µmol/L^146^ | -^192^ |  | 0.05 (0.18-0.43) | [209] |
| Gemfibrozil | appr. -25 |  |  | 1.5 | [3] |
| Gentamicin | (2-) 4-10^233^ | 12 |  | 1.5-6 | [389], [67], [390], [20], [391], [392], [393], [394] |
| Glibenclamide (Glyburide) | 0.05-0.2 | 0.6 |  | 10 | [47] |
| Glicazide | 1-3.7 |  |  | 6-14 | [47] |
| Glimepiride | 0.09-0.5 |  |  | 4-15 | [47] |
| Glipizide | 0.1-1 (-1.5) | 2 |  | 3-7 | [66] |
| Glutethimide | 0.2-5 | 10-30 | 20-50 | 5-20 | [30] |
| Glyburide | see Glibenclamide |  |  |  |  |
| Glyceryl trinitrate (GTN) | see Nitroglycerin |  |  |  |  |
| Gold | 3-8 | 10-15 |  | 21-31^6^ (oral) | [47], [58], [48] |
| Granisetron | 0.009-0.017 (?) |  |  | 3-14 | [3], [15], [8] |
| Griseofulvin | 0.3-1.3 |  |  | 22 | [8] |
| Guaifenesin | 0.3-1.4 |  |  | appr. 1 | [3], [15], [8] |
| Guanethidine | 0.01 |  |  | 5-10^6^ | [3], [58] |
| Halazepam^15^ | see Nordazepam |  |  | 30-40 |  |
| Haloperidol | 0.005-0.017 (0.001-0.02) | 0.05-0.5 | 0.5; 0.18^8, 74^ | 10-35^153^ | [43], [395], [185], [396], [193], [397], [398], [240] |
| Halothan | 22-260 |  | 3.4^8^; 8.3^8^ | 43 | [87], [48] |
| Hematin | 50-100 |  |  |  |  |
| Hemin | see Hematin |  |  |  |  |
| Heptabarb(ital) | 0.5-4 | 8-15 | 20 | 6-11 |  |
| Heptaminol | appr. 0.2-1 (-1.5) |  |  | 2-3 |  |
| Heroin (Diacetylmorphine or Diamorphine, [DAM])^337^ | -^338^ | -^339^ |  | 2-5 min | [273], [274], [275], [276], [277], [278], [279], [280], [281] |
| Hexachlorobenzene (HCB) | -0.0001^332^ |  |  | appr. 2 years | [100] |
| ß-Hexachlorocyclohexane (ß-HCH, ß-lindane) | -0.0001^332^ |  |  | appr. 7 years | [100] |
| Hexachlorophene | 0.003-0.65 (-1) |  | 35 | 6-44 | [11], [58] |
| n-Hexane | -0.01 |  |  | 1.5-2 | [47] |
| Hexapropymate | 2-5 | 10-20 |  |  | [8], [11] |
| Hexobarbital | 1-5 | 10-20 | 50 | 4-6 |  |
| Hirudin-rec | -^171^ | -^171^ |  | (1-) 2.5-3 | [399], [43], [3] |
| Hydralazine^5^ | 0.05-0.5 (-1.5) |  |  | 2-6 | [84] |
| Hydrochlorothiazide | appr. 0.04-2 |  |  | 10-12 | [3], [42] |
| Hydrocodone | 0.01-0.05 | 0.1^339^ | 0.2 | appr. 4 | [8], [244] |
| Hydromorphone | appr. 0.005-0.015 | 0.1^339^ | 0.2 | 2-3 | [400], [9], [8], [270] |
| 4-Hydroxybutyrate (GHB)^48^ | appr. 50-120^4^ | 80 (abuse) | 250-280 (abuse) | 0.3-0.5 (-1) | [47], [3], [8], [244], [401], [402] |
| Hydroxychloroquine | -0.1 (-0.4) | 0.5-0.8 | 4 | dose-dependent^6^ | [3], [403], [13], [14] |
| 4-Hydroxy-3-methoxymethamphetamine (HMMA) | -^333^ |  |  | 11.5-13.5 | [404], [405], [406] |
| Hydroxyzine | 0.05-0.1 | 0.1 | 39^8^ | 7-20 | [56], [11], [58] |
| Ibandronat (Ibandronic acid) | -^321^ |  |  |  | [49] |
| Ibuprofen | 15-30 | 200 | 352^8; 346^ | 2-3 | [47], [407], [343], [293], [408] |
| Idebenone | 0.05-0.2 |  |  | 16-22 | [3], [409] |
| Iloperidone | 0.005-0.01 | 0.02^311^ |  | 18-33 | [4] |
| Iloprost | appr. 0.0001 |  |  | appr. 0.5 |  |
| Imatinib | 0.72^258^ |  |  | appr. 18 | [410], [411] |
| Imipenem | 0.5-5 (20-75) |  |  | 1 | [8], [412] |
| Imipramine^48, 125^ | 0.05-0.35 | 0.5-1 | 1.5-2 | 6-20^26^ | [308], [74], [309], [76], [77], [413], [78], [79], [80] |
| Indinavir (IDV) | > 0.1^260^ | appr. 0.5 |  | 1.5-2 | [211], [3], [110], [2], [414], [111] |
| Indomet(h)acin | 0.3-1 (-3) | 4-5 |  | 3-11 | [415] |
| Indoramin | appr. 0.025-0.1 |  |  | 12 (3.5-15) | [95] |
| Iproniazid | appr. -5 ? |  |  |  |  |
| Irbesartan | 1.9-3.3 |  |  | 11-15 | [47] |
| Iridium | -0.02 |  |  |  |  |
| Iron | 0.5-2 | 6 | 17 |  | [42], [58], [64] |
| Isoniazid (INH) | 5-10 | 20 | (30-) 100 | 1-3 | [3], [317], [8] |
| Isopropanol^361^ |  | 200-400 | 1000 | 2.5-3 | [47], [58] |
| Isosorbid mononitrate (ISMN) | 0.1-1 |  |  | 2-5 | [95] |
| Isotretinoin | appr. 0.001-0.002 (topical)^224^ |  |  | 10-20 | [3], [416], [417] |
| Isoxicam | 5-15 |  |  | 20-50 |  |
| Isradipine | 0.0005-0.002 (-0.01) | 0.01 | 0.26^8, 259^ | 5-10 | [418], [3], [84], [419], [420] |
| Itraconazole | appr. 0.4-2^111^ |  |  | 24-36 | [421], [422], [423], [424] |
| Ivermectin | appr. 0.05^51^ |  |  | 16-28 | [3], [425] |
| Kanamycin | 1-4 (10-25) | 25-30 |  | 0.5-3 |  |
| Kavain | appr. 0.05 |  |  | 2.8-6.7 | [47] |
| Ketamine | 1-6 | 7 (abuse) | 3.8^8^; 6.9^8^ | 1-3 (-4) | [47], [426], [56], [427], [428] |
| Ketanserin | 0.05-0.5 |  |  | 10-22 |  |
| Ketazolam^15^ | 0.001-0.02 |  |  | 1-3 | [59] |
| Ketobemidone | 0.01-0.05 |  | 0.2-3.2 | 1.8-4.2 | [47], [426], [3], [428] |
| Ketoconazole | 1-3 (-6) |  |  | 6-10 | [8] |
| Ketoprofen | 1-6 (-20) |  | 1100^8^ | 1,1-2 (-4.2) | [429], [47], [430], [343], [431], [432], [433] |
| Ketorolac | 0.5-3 | 5 |  | 4-10 | [3], [8] |
| Ketotifen | 0.001-0.004 | 0.02 | 1.2^8^ | 21 | [57] |
| Labetalol | 0.03-0.18 (-0.65)^50^ | 1 |  | 3-10 | [3], [95], [7] |
| Lacidipine | 0.003-0.006 |  |  | 12-19 | [8] |
| Lacosamide | 1-10 | 20^311^ |  | 13 | [4] |
| Lamivudine | -^230^ |  |  | 5-7 | [123], [434],[2] |
| Lamotrigine | (1-5) 3-14 | 20-30 | 35.7^8; 347^; 50^8^ | 23-37^109^ | [47], [374], [435], [436], [381], [437] |
| Lead | -0.09^324^ | 0.4-0.6 | 3 | -^180^ | [47], [64], [123], [438], [439], [100] |
| Leflunomide^255^ | 8.8 ± 2.9, 18 ± 9.6, 63 ± 36^256^ |  |  | 11 (4-28)^6^ | [440], [123], [441], [442] |
| Levacetylmethadol (LAAM) | appr. 0.02-0.06 |  |  | 35-60 | [8] |
| Levamisole | appr. 0.1-0.7 |  |  | 3-6 | [8] |
| Levetiracetam | (3-) 10-40 | 400^8; 264^ |  | 4-10 | [8], [443] |
| Levocabastine | < 0.001-0.01^147^ |  |  | 33-40 | [444] |
| Levocetiricine^378^ | 0.3-0.5 |  |  | 6-10 | [47] |
| Levodopa (L-Dopa) | 0.3-2 | 5^311^ | 650^8^ | 1-3^215^ | [445], [446], [447], [448], [4], [449] |
| Levomepromazine^27^ | 0.005-0.025 (-0.2) | 0.4 | 0.5 | 15-30^28^ |  |
| Levomethadone^352^ | 0.04-0.4 | (0.4-) 1^339^ | 0.1-0.2^362^ | 10-40 | [123], [4], [450] |
| Levorphanol | 0.007-0.02 | 0.1 | 2.7^8^ | 11-30 | [8] |
| Levothyroxine | 0.045-0.14^47^ |  |  | 6-8^6^ | [451] |
| Lidocaine (Lignocaine) | (1-) 1.5-5^113^ | 6-7 | 10 | 1-4^113^ | [452], [453], [391], [70], [327], [454] |
| Linezolide | appr. 0.5-4 |  |  | appr. 5 | [8] |
| Lisinopril | (0.005-) 0.02-0.07 | 0.5 |  | 12 | [84] |
| Lithium | 4-8^79^ | 13 | 14 | 8-50^28^ | [455], [456], [457], [80], [264], [458] |
| Lofepramine | 0.003-0.01 |  |  | 10-20 |  |
| Loperamide | -^84^ |  |  | 7-15 | [459] |
| Lopinavir (LPV) | > 1.0^294^ |  |  | 5-6 | [110], [2], [111] |
| Loprazolam | 0.003-0.01 |  |  | 11-20 | [59] |
| Loratadine | 0.001-0.02^138^ |  |  | 8-14 | [460], [461] |
| Lorazepam | (0.02-) 0.08-0.25 | 0.3-0.5 |  | 10-40 | [462], [59], [463], [224] |
| Lorcainide | 0.1-0.4 (-0.9) |  |  | 5-10 | [8], [464], [465], [466] |
| Lormetazepam | 0.002-0.01 (-0.025) | 0.1 |  | 10-15 | [4], [59] |
| Lornoxicam | 0.1-0.8 |  |  | 2-6 | [8] |
| Losartan | < 0.2 (-0.65)^227^ |  |  | 1.5-2 | [3], [8] |
| Loxapine | 0.01-0.03 (-0.1) | 1 | 7.7 | 4 (1-14) | [8], [11] |
| Lysergide (lysergic acid diethyl amide, LSD) | 0.0005-0.005 | 0.001 | 0.002-0.005 | appr. 2-5 | [467], [15], [8], [11] |
| Magnesium | 55-75^121^ | 120-140 | 150-180 | 2.1-2.9 | [47], [3], [468] |
| Malathione |  | 0.35^8^; 0.5 | 1.8^8^; 175^8^; 517^8^ | 3-6 | [47] |
| Manganese | 0.0005-0.0015 |  |  | 12-36^6^ | [47] |
| Maprotiline | 0.075-0.13 | (0.22-) 0.5-1 | 1-5 | 20-60 | [4] |
| Maraviroc (MVC) | > 0.05^300^ |  |  |  | [110] |
| MCPA | see 2-Methyl-4-chlorophenoxyacetic acid |  |  |  |  |
| MCPP | see 2-Methyl-4-chlorophenoxypropionic acid |  |  |  |  |
| MDA | see Methylenedioxyamphetamine |  |  |  |  |
| MDEA | see Methylenedioxyethylamphetamine |  |  |  |  |
| MDMA | see Methylenedioxymethylamphetamine |  |  |  |  |
| Mebendazole | ≥ 0.1^67^ | appr. 0.6 |  | 2.8-9 | [43] |
| Medazepam^30^ | 0.1-0.5 (-1) | 0.6 |  | 2-5 | [59], [8] |
| Mefenamic acid | 2-10 (-20) | 25 |  | 2-4 | [9], [13], [14] |
| Mefloquine | 0.4-1^108^ | 1.5-2^8^ |  | appr. 21^6^ | [469], [470] |
| Melatonin | 0.0005-0.1^379^ |  |  | 40-50 min | [47] |
| Melitracen | 0.01-0.1 |  |  | 12-23 | [8] |
| Meloxicam | 0.4-2 |  |  | 17-22 | [471], [15], [8] |
| Melperone | 0.03-0.1^219^ | 0.2^311^ | 17.1^8^ | 4-8 | [4], [472], [15], [8], [473] |
| Melphalan | -1.5 |  |  | 1.5-2 | [8] |
| Memantine | 0.09-0.15 | 0.3^311^ |  | 60-100 | [4] |
| Meperidine | see Pethidine |  |  |  |  |
| Mephenesin | 3-10 (?) |  |  | appr. 2-4 | [15], [8] |
| Mepindolol | 0.007-0.07 |  |  | 3-6 | [7] |
| Mepivacaine | appr. 0.4 (-4) | 5-6 (-10) | 50 | 1-3 | [15] |
| Meprobamate | 5-10 | 10-25 | 30 | 6-17 | [56] |
| Meptazinol | 0.025-0.25 |  |  | 2-3 | [8] |
| Mercaptopurin^364^ | 0.03-0.08 | 1-2 |  |  | [8] |
| Mercury | appr. 0.0015-0.002 (< 0.005)^178^ | 0.05-0.2 | 0.5 | appr. 3^6^ | [123], [64], [474], [66], [475], [100], [476], [477], [478] |
| Mescaline | 1.5-3.8 |  |  | 6 | [47] |
| (Mesalazine (Mesalamine) | appr. -1^119^ |  |  | 0.5-2.4^120^ | [479] |
| Mesoridazine | 0.15-1 | 3-5 | 3^8^; 4^8^; 16^8^ | 20 | [15] |
| Mesuximide | see Methsuximide |  |  |  |  |
| Metaclazepam | 0.05-0.2 |  |  | 7-23 | [59] |
| Metamizole (Dipyrone)^5^ | 10^32^ | 20^32^ |  | 6-8 | [15] |
| Metformin | 0.1-1 (0.6-1.3) | 5-10 | 64^8^; 85^8^; 91^8^; 166^8^ | 2-4 (-10) | [480], [481] |
| Methadone | (0.05-) 0.1-0.5 (-0.75)^135^ | 0.2^339^ | 0.4^362^ | 23-25 (13-55) | [482], [483], [484], [485], [486], [487], [488], [489] |
| Methamphetamine (Methylamphetamine) | -0.1 | 0.15 | 1-18; 40^8^ | 6-15^344^ | [47], [8] |
| Methanol | appr. -2 | 200 | 900 | 10-12 (-24)^325^ | [47], [325], [15], [8], [58], [490] |
| Methapyrilene | appr. 0.1 | 4 |  |  |  |
| Methaqualone | 1-3 | 3-5 | 5-10 | 10-40 | [8] |
| Methemoglobin (Met-Hb) | -^199^ | 25-30 % | 60-70 % |  | [3], [10], [11] |
| Methimazole | 0.5-2.5 |  |  | 2-28 | [467], [15], [8] |
| Methocarbamol | 25-40 (-50) | 250 |  | 0.9-2 | [3], [11],[58] |
| Methohexital | 1-6 (-11)^55^ | 2-20 |  | 1-3 | [8] |
| Methomyl |  | 0.63^8^; 1.6^8^ | 1.6^8^; 28 (8-57) |  | [47] |
| Methotrexate | 0.04-? | 0.4 |  | 5-9 (low dose); 16-29 (high dose) | [47] |
| Methotrimeprazine | see Levomepromazine |  |  |  |  |
| Methoxsalen (8-Methoxypsoralene) | 0.025-0.1 (-0.2) | 1 |  |  | [3], [10] |
| Methsuximide (Mesuximide) | 10-40^223^ | 40-50 |  | 20-40 (-45) | [3], [491], [8], [11], [58] |
| 2-Methyl-4-chlorophenoxyacetic acid (MCPA) | - | appr. 100 | appr. 180 | -^187^ | [3], [492] |
| 2-Methyl-4-chlorophenoxypropionic acid (MCPP) | - | appr. 100 | 669^8^; 715^8,181^ | 17^183^ | [3] |
| Methyldopa | 1-5 | 9^8^ |  | 1.5-3 | [9], [13], [14] |
| 3,4-Methylenedioxyamphetamine (MDA) | -0.4 | 1.5 | 1.8-2; 26^8^ | 10.5-12.5 | [15], [8], [58], [467], [404], [405], [406], [493], [494] |
| 3,4-Methylenedioxyethylamphetamine (MDEA, MDE; Eve) | -0.2 |  | 1; 4.2^8^; 12^8^ | 4-8^334^ | [47], [493], [495], [496], [497] |
| 3,4-Methylenedioxymethylamphetamine (MDMA; Ecstasy, XTC; Adam) | 0.1-0.35^236^ | 0.35-0.5 | 0.4-0.8 | 7-8 (-10) | [498], [467], [15], [8], [58], [404], [405], [499], [406], [493], [494] |
| Methylphenidate | 0.01-0.06 | 0.1-0.5; 1^8^ | 2.3 | 2-7 | [270] |
| Methylphenobarbital | see Phenobarbital |  |  |  | [12] |
| 4-Methylthioamphetamine (4-MTA, p-MTA) |  |  | 2^8^; 4.2^8^; 7.4^8^ |  | [500], [501], [502], [503], [504] |
| Methyprylon(e) | < 10-20 | 25-75 | 50 (-100) | 3-6, 9-11 | [8] |
| Metiamide | 0.01-0.06 |  |  |  | [58] |
| Metildigoxin^3^ | 0.0005-0.0008 | 0.0025-0.003 | 0.005 | 40-70 | [18], [19], [20], [21], [22], [23], [24], [25], [26], [27] |
| Metipranolol^33^ | 0.02-0.08 |  |  | 2-3.5 |  |
| Metoclopramide | 0.05-0.15 | 0.2 | 4.4^8^ | 3-6 | [66] |
| Metocurine | appr. -0.4 |  |  |  |  |
| Metoprolol^48^ | 0.035-0.5 | 0.65^8^; 12-18 | 4.7^8^; 12^8^; 18^8^; 63^8^ | 2.5-7.5 | [47], [3], [7] |
| Metrifonate | appr. 1.4-3.6 |  |  | 2-5 | [8] |
| Metronidazole | 3-10 (-20) | 200^8^ |  | 6-10 (-14) | [8] |
| Mexiletine | 0.7-2 | 2 | 35^8^ | 5-26 | [70], [505] |
| Mianserin | 0.015-0.07 | 0.14^311^; 0.25-0.5 |  | 14-33 | [4], [223] |
| Mibefradil | appr. 0.2-0.3 |  |  | 17-25 | [8] |
| Miconazole | appr. 1 |  |  | 24 | [8] |
| Midazolam | 0.04-0.1 (-0.25)^134^ | 1-1.5 |  | 1.5-3^46^ | [506], [507], [508], [61], [224] |
| Mifepristone | 216 |  |  | 24-48 (20-54) | [509] |
| Milnacipran | 0.05-0.11 | 0.22^311^ |  | 5-8 | [4] |
| Milrinone | 0.15-0.25 | 0.3 |  | 1-2 | [3], [95], [42] |
| Minaprine | appr. -0.1 |  |  |  |  |
| Minoxidil | appr. 0.02-0.2^149^ | 1.4^8^; 3.1^8^ | 2.7^8^ | 1.3-4.2 | [47], [3], [84] |
| Mirtazapine | 0.03-0.08 (-0.3) | 1-2; 2.3^8^ |  | 20-40^188^ | [3], [4], [510] |
| Misoprostol | -^286^ |  |  | 0.5±0.4 | [3], [511], [512] |
| Mitotane | 14-20 | 20 |  | 18-159^6^ | [513] |
| Mizolastine | appr. 0.2-0.8 |  |  | 8-17 | [3], [8] |
| Moclobemide^141^ | 0.3-1.0 (-3) | 2^311^; 11^162^; 25-60 |  | 1-7 | [514], [515], [516], [517], [518], [519] |
| Modafinil | 1-1.7 (-3)^257^ | 3.4^311^ |  | 10-15 | [89], [4], [8], [520] |
| Moexiprilat | 0.005-0.04 |  |  | 2-10 | [8] |
| Molindone | 0.04-0.5 | 0.15^8^ | 6^8^; 9.3^8^ | 1.2-2.8 | [47] |
| Molsidomine | 0.002-0.01 |  |  | 1-2.5 | [95] |
| Molybdenum | -0.005 |  |  |  |  |
| Montelukast | appr. 0.05-0.3 |  |  | 3-6 | [3], [15], [8], [521] |
| Moricicine | 0.12-1.27 |  |  | (3-) 6-13 | [467], [15], [8] |
| Morphine^288^ | 0.01-0.1 | 0.1^339^ | 0.1-4 | 1-4 | [522], [241], [523], [524], [87], [525] |
| Moxonidine | 0.001-0.002 (-0.004) |  |  | 2-4 | [3], [95], [8] |
| Muromonab-CD3 (OKT 3) | appr. 0.7-1.3 |  |  | appr. 18 |  |
| Mycophenolate mofetil | -^211^ |  |  | 16-18^212^ | [526], [527], [528], [529] |
| Nabumetone | -^206^ |  |  | -^206^ | [3], [530] |
| Nadolol | 0.01-0.25 |  | 1.3^8^ | (14-) 20-24 | [8], [7] |
| Naftidrofuryl (Nafronyl) | <0.5 |  |  | 1-2 |  |
| Nalbuphine | 0.02-0.2 |  |  | 2.5-7 | [8] |
| Nalidixic acid | 10-30 | 40-50 |  | 1-2 (-7) | [3], [9], [15], [8] |
| Nalmefene | -0.1 |  |  | 8.5-11 | [3] |
| Naloxone | 0.01-0.03 |  |  | 1-2 | [8] |
| Naltrexone | 0.003-0.05^99^ |  |  | 4-10 |  |
| Naphyrone (Naphthylpyrovalerone) |  | 0.03^334^ |  |  | [531] |
| Naproxen | 20-50 (-100) | 200-400; 414^8^ |  | 10-20 | [532], [343], [533], [293] |
| Naratriptan | appr. 0.01-0.05 |  |  | 5-6 | [3], [15], [8] |
| Nebivolol | < 0.02 (-0.2) | 0.48^8^ |  | 10 (8-27) | [3], [8], [534], [535], [536] |
| Nedocromil | < 0.025 |  |  | 1.5-3.3 | [3] |
| Nefazodone | appr. 0.01-0.3 (?)^220^ | 5.5^8, 221^ |  | 2-7^222^ | [8], [3], [537], [538], [539], [540], [541], [542] |
| Nefopam | 0.01-0.1 | 4^8^ | 12^8^ | 3-8 | [8] |
| Nelfinavir (NFV) | > 0.8^295^ |  |  | 3.5-5 | [110], [87], [2], [111] |
| Neostigmine | appr. 0.001-0.01^127^ |  |  | 0.4-1.3 | [316], [543] |
| Netilmicin | 1-12 |  |  | 2-3^80^ | [8] |
| Nevirapine (NVP) | > 3.0^298^ |  |  | 25-30 | [110], [2], [111] |
| Nicardipine | 0.07-0.1 |  |  | 7-12 | [84] |
| Nickel | -0.003^330^ | 0.005 |  |  | [58], [100] |
| Nicotine^123^ | 0.005-0.02 (-0.03) | 0.4 (-1) | 5; 13.6^8^ | 1-4^124^ | [3], [544], [42], [545] |
| Nicotinic acid | 4-18 |  |  | 0.3-1 | [8] |
| Nifedipine | 0.025-0.15 | appr. 0.15-0.2 | 0.15^8^; 1.2^8^; 5.4^8^ | 2-5 | [47], [84] |
| Niflumic acid | 2-35 |  |  | 2-3 | [15] |
| Nilvadipine | < 0.01 |  |  | 11-20 | [84] |
| Nimesulide^235^ | 0.1-6.5 |  |  | 2-7 (11-20) | [546], [3], [15], [8] |
| Nimodipine | 0.01-0.05 |  |  | 1-2 (8-9) | [84] |
| Nimustine | 0.0002-0.0005 |  |  |  | [58] |
| Nisoldipine | 0.0003-0.001 |  |  | 7-12 | [3], [84] |
| Nitrazepam | 0.03-0.1 | 0.2-3 | 5 | 20-30 | [8], [59] |
| Nitrendipine | 0.01-0.05 |  |  | 8-12 | [84] |
| p-Nitroaniline |  |  | 4.2^8^ |  | [547] |
| Nitrofurantoin | (0.5-) 1-3 (-5) | 3-4 |  | 0.7-1.5 | [8] |
| Nitroglycerin (Glyceryl trinitrate) | appr. -0.015 |  |  | 20-30 min | [548] |
| Nitroprusside | see Thiocyanate | see also Cyanide |  |  |  |
| Nizatidine | 0.05-0.5 (-1.0) |  |  | 0.7-2.1 | [8] |
| Nomifensine | 0.01-0.1 | 8 | 17^8^ | 2-5 | [87], [8] |
| Nordazepam | (0.02^273^-) 0.2 -0.8 | 1.5-2 |  | 40-80 | [284], [285], [467], [8], [15] |
| Nordiazepam | see Nordazepam |  |  |  |  |
| Norephedrine | see Phenylpropanolamine |  |  |  |  |
| Norfenefrine | -0.4 |  |  | 2-3 | [8] |
| Norfloxacin | 0.5-5 |  |  | 3-4 | [3], [58] |
| Normesuximide^31^ | 10-30 | 40 |  | 38 |  |
| Nortriptyline^48^ | 0.02-0.2 (0.05-0.15) | 0.3^311^; 0.5 | 1-3 | 18-56^68^ | [76], [80], [81], [82], [223] |
| Noscapine | 0.02-0.4 |  |  | 1.5-4 | [47] |
| Obidoxime | 1-10 (appr. 10-15 µmol/L) |  |  |  | [549], [58] |
| Ofloxacin | appr. 2.5-5.5 | (30-) 40^8^ |  | (3-) 5-8 | [3], [550] |
| OKT 3 | see Muromonab-CD3 |  |  |  |  |
| Olanzapine | 0.02-0.08 | 0.15-0.2 | 0.25^8^; 1^8^; 2.5^8^; 4.9^8^ | 30-60 | [47], [551], [101], [552], [553], [4], [72], [554], [15], [555], [58] |
| Olpadronat (Olpadronic acid) |  |  |  | -^6^ | [49] |
| Omeprazole^48^ | 0.05-4^98^ |  |  | 0.5-1 (-1.5) |  |
| Ondansetron | 0.03-0.3 |  |  | 3-5.5 | [8], [66] |
| Opipramol | 0.05-0.5 | 1^311^; 2-3 | 7-10 | 6-12^262^ | [43], [556], [4] |
| Orphenadrine | 0.1-0.2 (-0.6) | 1.7 | 3.6^8^; 5-7 | (10-) 14-18 | [296], [56], [9], [557] |
| Oxatomide | 0.02-0.1 |  |  | 14-30 | [3] |
| Oxazepam | 0.2-1.5 | 2 | 3-5 | 6-20 | [283], [3], [59], [13] |
| Oxazolam | see Nordazepam |  |  |  |  |
| Oxcarbazepine | 10-35^172^ | 45 |  | 1-2.5 (-5)^172^ | [3], [558], [12], [559], [560], [381] |
| Oxibutynin | 0.001-0.02 |  |  |  | [8] |
| N-Desethyloxibutynin | 0.01-0.08 |  |  |  | [8] |
| Oxpentifylline | see Pentoxyfylline |  |  |  |  |
| Oxprenolol | 0.05-0.3 (-1.0) | 2-3 | 10 | 1-4 | [7], [8] |
| Oxycodone^48^ | 0.005-0.1 | 0.2^339^ | 0.6; 5^8^ | 2-5^53^ | [9], [42], [11], [58], [244] |
| Oxyfedrine | appr. 0.06 |  |  | 4.2 | [95] |
| Oxyphenbutazone | 25-100 | 200 |  | 48-72 | [47], [8] |
| Oxypurinol^61^ | 5-15 | 20 |  | 18-30 | [11], [58] |
| Oxytocin | appr. -0.0002 |  |  | 3-5 min | [3] |
| Paclitaxel | 0.1-1^122^ |  |  | 4-8 (-20) | [8] |
| Paliperidone | 0.02-0.06 | 0.12^311^ |  | 23 | [4] |
| Pamidronate (Pamidronic acid) | < 0.02^323^ |  |  | -^6^ | [49], [561] |
| Pancuronium | 0.1-0.6 | 0.4^8; 198^ | 1.6 | 1.5-2.5 | [562] |
| Pantoprazole | appr. -4.6^98^ |  |  | 1-2 | [8], [3] |
| Papaverine | 0.2--2 |  |  | 1-2 (6-7) | [57] |
| Paracetamol | (5-)10-25 | 100-150 | 200-300 | 2-4 | [563], [564], [565], [566], [567], [568], [569], [243], [570], [571] |
| Paraldehyde | 10-100 | 200 | 400-500 | 4-10 | [87], [48] |
| Paraoxon | - | 0.005 |  |  | [11] |
| Paraquat | - | 0.05 | 0.12^8; 348^; 1-2^201^ | 8-12^349^ | [123], [572], [11], [573], [574], [575], [576], [577], [578], [579], [580] |
| Parathion | - | 0.01-0.05 | 0.05-0.08 |  | [3], [10], [58] |
| Paroxetine^48^ | < 0.01-0.05 (-0.12) | 0.35-0.4 | 3.7^8^; 4^8 ;306^ | 16-24^93^ | [263], [368], [202], [581], [582], [583] |
| Pefloxacin | 1-10 (3-6) | 25 |  | 8-15 | [8] |
| Pemoline | appr. 1-7 |  |  | 7-13 | [8] |
| Penbutolol | 0.01-0.3 (-1.0) |  |  | 20-26 | [7] |
| Penfluridol | 0.004-0.025 |  |  | 70 | [3], [11] |
| (D-)Penicillamine | 1.7-5.6 (-11) |  |  | 1-3 | [8] |
| Pentachlorophenol | -0.2 | 30 | 45 |  | [11] |
| Pentamidine | 0.3-0.5 | appr. 0.8 |  | 6-9 | [3], [15], [8] |
| Pentazocine | 0.01-0.2 | 1-2 | 3 | 2-5 | [8] |
| Pentobarbital | 1-5 (-10) | 10-19 | 15-25 | 20-40 | [87], [48], [8] |
| Pentoxifylline^72^ | appr. 0.5-2 |  |  | 0.5-2 (4-6) | [8] |
| Pentoxyverine | -0.18 |  |  | 2-3 | [8] |
| Perazine | 0.02-0.35 (-0.44) | 0.5 (6.1^8^) |  | 8-16 (-35) | [584], [8] |
| Perhexiline | 0.11-0.6^309^ | 0.6-1.2 |  | (7-) 12-18 (-23) | [585], [586],  [3] |
| Perici(y)azine | 0.005-0.03 | 0.1 |  |  | [9] |
| Perindopril | 0.08-0.15 |  |  | 0.8-1.5^265^ | [15], [8], [3] |
| Perphenazine | 0.001-0.02 (0.0006-0.0024)^161^ | 0.005^311^; 0.05 |  | 8-12 (-21) | [587] |
| Pethidine | 0.1-0.8^115^ | 1-2 | 2 (-3) | 3-6 (-10) | [588], [589], [590], [591], [592] |
| Phenacetin^62^ | 5-10 (-20) | 50 |  | appr. 1 | [9], [13] |
| Phenazepam | 0.02-0.04 |  |  | 60 | [47] |
| Phenazone (Antipyrine) | 5-25 | 50-100 |  | 10-12 | [8] |
| Phencyclidine (PCP) | 0.01-0.2 | 0.007-0.24 (-0.8) | (0.3-) 1-5 | 1-12 (-50) | [467], [15], [8], [11], [58] |
| Phendimetrazine | 0.02-0.1 |  | 0.3-0.7 | 2-4 | [8] |
| Phenelzine | 0.001-0.002 (-0.04) | 0.5 | 1.5 | 6-8 | [3], [11], [58] |
| Pheneturide |  | 5-20 |  | 30-90 | [12] |
| Phenformin | 0.03-0.1 | 0.6 | 3 | 4-13 | [15], [8], [3] |
| Pheniramine | 0.01-0.27 |  | appr. 2 | 16-19 | [15], [8], [3] |
| Phenmetrazine | 0.02-0.25 | 0.5 | 4 | appr. 8 | [8] |
| Phenobarbital | 10-30 (15-40) | 30-40 | 50-60 | 60-130 | [161], [164], [415] |
| Phenol |  | 50 | 90 |  | [11] |
| Phenprocoumon | 0.16-3.6 (1-5) | 5 |  | 100-160^35^ | [11], [58] |
| Phensuximide | 4-10 (-20) | 80 |  | 4-12 | [8], [11], [58] |
| Phentermine | 0.03-0.1 | 0.9 | 1 | appr. 20 | [15], [8], [11], [58] |
| Phenylbutazone^36^ | 50-100 | 120-200 | 400 | 30-175^37^ | [9], [13] |
| Phenylephrine | 0.04-0.1 |  |  | 2-3 | [8] |
| Phenylpropanolamine (Norephedrine) | 0.1-0.5 | 2 | 48 | 3-7 | [3], [9] |
| Phenytoin | 5-15 (10-20)^81^ | 20-25 | 43^8^ ; 50 | 10-60^37^ | [164], [593], [594], [595], [596],[597], [598], [599], [600] |
| Pholcodine | appr. 0.07-0.2 |  |  | 35-75 | [47] |
| Physostigmine | < 0.001-0.005 |  |  | 0.4-1 | [316], [601], [303] |
| Pimozide | 0.004-0.01 (-0.02) | 0.02^311^ |  | 24-55 | [4] |
| Pinazepam^15^ | 0.01-0.05 |  |  | 16 | [59] |
| Pindolol | 0.02-0.15 | 0.7-1.5 |  | 2-5 | [7] |
| Pipamperone | 0.1-0.4 | 0.5-0.6 |  | 17-22 | [4] |
| Piperacilline | 1-5 (20-70) |  |  | 1-2 | [15], [8], [11], [58] |
| Piperazine | 0.02-0.1 | 0.5 |  |  | [42], [66], [58] |
| Pipotiazine | 0.001-0.06 | 0.1 |  | 8-11 | [3], [9], [8], [11], [58] |
| Piracetam | appr. 20-50 |  |  | 4.5-7 | [8] |
| Pirenzepine | 0.03-0.45 |  |  | 8-20 | [8] |
| Piritramide | 0.0035- 0.014^128^ | -^128^ |  | 4-10 | [602], [8] |
| Pirmenol | 1-4 |  |  | 6-18 | [3], [8], [11] |
| Piroxicam | 2-6 | 14^8^ |  | 30-70 | [8] |
| Pizotifen | 0.007-0.009 |  |  | 26 |  |
| Posaconazol | > 0.7 (for invasive aspergillosis) |  |  | 20-66 | [47], [603], [604], [605] |
| Practolol | 1.5-5 |  |  | appr. 6-8 | [8] |
| Prajmalium^48^ | 0.06-0.44 |  |  | 5-7 | [8] |
| Pramipexole | appr. 0.0002-0.007 | 0.015^311^ |  | 8-14 | [4], [467], [15], [8] |
| Pranlukast | appr. 0.2-1.2 |  |  | appr. 2-9 | [15], [8], [3] |
| Prazepam^15^ | 0.2-0.7 | 1 |  | 1-3 | [8], [59] |
| Praziquantel | appr. 0.2 |  |  | 1-2.5 | [8] |
| Prazosin | 0.001-0.02 | 0.9 |  | 2.9±0.8 | [95], [42] |
| Prednisolone | 0.5-1 |  |  | 2-6 | [8] |
| Pregabalin | 2-5 (-8) | 10^311^; 13^8^; 60^8^; 67^8^ |  | appr. 6 | [373], [4], [606], [607] |
| Prilocaine | 0.5-1.5 (-2)^126^ | 5-6 | appr. 20 | 1-2 | [608], [327] |
| Primaquine | appr. 0.1-0.2 |  |  | 4-7 | [8] |
| Primidone^63^ | 4-12 (8-15) | 20-50 | 65 | 4-12, 9-22 | [161], [164] |
| Probenecid | 100-200 (20-150) |  |  | 3-17^37^ | [8] |
| Procaine | 0.2-2.5 (-15) | 15-20 | 20 | -0.5 | [15], [11], [58] |
| Procainamide^5^ | (2.5-) 4-10 (-12) | 10-15 | 20 | 2-5 (-8) | [70], [609], [8] |
| N-Acetylprocainamide^38^ | (5-) 10-35 (-40) |  |  | 3-7 | [8] |
| Prochlorperazine | 0.01-0.05 | 0.2-0.3 | 5 | 7-9 (-18) | [3], [9], [58] |
| Procyclidine | 0.08-0.63 | 1-2 | 7.8^8^ | 7-16 | [15], [8], [11] |
| Proguanil^48^ | appr. 0.04-0.15^114^ |  |  | 13-24^114^ | [610], [58] |
| Promazine | 0.01-0.05 (-0.4) | 1 | 5 | 5-41 (8±7) | [8] |
| Promethazine | 0.05-0.2 (-0.4) | 1-2 | 2.4^8^; 1.8-5.4^250^ | 8-15 (-20) | [56], [611], [612] |
| Propafenone^48^ | (0.04-) 0.3-2 | (1.1-) 2-3 | 7.7^8^; 9^8^ | 5-8, 2-32^48^ | [613], [70], [42] |
| Propallylonal | 0.3-10 | appr. 10 |  | appr. 3 | [8] |
| 2-Propanol | see Isopropanol |  |  |  | [64] |
| Propantheline | appr. -0.02 |  |  | 1-3 | [3] |
| Propiomazine | < 0.3 (?) |  |  | 8-10 | [56], [614] |
| Propofol^48^ | appr. 2-8 |  |  | 3-8^91^ | [244], [615], [52], [616] |
| Propoxyphene | see Dextropropoxyphen |  |  |  |  |
| Propoxur |  |  | 0.3^8^ |  | [87] |
| Propranolol | 0.02-0.3 | (0.5-) 1-3 | 4-10 | 2-6 | [7], [112] |
| Propylene glycol | 0.05-0.5 | 1000; 4700^8^ |  | 2-5 | [47], [11], [58], [617] |
| Propylhexedrine | 0.01 | 0.5 | 2-3 |  | [58] |
| Propyphenazone | 3-12 |  |  | 1-1.5 (-3) | [9], [13], [14] |
| Prothipendyl | 0.005-0.01 | 0.02^311^; 0.1-0.5 |  | 2-3 | [4] |
| Protriptyline | 0.05-0.3 | 0.5 | 1; 20.7^8^ | 50-200 | [87], [58] |
| Pseudoephedrine | (0.05-) 0.5-0.8 |  | 19-20^280^ | 9-16 | [618], [266], [267], [269], [15], [11], [58], [619] |
| Psilocin^380^ | appr. 0.008 | 0.018^8^ |  | 1.8-4.5 | [47] |
| Psilocybin | see Psilocin |  |  |  |  |
| Pyrazinamide | 30-75 |  |  | 9-10(-25) | [620], [3], [9] |
| Pyridostigmine | < 0.05-0.2 |  |  | 1-2.5 | [316], [621], [622] |
| Pyridoxine | 0.003-0.018 |  |  | 3-6 | [66] |
| Pyrilamine |  |  | 11^8^ |  | [87] |
| Pyrimethamine | appr. -1.5 |  |  | 80-96 | [3] |
| Pyrithyldione | 1-10 |  |  | 11-20 | [9], [13], [14] |
| Quazepam | 0.01-0.05 (-0.15)^131^ |  |  | 39 (25-41) | [59], [8], [11], [58] |
| Quetiapine | 0.1-0.5^239^ | 1^311^; 1.8^8^ | 1.9^8^; 5.1^8^; 12.7^8^ | appr. 5-7^240^ | [623], [624], [625], [626], [87], [627], [628] |
| Quinidine^48^ | 1-5 | 6-10 | 10-15 | 4-12 | [70], [23] |
| Quinine | 1-7 | 10 |  | 4-15 | [629], [630] |
| Rabeprazole | appr. -0.6 |  |  | 1-2 | [15], [8] |
| Ramipril | appr. 0.001-0.04^228^ |  |  | 1-5 | [3], [8] |
| Ranitidine | 0.05-1 |  |  | 2-4 | [631] |
| Raltegravir (RAL) | 0.072 (0.029-0.118)^303^ |  |  |  | [110] |
| Reboxetine | 0.06-0.35 ^281^ | 0.7^311^ |  | 12-14 (-30) | [632], [3], [633], [4] |
| Recainam | 1.3-5.7 |  |  | 5-7 | [634] |
| Remacemide | appr. 0.1-1 |  |  | 4 | [8] |
| Remifentanil | -0.02 |  |  | 0.15 | [8] |
| Remoxipride | 2.15±0.59^132^ |  | 41-150 | 5-10 | [56], [635], [636] |
| Retinol (Vitamin A) | 0.2-0.8 (0.7-2.8 µmol/L) |  |  |  | [66], [637] |
| Ricin |  | 0.0005 |  |  | [638] |
| Rifabutin | 0.05-0.15 |  |  | 24-58 | [47], [3] |
| Rifampicin (Rifampin) | 0.1-10^101^ | 204^8^ | 55^8^ | 1-6^37^ | [47], [620], [317], [639] |
| Rifapentine |  |  |  | 13.2 | [620] |
| Riluzole | appr. 0.05-0.5 (-1.5) |  |  | 9-15 | [467], [15], [8] |
| Risedronat (Risedronic acid) |  |  |  | -^6^ | [49] |
| Risperidone^48^ | appr. 0.006^158^ (0.002-0.02^272^) | 0.12^311^ | 1.8^8^ | 2-4^159^ | [47], [457], [4], [640], [641] |
| Ritonavir | appr. 5-11 (-20) |  |  | 3-5 | [3], [642],[2] |
| Rivastigmine | 0.008-0.02 | 0.04^311^ |  | 1-2 (oral); appr. 3 (transdermal patch) | [4], [303], [15], [8], [643], [644] |
| Rizatriptan | appr. -0.1 |  |  | 2-3 | [3], [15], [8] |
| Rocuronium | appr. -17 |  |  | appr. 1.5 | [8] |
| Ropinirole | 0.0004 – 0.006^254^ | 0.012^311^ |  | 3-10 | [4], [8], [645] |
| Ropivacaine |  | (1-) 2^173^ |  | 2^168^ | [646], [647], [648] |
| Rosiglitazone | appr. 0.1-0.3 |  |  | 4 | [8] |
| Roxatidine | 0.1-0.8 |  |  | 5-6 | [8] |
| Roxithromycin | 4-12 |  |  | 12 | [115], [3] |
| Rufinamid | 5-30 | 40^311^ |  | 7 | [4] |
| Salbutamol (Albuterol) | 0.004-0.02 | (0.03-) 0.1-0.15^116^ | 0.16 | 3-6 | [11], [58], [649] |
| Salicylamide | 5-40 |  |  | appr. 1 | [8] |
| Salicylic acid | 20-200 | 300-350 | (400-) 500 | 3-20 | [28], [3], [29], [30], [31], [32], [33] |
| Salvinorin A |  |  |  | 40-80 min | [47] |
| Saquinavir (SQV) | > 0.1-0.25^296^ |  |  | (1-) 3-7 (-12) | [47], [110], [2], [111] |
| Scopolamine | 0.0001-0.0003 (-0.001) |  |  | appr. 3 | [15], [8], [11] |
| Secbutabarbital | 5-10 (-15) | 20 | 30 | 34-42 | [8] |
| Secobarbital | 1.5-5 | 7-10 | 10-15 | 15-30 | [87], [8] |
| Selegiline | see Amphetamine and Methamphetamine |  |  |  |  |
| Selenium | 0.045-0.13 (-0.19) | 0.4-1 | 2; (2.6^8^; 2.8^8; 304^; 18.4^8^; 38^8^) | 69-77^6^ | [47], [3], [650], [87], [651], [652] |
| Seratrodast | appr. 3-25 |  |  |  | [8] |
| Sertindole | 0.05-0.1 | 0.2^311^ |  | 55-90 | [8], [101], [4] |
| Sertraline | (0.01-) 0.05-0.25 | 0.29^8^ | 1.6^8^; 3^8^ | 24-28 (22-34) | [47], [467], [653], [15], [8], [11], [58], [223] |
| Sevoflurane | appr. 134^381^ |  | 8, 26^8^ | 1.8-3.8 | [47] |
| Sibutramine^366^ | appr. 0.001-0.01 |  |  | 4-8 | [47], [8] |
| Sildenafil | appr. 0.05-0.5 |  |  | 3-5 | [3], [15], [8] |
| Silver | -0.005^367^ |  |  |  | [58] |
| Simvastatin | 0.0027-0.0056 |  |  | appr. 2^290^ | [48] |
| Sirolimus | 0.005-0.015^244^ | 0.015(-0.06) |  | 57-63 | [123], [654], [655], [656], [657] |
| Sisomicin | 0.5-10 |  |  | appr. 1 | [8] |
| Sitagliptin | 0.05-0.38 |  |  | 8-14 | [47] |
| Sodium aurothiomalate (gold) | see Gold |  |  |  |  |
| Sodium nitroprusside | see Thiocyanate |  |  | 0.1 |  |
| Sodium oxybate (GHB) | see 4-Hydroxybutyrate |  |  |  |  |
| Sodium valproate | see Valproic acid |  |  |  |  |
| Sotalol^167^ | 0.5-3 (-4) | 7.5-16^8^ | 40^8^; 43^8^ | 5-13 (-17) | [658], [70], [7], [659] |
| Sparteine^48^ | 0.5-1 |  |  | 2.5 | [8] |
| Spiramycin | appr. –3 |  |  | 5-8 | [3], [660] |
| Spiraprilate | 0.006-0.045 |  |  | 33-41 | [8] |
| Spironolactone | (0.05-) 0.1-0.25 (-0.5)^73^ |  |  | 13-24^73^ | [8], [11], [58] |
| Stiripentol | 1-10 | 15(-20) |  | 4-13 | [4], [3], [8] |
| Streptomycin | 1-5 (15-40) | 40-50 |  | 2-4 | [467], [8], [11], [58] |
| Strontium | -0.03 |  |  | 30-60 | [47] |
| Strychnine |  | 0.075-0.1 | 0.2-2 | 10-15 | [661], [662], [663], [664], [15], [8], [665], [11], [666], [667] |
| Sufentanil | 0.0005-0.01^4^ | -^339^ | 0.001-0.007^8^ | 1.6-5.7 (7-49) | [47], [668], [669], [670], [344], [346], [671], [54] |
| Sulbactam | -80 |  |  | 1-2^70^ | [174], [3] |
| Sulfamethoxazole | 30-60^56^ | 200-400 |  | 9-12 | [3], [672] |
| Sulfasalazine^34^ | 5-30 (-70) |  | 130^8;304^ | 4-10 | [8] |
| Sulfisoxazole | 90-100 |  |  |  | [48] |
| Sulfinpyrazone | 6-17 |  |  | 3-5 | [8] |
| Sulindac | 1-5^102^ |  |  | appr. 7 | [673], [87] |
| Sulpiride | 0.05-0.4 (-1)^225^ | 1; 5^8^ | 3.8^8^; 38^8^ | 4-11 (-14) | [47], [43], [3], [4], [11], [58] |
| Sultiam (Sulthiame) | 0.5-12.5 (2-8) | 12-15 | 20-25 | 3-30 | [491], [8] |
| Sumatriptan | 0.018-0.06 |  |  | 2 | [15], [8] |
| Suramin | > 100^117^ | 300^118^ |  | 44-54 | [674] |
| 2,4,5-T | see 2,4,5-Trichloro-phenoxyacetic acid |  |  |  |  |
| Tacrine | appr. 0.01 |  |  | 2-4 | [303] |
| Tacrolimus (FK-506) | 0.005-0.015 (-0.02) | (0.015-) 0.02-0.025 |  | 9-16 | [11], [675], [676], [191], [677], [678], [679], [680], [681], [682], [683], [684]] |
| Tadalafil | 0.09-0.48 |  |  | 16-19 | [47] |
| Talinolol | 0.04-0.15 |  | 5^8; 129^; 20^8^ | 10-14 | [7], [685], [686] |
| Talipexole | appr. 0.0001-0.001 |  |  | 5-9 | [15], [8] |
| Tamoxifen | 0.05-0.5 |  |  | 5-7^6^ | [3] |
| Tapentadol | 0.05-0.13 |  | 0.3^8^; 2^8^ | 3-5 | [47] |
| Taxol | see Paclitaxel |  |  |  |  |
| Teicoplanin | (10-) 15-20 (-40) | 200 |  | 10-15; 83-168^83^ | [687], [688] |
| Temazepam | 0.02-0.15 (-0.9) | 1 | 8.2^8^; 14^8^ | 6-25 | [689], [59], [8], [11], [58] |
| Tenoxicam^174^ | appr. 5-10 |  |  | (50-) 70-90 | [690], [691] |
| Terazosin | appr. 0.02-0.08 |  |  | 8-12 | [15], [8] |
| Terbinafine | 0.01-0.03^205^ |  |  | 22-26 | [3], [10], [11], [58] |
| Terbutaline | 0.001-0.006 (-0.01) |  | 0.04 | 16-20^89^ | [8], [11], [58] |
| Terfenadine^368^ | < 0.01 | 0.04-0.06^148^ | 0.4^8^ | 15-22^64^ | [47], [3], [692] |
| Tetrachloroethylene |  |  | 4-5; 44^8^; 66^8^ | 33-72 | [87], [58] |
| Tetracycline | 1-5 (5-10) | 30 |  | 6-10 | [3], [8], [11] |
| Tetrazepam^40^ | 0.05-0.6 (-1) |  |  | (10-) 16-44 | [47], [59] |
| Thalidomide | 0.5-1.5 (-8) |  |  | 5-9 | [3], [15], [8] |
| Thallium | -0.002 (-0.0006^331^) | 0.1-0.5^179^; 5.6^8^ | 0.5-11 | -^6^ | [693], [3], [11], [694], [100] |
| Theobromine | 10-15 | 20 |  | 6-10 | [11], [58] |
| Theophylline | (5-) 8-15 (-20)^82^ | 20 | 50 | 6-9^41^ | [695], [696], [20], [697], [415], [698], [699], [21], [700], [701], [702], [703], [704], [705] |
| Thiamphenicol | 0.5-3-10 (-15) | 20 |  | 2-7 | [15], [8] |
| Thiamylal | appr. 5 |  | 29^8; 304^ | 0.6-0.8 (initial); 12-34 (terminal) | [47], [87] |
| Thiazinamium | 0.05-0.15 | 0.3 |  |  | [467], [15], [8], [11] |
| Thiocyanate from Nitroprusside | 1-12^144^ 5-30 | 35-50 50-100 | 200 | 3-4^6^ | [706], [3], [255], [11] |
| Thiopental^57^ | 1-5 | 7 | 10-15^58^ | 3-8 | [707], [48] |
| Thioproperazine | appr. 0.001-0.02 | 0.1 |  |  | [15], [8] |
| Thioridazine | 0.1-2 (0.2-0.8-1.25)^133^ | 2.5-5 | 3-10 | 7-13 (-36) | [56], [4] |
| Thiothixene | see Tiotixene |  |  |  |  |
| Thyroxine | see Levothyroxine |  |  |  |  |
| Tiagabine | 0.01-0.1 (-0.2) | 0.5-0.6; 3.1^8; 245^ |  | 7-9 (4-13) | [708], [3], [709], [710], [711], [12], [712], [15], [8], [381], [4] |
| Tiapride | C_max_ 1-2 | 4^311^ |  | appr. 3-4 | [4], [8] |
| Tiaprofenic acid | appr. 15-40^193^ |  |  | 1.5-3 (-6) | [3], [713], [714] |
| Ticlopidine | < 1-2 (?) |  |  | 70-130^100^ | [47], [8] |
| Tiletamine |  |  | 0.85^8; 304^ |  | [87] |
| Tilidine^25^ | 0.05-0.12 | -^339^ | 1.7^8^ | appr. 3 | [715], [716], [15] |
| Tiludronate (Tiludronic acid) | 0.2-1.5 |  |  | 65-78 (-150) | [49], [717] |
| Timolol | 0.005-0.05 (-0.1) |  |  | 2-6 | [8] |
| Tin | 0.03-0.14 |  |  |  | [58] |
| Tinidazol | max. -60 |  |  | 11-15 | [3] |
| Tiopronin | appr. 2-5 |  |  | 23 ± 11 | [718] |
| Tiotixene | 0.001-0.03 (0.002-0.014) | 0.1 |  | 34-36 | [185], [48] |
| Tiotropium | 0.000016^307^ |  |  | 5-6^6^ | [719] |
| Tipranavir (TPV) | > 20.5^299^ |  |  | 5.5-6 | [110], [2], [111] |
| Tizanidine | appr. 0.015 |  |  | appr. 2.5 | [3], [8] |
| Tobramycin | 4-10^154^ | 12-15 |  | 2-3 | [67], [3], [198], [41] |
| Tocainide | 4-12 (6-10) | 13-15; 20^8^ | 74^8^; 78^8^; 140^8^ | 8-25 | [47], [3], [720], [721] |
| Tofenacine | 0.025-0.1 | 0.5-1 |  |  | [9], [58] |
| Tolbutamide | 45-100 | 400-500 | 640^8^ | 4-12 | [722], [11], [58] |
| Tolcapone | C_max_ 3-6 | 12^311^ |  | 2 | [4] |
| Tolmetin | 10-80 |  |  | 2-4 | [11], [58] |
| Tolperisone | 0.09-0.3 | 0.7^8; 382^ | 7-14 | 1.8-2.9 | [47] |
| Toluene |  |  | 10 (-48^8^) | 13-68 | [47], [58] |
| Topiramate | 2-10^218^ | 16^311^ |  | 20-30 | [712], [15], [8], [381] |
| Topotecan | appr. 0.001-0.01^190^ |  |  | 2-3 | [3], [723], [724] |
| Tramadol^48^ | 0.1-1 (>0.3)^87^ | 1 | 2^8; 49^; 13^8^; 38.3^8; 252^ | 5-10 | [11], [244], [725], [726], [63] |
| Tranexamic acid | 10-50 |  |  | 10 | [8] |
| Tranylcypromine | < 0.05 | 0.1^311^; 0.5^8; 202^ | 0.7^8^; 5^8^ | 1-3.5 | [4], [727] |
| Trapidil | (4-) 6-10 |  |  | 2-6, 12 | [3], [95], [728] |
| Trazodone^145^ | 0.7-1 | 1.2^311^; 3-4 | 12-15^8^ | 4-11 (-13) | [43], [4], [729], [64] |
| Triamterene | 0.01-0.1 |  |  | 1.5-4 | [3], [42] |
| Triazolam | 0.002-0.02 | 0.04 |  | 2-5 | [730], [59], [423] |
| 2,2,2-Tribromoethanol |  | 50 | 90 |  | [58] |
| 1,1,1-Trichloroethane |  |  | (15^8^); 100-1000 | appr. 53 | [11], [58] |
| 2,2,2-Trichloroethanol^369^ | 5-15 | 40-70 | 60-100 | 6-10^327^ | [47], [11], [58] |
| Trichloroethylene |  |  | 9.7^8^; 16^8^; 21^8^ | 30-38 | [87] |
| 2,4,5-Trichlorophenoxyacetic acid (2,4,5-T) | - | appr. 100 | 200 | 23-33 | [3], [11] |
| Trifluoperazine | 0.001-0.01 (-0.05) | 0.1-0.2 | 0.4^8^ | 7-18 | [87], [8] |
| Triflupromazine | 0.03-0.1 | 0.3-0.5 |  | appr. 6 | [8] |
| Trihexyphenidyl | 0.05-0.2^75^ | 0.5 |  | 3-5 | [9], [114] |
| Trimeprazine | see Alimemazine |  |  |  |  |
| Trimethadione^274^ | 20-40 |  |  | 16 | [11], [58] |
| Trimethobenzamide | 1-2 |  | 184^8^ | 7-9 | [47], [11], [58] |
| Trimethoprim | 1.5-2.5^56^ | 20 |  | 8-11 | [3], [672] |
| Trimipramine | 0.01-0.3 | 0.6^311^ | 1.7-8.2^251^ | 10-20 (-40) | [56], [4], [223] |
| Tripelenamine | 0.02-0.06 |  | 10^8^ | 5-8 | [87], [15], [8], [57], [11] |
| Triprolidine | 0.004-0.045 |  |  | 2-5 | [57] |
| Tropisetron | 0.02-0.05 |  |  | 7-9 (-30) | [66], [8] |
| Tubocurarine | 0.04-6 |  |  | 2-4 | [48], [8], [58] |
| Tungsten | -0.035 |  |  |  |  |
| Uranium | 0.00004^331^ |  |  |  |  |
| Urapidil | appr. 0.1-0.2 |  |  | 2.7-7 | [3], [95] |
| Valdetamide | see Diethylpentenamide |  |  |  |  |
| Valnoctamide | 5-25 | 40 |  |  | [58] |
| Valproic acid | 40-100 (50-150) | 150-200 | 556^8^; 720^8^ | 8-20 | [47], [161], [164], [64] |
| Valsartan | appr. 0.8-6 |  |  | 6-9 | [8] |
| Vanadium | -0.05 |  |  | 4-12^6^ | [47] |
| Vancomycin | ≤ 5-10 (-12)^142^ | 30 |  | 2.6-11^83^ | [47], [67]. 1994), [390], [731], [732], [733], [734], [735], [736], [64] |
| Varenicline | 0.004-0.005 | 0.01^311^ |  | 24 | [4] |
| Vecuronium | appr. 0.2-0.37 (-0.5) |  |  | 1-1.5 | [3], [9] |
| Venlafaxine | 0.1-0.4^189^ | 1-1.5^266^ | 6.1-24^8^ | 3-7 | [47], [101], [4], [737], [11], [58], [223] |
| Verapamil^90^ | (0.01-) 0.02-0.25 (-0.4) | 1 | 2.5 ; 3.9^8^ | 6-14^42^ | [56], [84], [721], [738], [739], [64] |
| Vigabatrin | 2-10 (-15)^94^ | 20^311^ |  | 5-8 | [47], [381], [64] |
| Viloxazine | -6.0-8.0 (?) |  | 45^8^ | 2-5 | [43], [3], [15], [8] |
| Vincamine | < 0.25 (?) |  |  | 1-2 (8-17) | [8] |
| Vinylbital | 1-3 | 5 | 8 | 18-33 | [8] |
| Viquidil | 0.15-0.25 |  |  | 6-12 | [11], [58] |
| Vitamin A | see Retinol |  |  |  |  |
| Vitamin C | see Ascorbic acid |  |  |  |  |
| Vitamin D | (0.02-) 0.03-0.09^263^ | 0.2 |  | appr. 30^6^ | [740], [741], [742], [743] |
| Voriconazole | 2-6 | 3.5 (-6.0)^284^ |  | 4-10^37^ | [47], [603], [744], [745] |
| Warfarin | 1-3 (-7) | 10-12 | 100 | 37-50^97^ | [3], [42], [14], [746] |
| Wismut | see Bismut(h) |  |  |  |  |
| Xamoterol | appr. 0.02-0.04 (-0.1) |  |  | 7-8 | [3] |
| Xipamide | -20 |  |  | 5-8 | [3] |
| Xylene | 1.5 (BAT |  | 3-40 | 20-30 | [47], [11], [58] |
| Yohimbine | appr. 0.05-0.3 |  |  | 1-3 | [64], [15], [8] |
| Zafirlukast | 0.005-0.03 |  |  | 10 | [3], [15], [8] |
| Zalcitabine | appr. 0.1 (0.5 µmol/L) |  |  | 1-3^83^ | [290], [38] |
| Zaleplon | appr. 0.001-0.1 |  |  | 1 | [8] |
| Zanoterone | 0.1-0.5 |  |  |  | [747] |
| Zidovudine | 0.1-0.3 (-1)^51^ | 2-3 |  | 1-1.5 | [290], [748], [749], [3] [750], [38],[2] |
| Zinc | 0.6-1.3 | 2 | 42^8^ | 5-16 months | [47], [66] |
| Zipeprol | 0.1-0.7 |  | 5.8^8^; 10.6^8^; 31^8^ | 1.2 | [47], [57] |
| Ziprasidone | 0.05-0.2 | 0.4^311^ |  | 2-7 | [4], [3], [15], [8] |
| Zoledronat (Zoledronic acid) |  |  |  | (1-2) 40-385 (-189^6^) | [49], [751], [752], [753] |
| Zolmitriptan | appr. 0.007-0.01 |  |  | 2.5-3 | [15], [8] |
| Zolpidem | 0.08-0.15 (-0.2) | 0.5 | 2-4 | 2-3 | [754], ([56], [755], [3], [310], [15], [11]. [756] |
| Zomepirac | 0.1-4 |  | 152^8^ | 4-10 | [8], [87] |
| Zonisamide | 10--40 | 40-70 | 100^208^ | 50-70^209^ | [8], [757], [758],[712], [381] |
| Zopiclone | 0.01-0.05 | 0.15 | 0.6-1.8 | 3.5-8 | [56], ([3], [4], [15], [11] |
| Zotepine | 0.01-0.15 | 0.3^311^ |  | 13-16 | [3], [4] |
| Zuclopenthixol^48; 365^ | 0.004-0.05 (-0.1) | 0.1-0.3 |  | 15-25 | [4], [15], [14] |

Clinical categories used for grouping analytical data:

Therapeutic: blood-plasma/serum concentrations (in general, trough at steady state) observed following therapeutically effective doses; no or only minimal side effects (drugs); “normal”: concentrations associated with no or only minimal toxic effects (other xenobiotics).
Toxic: blood-plasma/serum concentrations which produce toxicity/clinically relevant side effects/symptoms.
Comatose-fatal: blood-plasma/serum (comatose) concentrations and whole blood (fatal) concentrations reported to have caused coma and death, respectively. Whether published data for deaths refer to levels measured ante-mortem or post-mortem (femoral or heart blood ) is often unknown.
In addition to specific references provided in the table, data were compared and contrasted against published review articles and textbooks [3,4,7-15,41-43,47,48,56-59,64,66,74,87,88,259,270,294,467,759-779] but not specifically indicated for every drug/substance, as well as supplemented with our experiences in clinical and forensic toxicology.

Abbreviations: BAT, biological tolerance value (in the work area); rec, recombinant; Ref., references; t_½_, in general, terminal elimination half-life (if not stated otherwise, see annotations)

.

**Annotations to Table 1**

| 1 | active metabolites of acebutolol: N-acetylacebutolol (t½: 9-14 h): therapeutic concentration 1-2.5 mg/L, comatose-fatal from appr. 90 mg/L, and diacetolol (t½: 8-13 h): therapeutic 0.65-4.5 mg/L, comatose-fatal from appr. 100 mg/L |
| --- | --- |
| 2 | as salicylic acid (for analgesic and antipyretic effect) |
| 3 | as digoxin |
| 4 | during mechanical ventilation |
| 5 | slow (poor) and rapid (extensive) acetylators (metabolisers) |
| 6 | days |
| 7 | active metabolites nortriptyline (see Table) and amitriptyline oxide  (t½: 1.5-3 h) |
| 8 | case report |
| 9 | in patients with impaired renal function in some cases up to 100 h |
| 10 | active metabolite 6-mercaptopurine (t½ : 1-1.5 h) |
| 11 | appr. 0.2 h for azathioprine |
| 12 | active metabolite carbamazepine-10,11-epoxide (t½: 5-16 h; usual plasma concentration range 0.2-2 mg/L) should be considered in case of intoxication |
| 13 | each sum carbromal(um) + carbromide (t½: 12-15 days) |
| 14 | each as trichloroethanol |
| 15 | active metabolite desmethyldiazepam = nordazepam (see Table) |
| 16 | nephrotoxic |
| 17 | active metabolite N-desmethylclobazam (therapeutic reference range: 0.3-3 mg/L; laboratory alert level^311^ 5 mg/L) |
| 18 | duration of pharmacological effects: 0.3-0.4 h; major metabolite: benzoylecgonine (t_½_: 5-6 h) |
| 19 | active metabolites nordazepam and oxazepam (see Table) |
| 20 | active metabolite nordothiepin (t½: 20-60 h) |
| 21 | active metabolite desmethyldoxepin (synonym nordoxepin, t½: 33-80 h) should be considered in case of intoxication |
| 22 | benzodiazepine antagonist |
| 23 | active metabolites |
| 24 | active metabolite desalkylflurazepam (t½: 74 ± 24 h) |
| 25 | active metabolite nortilidine (t½: 6 h), comatose-fatal plasma concentration: 4.4 mg/L^8^ |
| 26 | in some cases up to 80 h |
| 27 | active metabolite levomepromazine sulfoxide (t½ : 5-10 h) |
| 28 | t½ for biological effects; terminal t½:16-78 h |
| 29 | active metabolite desipramine (see Table) |
| 30 | active metabolites diazepam, nordazepam plus oxazepam (see Table) |
| 31 | active metabolite of mesuximide |
| 32 | sum of active metabolites |
| 33 | each as desacetylmetipranolol |
| 34 | active metabolite 5-aminosalicylic acid (mesalazine, see Table); rapid/slow acetylators of the primary metabolite sulfapyridine |
| 35 | in some cases longer |
| 36 | active metabolite oxyphenbutazone (t½ : 27-64 h) |
| 37 | dose dependent |
| 38 | active metabolite of procainamide |
| 39 | for the management of osteoporosis |
| 40 | active metabolites diazepam (see Table), nordiazepam (see Table), and nortetrazepam (t½: 25-51 h) |
| 41 | smokers: t½: 3-6 h |
| 42 | during steady state |
| 43 | astemizole plus desmethylastemizole |
| 44 | blood drug concentrations following therapeutically effective doses below detection limit |
| 45 | as decanoate (t½: 5-12 days) |
| 46 | in intensive care patients in some cases t½ 8-22 h |
| 47 | physiologic |
| 48 | rapid (extensive, EM) and slow (poor) metabolisers (PM; genetic polymorphism) |
| 49 | 6 month-old-child, appr. 15 h after 100 mg tramadol rectally |
| 50 | total labetalol: 0.7-5.0 mg/L |
| 51 | C_max_ 0.038 ± 0.006 mg/L after a single oral dose of 150 µg/kg in nine persons with onchocerciasis (t½: 56 ± 7 h) |
| 52 | as enalaprilat |
| 53 | duration of clinical effect: 3-5 h |
| 54 | product after hydrolysis |
| 55 | narcotic; analyzed during distribution phase |
| 56 | for pneumocystis carinii pneumonia (PcP) treatment: sulfamethoxazole 100-200 mg/L, trimethoprim 5-10 mg/L |
| 57 | metabolite: pentobarbital (see Table) |
| 58 | “narcotic” |
| 59 | higher with meningism (-25 mg/L); decreased protein binding in neonates results in increased unbound drug |
| 60 | each as 2-hydroxyflutamide (active and major metabolite) |
| 61 | active metabolite of allopurinol |
| 62 | active metabolite paracetamol (synonym acetaminophen, see Table) |
| 63 | active metabolites phenobarbital (see Table) and phenylethylmalonide (7-10 mg/L; t½: 16-50 h) |
| 64 | as active carboxylic acid metabolite = fexofenadine (t½: mean 15 h) |
| 65 | 1 mg oral alprazolam/day equals appr. a plasma concentration of 0.01 mg alprazolam/L during steady state. Usually higher doses/plasma concentrations are recommended for the treatment of phobias when compared to panic disorder/attacks |
| 66 | highly inter- and intraindividual variable kinetics; for children (therapeutically): 0.04-0.1 mg/L; active metabolite desmethylchlorpromazine |
| 67 | ≥ 0.25 µmol/L desirable for echinococcosis |
| 68 | mean: 27 h; for geriatric patients (> 65 years) in some cases increased to more than 90 h |
| 69 | active metabolite 2-hydroxydesipramine (t½: mean 18 h; in patients with impaired renal function several fold increased) |
| 70 | in patients with impaired renal function several fold increased |
| 71 | in colon tissue 0.8-1.8 h after 1 x 2 g i.v.: 94.0-7.4 µg/g |
| 72 | active metabolites 1-(5-hydroxyhexyl)-3,7-dimethylxanthine and 1-(3-carboxypropyl)-3,7-dimethylxanthine (t½: 1-1.6 h), among others, with 5 and 8 times, respectively, higher plasma levels than pentoxifylline |
| 73 | as canrenone (one of the active metabolites of spironolactone, t½: 1.3-1.4 h) |
| 74 | appr. 8 h after ingestion of probably 210 mg haloperidol and 1400 mg orphenadrine-HCl with life-threatening arrhythmias |
| 75 | data on effective plasma concentrations for Parkinson’s disease not proven |
| 76 | peak: 20-30 mg/L, trough: < 7 mg/L |
| 77 | for hypertension: 0.2-0.45 mg/L; for angina/coronary heart disease or arrhythmias: 0.3-0.8 mg/L |
| 78 | therapeutic concentration of the unbound fraction: 0.5-2 mg/L; therapeutic concentration of the metabolite desalkyldisopyramide < 5 mg/L (ratio of metabolite to the parent compound disopyramide guide to duration of therapy and possibly to the likelihood of toxicity) |
| 79 | in mmol/L (mEq/L, mval/L): 0.4-1.2 (0.6-1.4), toxic from 1.5 mmol/L |
| 80 | terminal elimination t½: 37 ± 6 h, increased in case of renal dysfunction |
| 81 | therapeutic concentration of the unbound fraction: 1-2.2 mg/L |
| 82 | for (sleep) apnea: 5-10 mg/L |
| 83 | increased in patients with impaired renal function |
| 84 | Cmax 3-5 h after 4 mg loperamide hydrochloride orally: 1-3 ng/mL |
| 85 | active metabolite N-desmethylclomipramine (t½: 21-65 h, mean: 40 h) |
| 86 | 12-36 h |
| 87 | post-operative (on-demand; i.v.): 0.02-1-2 mg/L (median: 0.29-0.92 mg/L) as minimal (analgesic) effective concentration; O-desmethyltramadol: 0.03-0.04 mg/L (median: 0.036 mg/L) |
| 88 | 10-36 h |
| 89 | 11-26 h |
| 90 | stereoselective metabolism (therapeutic concentration after oral application higher than after intravenous administration) |
| 91 | t½ for ß-phase of the elimination: 0.5-1 h |
| 92 | as albendazole sulfoxide (active metabolite) |
| 93 | t½ in slow (poor) metabolisers appr. 40 h |
| 94 | trough plasma concentration at steady state during 2 g twice daily orally (p.o.) appr. 9 mg/L; Cmax (0.8 h after 1 g orally): appr. 45 mg/L |
| 95 | as active metabolite methimazole (thiamazole) |
| 96 | mean 80 min |
| 97 | 15-85 h |
| 98 | plasma concentrations do not correspond with pharmacological effects |
| 99 | naltrexone plus 6-ß-naltrexone: 0.025-0.1 mg/L; plasma concentrations of the less potent major metabolite 6-ß-naltrexol (t½: 11-13 h) are usually 1.5-10 times higher |
| 100 | at steady state; 4-15 h after a single dose |
| 101 | sum rifampicin plus metabolites |
| 102 | sum sulindac plus metabolites (sulindac sulfide, t½: 15-18 h; t½ sulindac sulfone: 17-20 h) |
| 103 | abuse |
| 104 | sum carisoprodol plus meprobamate |
| 105 | 12-15 days for the metabolites |
| 106 | t½ for total platinum plasma concentrations: 20-40 h (up to 6-7 days) |
| 107 | 2-20 µmol/L |
| 108 | carboxylic acid metabolite (t½: appr. 20 days): 1.5-5.5 mg/L |
| 109 | during concomitant therapy with carbamazepine or phenytoin 13.5 -15 (8-33) h, during concomitant therapy with valproic acid 48-59 (31-89) h |
| 110 | in infants and after intoxications in some cases dramatically increased |
| 111 | during steady state 3-4 h after oral doses of 100-400 mg; prophylaxis of candidiasis: > 0.2 mg/L and of aspergillosis: > 1.0 mg/L in patients with acute myeloid leukemia (AML) |
| 112 | plasma concentrations of the major metabolite 13-cis-acitretin are usually higher |
| 113 | higher and increased, respectively, in patients with impaired hepatic function; for tinnitus aurium: therapeutic plasma concentration appr.  1-2 mg/L |
| 114 | biologically active/major metabolite cycloguanil (t½: 8-17 h): plasma concentration after daily oral doses of 100-200 mg proguanil appr. 0.02-0.06 mg/L |
| 115 | active metabolite norpethidine (t½: 14-24 (-48) h): toxic from appr. 0.5 mg/L |
| 116 | tremor, hypokalemia |
| 117 | as cytostatic drug: >200 mg/L |
| 118 | neurotoxic |
| 119 | in terminal renal insufficiency appr. 0.5-2 mg/L, cumulation of the inactive metabolite N-acetyl-5-aminosalicylic acid (Ac-5-ASA) up to 20 mg/L without adverse effects |
| 120 | t½ of the inactive major metabolite N-acetyl-5-aminosalicylic acid (Ac-5-ASA) appr. 6-9 h |
| 121 | tocolytic (4.5-6.25 mval(mEq)/L, 2.25-3.125 mmol/L). Approximate normal range: 18-25 µg Mg^2+^/mL (0.74-1.03 mmol/L); conversion factor: mg/dL x 0.4113 = mmol/L |
| 122 | Cmax appr. 2-8 µmol/L (i.e. 1.7-6.8 mg/L, after 170-275 mg/m^2^ intravenously for 6 h); much lower after intraperitoneal injection |
| 123 | as transdermal system (patch); plasma concentrations of the major metabolite cotinine (t½: mean 16-20 h) appr. 10 times higher |
| 124 | mean 2 h; after application of the transdermal system possibly longer |
| 125 | active metabolites desipramine (see Table), 2-hydroxyimipramine (t½: 6-18 h), and 2-hydroxydesipramine^69^ |
| 126 | 3-7 min after retrobulbar blockade: 0.5-1.1 mg/L |
| 127 | for myasthenia gravis |
| 128 | half maximal effective concentration (EC_50_) for analgesia: 0.0088 ± 0.0053 mg/L; EC_50_ for respiratory depression: 0.035 ± 0.022 mg/L |
| 129 | appr. 14 h after oral ingestion of 1.5 g |
| 130 | fluoxetine plus norfluoxetine; t½ of the active metabolite norfluoxetine: 4-16, mean 7-9 days |
| 131 | active metabolites 2-oxoquazepam (t½: 39 (28-43) h) and N-desalkyl-2-oxoquazepam (N-desalkylflurazepam, t½: 74 ± 24 h) |
| 132 | peak plasma concentration during steady state |
| 133 | range of plasma concentrations after therapeutically effective doses of thioridazine for the active metabolites mesoridazine (thioridazine-2-sulfoxide): 0.2-1.6 mg/L (t½: 10-14 h) and sulforidazine (thioridazine-2-sulfone): up to 0.6 mg/L (t½: 10-16 h) and for the inactive metabolite thioridazine-5(ring)-sulfoxide: 0.06-4 mg/L; probably, the best correlation exists between the plasma concentration of mesoridazine and the clinical response |
| 134 | usually sleep occurred with ≥ 0.1 mg/L; in infants and children (< 13 years): in some cases during mechanical ventilation up to 3 mg/L; α-hydroxymidazolam-glucuronide likely contributes in case of impaired renal function to prolonged sedation |
| 135 | plasma concentration range of the primary metabolite 1,5-dimethyl-3,3-diphenyl-2-ethylidene-pyrrolidine (EDDP) during steady state: 0.005-0.055 mg/L (daily oral methadone dose: 10-225 mg, mean 60 mg) |
| 136 | ratio clozapine/active metabolite N-desmethylclozapine (= norclozapine, t½: 19.2 ± 10.2 h ) usually 1.0-2.5 |
| 137 | maximum antiemetic effect at > 0.01 mg/L |
| 138 | active metabolite descarboethoxyloratadine (t½: 17-24 h): appr. 0.005-0.02 mg/L |
| 139 | 0.15 ± 0.05 ‰ per h |
| 140 | during chronic administration appr. 10-20 h (induction of own metabolism) |
| 141 | caution is warranted in case of concomitant use or intoxication with serotonin reuptake inhibitors (SSRI) such as citalopram, clomipramine (fluoxetine, paroxetine): possible serotonin syndrome |
| 142 | trough concentration; peak concentration: < 40 mg/L |
| 143 | distribution half-life: 0.3-0.5 (-1) h |
| 144 | non-smoker: 1-4 mg/L (17-69 µmol/L); smoker: 3-12 mg/L (52-206 µmol/L) |
| 145 | major active metabolite 1-m-chlorophenylpiperazine; plasma concentration appr. 1/10 compared to trazodone |
| 146 | plasma concentration for maximal cellular accumulation of the active form gemcitabine-5’-triphosphate |
| 147 | after topical nasal or ocular administration |
| 148 | Torsade de pointes, usually due to cytochrome P450 3A4 inhibition (e.g., ketoconazole, erythromycin) and/or impaired hepatic function |
| 149 | after oral administration; after topical application: plasma concentration < 0.03 mg/L and t½ appr. 22 h |
| 150 | for each added 1 mg/day dose of clonazepam, there is appr. an increase of 12 ng/mL in the plasma (patients with panic disorder) |
| 151 | Sum of amoxapine and his major metabolite 8-hydroxyamoxapine (t½: appr. 30 h; t½ 7-hydroxyamoxapine: 4-6.5 h) |
| 152 | sum bupropion (amfebutamone) and morpholinole metabolite (t½: 19-22 h) |
| 153 | after i.m.-application as decanoate appr. 3 weeks |
| 154 | C_min_ < 1-2 mg/L at best (especially in patients with renal dysfunction) |
| 155 | appr. 0.02 mg/L in organophosphorous ester poisoning depending on clinical symptoms |
| 156 | in case of organophosphorous ester (e.g. parathione) intoxication; 250 mg intravenously as bolus followed by an infusion of 750 mg/24 h |
| 157 | if used as an antiarrhythmic appr. 0.1-0.4 mg/L |
| 158 | using daily oral doses of ≤ 25 mg 0.00046 mg risperidone/L per mg dose and 0.0064 mg/L per mg dose for risperidone plus 9-hydroxyrisperidone (the clinical effects likely results from the combined concentrations) |
| 159 | extensive metabolisers; t½ for poor metabolisers: 20 h; t½ for risperidone plus 9-hydroxyrisperidone: 22-24 h |
| 160 | 6 case reports: post mortem 5.2-49 µg citalopram/g blood and 0.3-1.4 µg desmethylcitalopram/g blood |
| 161 | concentration/dose-values for extensive metabolisers: 0.025-0.688 (median 0.098) nmol/L per mg oral perphenazine, and 0.096-0.750 (median 0.195) nmol/L per mg oral perphenazine (mol wt 506.07) for poor metabolisers, respectively |
| 162 | two cases after ingestion of appr. 4 g moclobemide in combination with clomipramine (plasma concentration: 0.3-0.5 mg/L, i.e. toxic) |
| 163 | as R-enantiomer, mean: 9 mg/L |
| 164 | dosage: 50-55 mg/kg per day |
| 165 | appr. 2.5 h after ingestion of 50-100 mg amlodipine besylate with alcohol (263 mmol ethanol/L) |
| 166 | 0.101 mg/L 4 h after ingestion of 70 mg and 0.185 mg/L at 10.5 h, complicated by oxazepam ingestion |
| 167 | data for d,l-sotalol |
| 168 | after i.v.-application; t½: 4-7 h following epidural administration (appr. 4-5 h following intercostal block and appr. 8 h following brachial plexus blockade, respectively) |
| 169 | mean 19 h; t½ of oral ciclosporine microemulsion is appr. 8 h |
| 170 | a longer t½, up to 3.8 days, has been reported in elderly patients, up to 3.8 days |
| 171 | target range of activated partial thromboplastin time (aPTT) is prolongation of 50-70 sec; aPTT prolongation of more than 100 seconds has been associated with an increased risk of hemorrhagic events |
| 172 | as 10-hydroxycarbazepine for seizures (0.4-2 mg/L for oxcarbazepine) ; in patients with trigeminal neuralgia, therapeutic target range of the active metabolite 10-hydroxycarbazepine (t½: 7-14 (-20) h): 50-110 µmol/L (appr. 13-28 mg/L) |
| 173 | mild CNS symptoms (limited data) |
| 174 | pharmacologically inactive metabolites 5’ and 6’-hydroxytenoxicam |
| 175 | effective plasma concentrations for the 2 active metabolites: O-desmethylencainide (0.05-0.3; toxic from 0.3 mg/L, t½: 11 h) and 3-methoxy-o-desmethylencainide (0.06-0.28 mg/L; t½: > 24 h) during long- term therapy |
| 176 | in poor metabolisers 9-11 h |
| 177 | “normal”: 0.001-0.006; smoker: 0.005-0.012 (-0.15) mg/L; µmol/L x 0.026 = mg/L |
| 178 | reference value; 0.001 µg/g creatinine or 0.0014 mg/L urine; < 30 µg/24 h urine (“normal”); “toxic” from appr. 0.05-0.3 mg/L urine. Reference value for children in Germany: 0.0008 mg/L blood and 0.0004 mg/L urine |
| 179 | > 0.04 mg/L urine |
| 180 | up to years in chronically exposed workers |
| 181 | combination with 2,4-D and chlorpyrifos |
| 182 | in case of intoxication/overdose: 70-90 h |
| 183 | overdose |
| 184 | one case of toxicokinetic estimation in acute potassium cyanide (KCN) poisoning |
| 185 | dependent on indication; > 2.0 mg/L for partiell seizures; tentative target range according to Neels HM et al. 2004: 12-20 mg/L; peak concentration at steady state appr. 4.6 mg/L (300 mg three times daily (tid)) and appr. 8.4 mg/L (600 mg tid) |
| 186 | prolonged in case of impaired renal function to 16-43 h; > 100 h in dialysis dependent patients |
| 187 | dependent on urine pH, if alkaline appr. 8-10 h |
| 188 | females showed significantly longer elimination half-lives (35.4 **±** 13.7 h) than males (mean 21-26 h); the t½ of the R(-)-enantiomer is twice that of the S(+)-enantiomer |
| 189 | venlafaxine plus O-desmethylvenlafaxine. After doses of 25, 75, and 150 mg every 8 h for 3 days, mean peak serum levels were 0.053, 0.167, and 0.393 mg/L; corresponding levels of the major active metabolite O-desmethylvenlafaxine (t½: 10-11 h) were 0.148, 0.397, and 0.686 mg/L |
| 190 | at least 10 nmol of the lactone (mol wt 421.46)/L; decreases in absolute neutrophil counts of 50-90 % were observed with steady state plasma concentrations of total topotecan (lactone + hydroxy acid) of 20-60 nmol/L, respectively |
| 191 | a mean steady state peak plasma concentration of 0.286 mg/L was observed in healthy volunteers after 60 mg (oral solution) every 12 hours for 10 doses |
| 192 | the metabolite 2’,2’-difluorodeoxyuridine (dFdU) has minimal antitumor activity but may contribute to the toxicity of gemcitabine |
| 193 | C_max_ after 200 mg three times daily (tid) |
| 194 | serum concentration of benzoic acid following high dose diazepam i.v.-infusion and severe metabolic acidosis (5-year-old girl; urine concentration: 1,200 mg/L) |
| 195 | 1.5 h in dogs after i.v.-administration |
| 196 | for erythropoietic protoporphyria (EPP) |
| 197 | trough; peak: 0.1-0.5 mg/L |
| 198 | + 0.4 mg of its metabolite 3-deacetylpancuronium/L |
| 199 | “normal”: ≤ 2-3 % of total Hb; from 15-20 %: cyanosis, headache, dizziness |
| 200 | “normal”: ≤ 5 % hemoglobin as carboxyhemoglobin (elderly: -15 %); smoker: 8-10 % |
| 201 | 2 h after ingestion |
| 202 | 3 h after ingestion of 400 mg with no severe symptoms |
| 203 | mean steady state trough concentration; peak concentration: 5-15 mg/L |
| 204 | for Parkinson’s disease (appr. 15-50 pmol/mL) |
| 205 | peak: 0.5-3 mg/L |
| 206 | plasma concentrations below detection limit; plasma concentrations of the active metabolite 6-methoxy-2-naphthylacetic acid (t½: appr. 24 h), which appears to be responsible for the effects, were 10-37 mg/L 3-6 h after single oral doses of 250, 500, and 1000 mg, respectively |
| 207 | active metabolite 6-O-desmethyldonepezil |
| 208 | coma in a patient overdosing zonisamide, carbamazepine, and clonazepam |
| 209 | 25-30 h in patients co-medicated with enzyme-inducing anticonvulsants (e.g. phenobarbital) |
| 210 | 2-4 h in patients co-medicated with enzyme-inducing anticonvulsants (e.g. phenobarbital) |
| 211 | renal-transplant patients treated long-term (2-3 years) with mycophenolate mofetil had significantly lower trough plasma concentrations of the active metabolite mycophenolic acid (1.94 ± 0.24 mg/L) when compared to patients taking mycophenolate mofetil (1 g twice daily) short-term (2-10 months; 3.53 ± 0.45 mg/L). Proposed mycophenolic acid pre-dose target concentration: 1-3.5 mg/L |
| 212 | as mycophenolic acid (active metabolite) |
| 213 | ten men with multiple sclerosis, 10-20 mg p.o. every 6 h and analyzed 30 min before the next dose; peak levels < 0.1 mg/L 30 min after a dose |
| 214 | nine patients, maximum tolerated oral dose 50-100 mg |
| 215 | t_½_ of the metabolite 3-O-methyldopa: 15 h |
| 216 | appr. 2.5 µmol/l (1 mg/L) 24 h after single doses of 100-800 mg and during daily treatment with 200 mg |
| 217 | active metabolite 14-hydroxyclarithomycin (t_½_: 5-7 h) |
| 218 | C_max_ following oral administration of 200, 400, 800, and 1200 mg, respectively: 3.7, 8, 18, and 29 mg/L; tentative target range according to Neels et al., 2004: 5-25 mg/L |
| 219 | at a daily dosage of 60, 120, and 240 mg the mean ± SD concentration in patients with symptomatic ventricular tachyarrhythmias (n = 9-18) was 75 ± 46, 144 ± 105, and 324 ± 180 nmol/L, respectively |
| 220 | nonlinear kinetics |
| 221 | appr. 5 h after ingestion of 3 g, not associated with severe toxicity to a 27-year-old woman |
| 222 | slightly increased (8-12 h) in patients with impaired hepatic function; active metabolites hydroxynefazodone (t_½_: 2-5 h), m-chlorophenyl-piperazine (t_½_: 4-10 h), and triazoledione (t_½_: 10-12 h) |
| 223 | each as N-desmethylsuximide; methsuximide (t_½_: 1-2 h) steady state concentration: appr. 0.04-0.08 mg/L |
| 224 | mean steady state trough concentration in 15 young adults receiving a daily dose of 0.47-1.71 mg isotretinoin/kg: 0.05-0.34 mg/L (t_½_: 29 **±** 40 h), and for the 4-oxo metabolite (t_½_: 22 **±**10 h): 0.16-0.68 mg/L |
| 225 | for depression; higher in case of schizophrenia (0.2-1 mg/L) |
| 226 | suggested threshold for the sum of clomipramine (0.05-0.06 mg/L) and N-desmethylclomipramine (0.16-0.18 mg/L): 0.2-0.24 mg/L |
| 227 | for the active metabolite E-3174 (t_½_: 4-9 h); plasma concentration of losartan producing 50 % of maximal blood pressure response to exogenous angiotensin-II: 0.032 mg/L |
| 228 | as ramiprilat (t_½_: 13-17 (50-110) h) |
| 229 | half maximal inhibitory concentration (IC_50_) for analgesic effect after oral surgery |
| 230 | the inhibitory concentration to reduce the level of extracellular hepatitis B DNA by 50 % varied from 2.3 µg/L to 1.3 mg/L; C_max_ after 150 – 300 mg *per os* (p.o.): 1.2-2.0 mg/L |
| 231 | C_max_ at steady state (666 mg three times daily (tid) *per os* (p.o.)*)* |
| 232 | after oral administration of the enteric-coated tablet |
| 233 | trough < 2 plus peak 6-10 (5-12) mg/L |
| 234 | reference value; < 0.015 mg/L urine |
| 235 | active metabolite 4’-hydroxynimesulide (t_½_: 3-9 h) |
| 236 | mean C_max_ 126.5 and 226.3 ng/mL 2 h after 75 and 125 mg p.o. and 162.9 and 291.8 ng/mL after oral administration of 1.0 and 1.6 mg MDMA/kg body weight, respectively, to young adults; mean C_max_ for the metabolites 4-hydroxy-3-methoxymethamphetamine (HMMA) 171.9 and 173.5 ng/mL, 3,4-methylenedioxymethamphetamine (MDA) 8.4 and 13.8 ng/mL, and 4-hydroxy-3-methoxyamphetamine (HMA) 3.5 and 3.9 ng/mL, respectively |
| 237 | in patients > 60 years prolonged up to 10 h |
| 238 | adjuvant in methadone maintenance therapy |
| 239 | means of the ‘average’ steady state plasma concentration for the relatively high dose of 250 mg q8h (every 8 hours) appr. 0.4-0.6 mg/L |
| 240 | combination of distribution and elimination processes |
| 241 | as active metabolite fenofibric acid |
| 242 | appr. 37.5 mmol/L (=mval/L, mEq/L) |
| 243 | steady state concentration 21.6 ± 14.2 mg/L (mean ± SD) during continuous infusion of 3 g (1.1-2.2 mg/kg h) every 24 hours in 44 patients undergoing coronary artery bypass graft surgery |
| 244 | target trough concentration if ciclosporine is being used at trough concentrations of 0.075-0.15 mg/L; without ciclosporine: appr. 0.03 mg/L (LC/UV assay) |
| 245 | 4 hours after ingestion of 30-40 tiagabine HCl 8 mg tablets (coma) |
| 246 | bupropion plus 10-hydroxybupropion (t_½_: 17-47 h) |
| 247 | calculated steady state concentration in children (4 months to 16 years) receiving 0.3 mg/kg body weight i.v. |
| 248 | femoral blood concentration of the metabolite desmethylalimemazine after fatal intoxication: 0.2-1.3 µg/g |
| 249 | 40-50 min after 0.15 mg/kg i.v. |
| 250 | femoral blood concentration of the metabolite desmethylpromethazine after fatal intoxication (n=3): 0.3-1.8 µg/g |
| 251 | femoral blood concentration of the metabolite desmethyltrimipramine after fatal intoxication (n=10): 0.3-2.5 µg/g |
| 252 | fatal overdose with tramadol, alprazolam (0.21 mg/L), and alcohol (1.29 g/kg) in a 30-year-old woman |
| 253 | enterohepatic circulation; prolonged in elderly subjects to 33.4 hours (range: 20.0-53.4 h) |
| 254 | C_min_/D [(ng/mL)/mg], i.e. dose-normalized trough plasma drug concentration, dosage interval 8 h |
| 255 | all data refer to the active metabolite A771726 |
| 256 | steady state concentrations at 5, 10, and 25 mg/d, respectively |
| 257 | steady state trough concentrations after 400 mg/d orally; two major metabolites modafinil acid (appr. 0.5-0.8 mg/L, t_½_: 7.3 ± 1.1 h) and modafinil sulfone (appr. 4.5-5.3 mg/L), but neither appears to contribute to the wake-promoting properties of modafinil |
| 258 | mean plasma trough concentration at steady state obtained from 400 mg imatinib/day in 83 adult patients with chronic phase CML; peak: 2.3 mg/L |
| 259 | in a 5-year-old girl |
| 260 | suggested minimum target trough concentration in patients with HIV-1 susceptible to the antiretroviral (ARV) drug (dose of 800 mg two times daily (bid)) |
| 261 | active metabolite N-desethylamiodarone (t_½_: 57-64 days), which achieves plasma concentrations similar to the parent compound |
| 262 | inactive metabolites deshydroxyethyl opipramol (t_½_: 97 ± 24 h) and opipramol N-oxide (t_½_: 10.7 ± 3.2 h) |
| 263 | as 25-hydroxyvitamin D (25(OH)D, calcidiol); vitamin D deficient: < 0.01 mg/L (< 10 ng/mL = 25 nmol/L); vitamin D insufficient: < 0.02 – 0.03 mg/L (50-75 nmol/L); conversion factor: mg/L x 2,500 = nmol/L (ng/mL x 2.5 = nmol/L) |
| 264 | 6 h after reportedly ingestion of 30 g in a 38-year-old woman |
| 265 | metabolite perindoprilat, 3 to 10 hours, with a prolonged terminal half-life between 25 to 120 h |
| 266 | sum venlafaxine and O-desmethylvenlafaxine |
| 267 | for glaucoma 4-5 mg/L |
| 268 | doxapram + keto-doxapram |
| 269 | 24 h after ingestion of appr. 20 mL |
| 270 | active metabolite desethylamodiaquine (t_½_: 1-10 days) |
| 271 | smokers: -0.0006 mg/L |
| 272 | 0.01-0.1 mg/L for 9-hydroxyrisperidone |
| 273 | as active metabolite after administration of therapeutic doses of diazepam |
| 274 | active metabolite dimethadione (see Table) |
| 275 | HPLC-MS/MS (or FPIA) blood, in combination with ciclosporine micro-emulsion |
| 276 | n=439; median in µmol/L (interquartile range) |
| 277 | n=264; median in µmol/L (interquartile range) |
| 278 | in the presence of ethanol or during ethanol treatment ; t_½_ longer in patients with a serum creatinine concentration ≥ 130 µmol/L |
| 279 | n=99; median in µmol/L (interquartile range) |
| 280 | 3 dead infants aged ≤ 6 months with post mortem blood levels of pseudoephedrine ranging from 4.7 to 7.1 mg/L |
| 281 | daily dose 2-8 mg *per os* (p.o.) |
| 282 | treatment goal: cystine levels <1 nmol cystine/mg protein |
| 283 | according to other sources: < 0.005 mg/L; urine: < 0.05 mg/L; reference value for children in Germany: 0.015 mg/L urine. Case report: 0.13-0.16 mg/L (urine: 67.5 mg/L) on the first day after ingestion of appr. 54 g arsenic trioxide |
| 284 | potentially increased risk for visual adverse effects (> 3.5 mg/L) and abnormal liver function, respectively |
| 285 | trough plasma buprenorphine and norbuprenorphine concentrations in excess of 0.0007 mg/L were associated with minimal withdrawal symptoms in 11 heroin-dependent subjects |
| 286 | serum peak concentration of misoprostol acid (MPA) 574.8 ± 250.7, 287.6 ± 144.3, and 125.2 ± 53.8 pg/mL after sublingual, oral, and vaginal application, respectively, of 0.4 mg misoprostol to 40 women undergoing termination of pregnancy |
| 287 | on the first day of hospital admission after unintentional ingestion of appr. 400-500 mg carbachol (corresponding urine concentration: 374 mg/L). |
| 288 | main (probably inactive) metabolite: morphine-3-glucuronide (M3G); active metabolite: morphine-6-glucuronide (M6G) |
| 289 | metabolite: nordoxylamine |
| 290 | active ß-hydroxy-metabolite |
| 291 | targeted range between AUC of 9-12 mg/L/h |
| 292 | as amprenavir; suggested minimum target trough concentration in patients with HIV-1 susceptible to the antiretroviral (ARV) drug (dose of 700 mg bid (twice daily)) |
| 293 | suggested minimum target trough concentration in patients with HIV-1 susceptible to the antiretroviral (ARV) drug (dose of 300 mg qd (once daily)) |
| 294 | suggested minimum target trough concentration in patients with HIV-1 susceptible to the antiretroviral (ARV) drug (dose of 400 mg bid (twice daily)) |
| 295 | suggested minimum target trough concentration in patients with HIV-1 susceptible to the antiretroviral (ARV) drug (dose of 1,250 mg bid (twice daily)) |
| 296 | suggested minimum target trough concentration in patients with HIV-1 susceptible to the antiretroviral (ARV) drug (dose of 1,000 mg bid (twice daily)) |
| 297 | suggested minimum target trough concentration in patients with HIV-1 susceptible to the antiretroviral (ARV) drug (dose of 600 mg qd (once daily)) |
| 298 | suggested minimum target trough concentration in patients with HIV-1 susceptible to the antiretroviral (ARV) drug (dose of 200 mg bid (twice daily)) |
| 299 | suggested minimum target trough concentration for antiretroviral therapy-experienced patients who have resistant HIV-1 strains (dose of 500 mg bid (twice daily)) |
| 300 | suggested minimum target trough concentration for antiretroviral therapy-experienced patients who have resistant HIV-1 strains |
| 301 | median (range) trough concentration from clinical trials (dose 600 mg bid (twice daily)); suggested threshold: > 0.55 mg/L |
| 302 | median (range) trough concentration from clinical trials |
| 303 | median (range) trough concentration from clinical trials |
| 304 | post-mortem heart blood level (death by hanging?) |
| 305 | active enantiomer of propoxyphene,; active metabolite norpropoxyphene |
| 306 | heart blood |
| 307 | C_max_ at steady state achieved after 2-3 weeks of once-daily inhalation of 18 µg tiotropium; t_max_ after inhalation of 18 µg: 5 min |
| 308 | steady state peak concentration following a 300 mg twice-daily or a 600 mg once-daily regimen |
| 309 | active metabolite cis-monohydroxyperhexiline (t½: 10-29 h) |
| 310 | 1-2 h after 50 mg |
| 311 | “laboratory alert level” according to AGNP Consensus Guidelines for therapeutic drug monitoring (TDM) in psychiatry: update 2011 i.e., drug concentrations above the recommended reference range, based on reports on intolerance or intoxications. In most cases, however, arbitrarily defined as plasma concentration that is 2-fold higher than the upper limit of the therapeutic reference range |
| 312 | active metabolite 6-hydroxybuspirone |
| 313 | C_max_ 1-2 h after 4 mg |
| 314 | at low dose therapy (2.5 mg); at maximum dose (25 mg): 0.001-0.004 mg/L |
| 315 | C_max_ 0.5-4 h after drug intake for 4 weeks |
| 316 | C_max_ after 2 h |
| 317 | 60-90 min after intake of 1.2 mg/kg per day |
| 318 | 4 h after 20 mg |
| 319 | t_½_ with ritonavir 15-23 h |
| 320 | active metabolite 8-hydroxyloxapine (t_½_: 20-60 h) |
| 321 | C_max_ after 2h-infusion of 6 mg: 0.328 mg/L, after infusion of 2 mg: 0.246 mg/L |
| 322 | mean C_max_ after 2h-infusion of 10 mg: 0.265 mg/L |
| 323 | mean C_max_ after 4h-infusion of 15 mg: appr. 0.25 mg/L |
| 324 | male; female: -0.07 mg/L, children: -0.06 mg/L (reference value for children in Germany: 0.035 mg/L (whole) blood) |
| 325 | shorter in case of hemodialysis or continuous venovenous hemodiafiltration (CVVHDF); in a, fatal, case with 4,400 mg methanol/L blood and in the presence of adequate ethanol level (1,000 mg/L or 1 ‰) appr. 3.5 h |
| 326 | after topical (dermal) application |
| 327 | prolonged in newborns (27.8 ± 21.3 h) |
| 328 | reference value for Germany: 0.014 mg/L urine |
| 329 | reference value for children in Germany: 0.0003 mg/L urine |
| 330 | reference value for children in Germany: 0.0045 mg/L urine |
| 331 | reference value in urine for children in Germany |
| 332 | reference value in whole blood in Germany |
| 333 | metabolite of 3,4 methylenedioxymeth(yl)amphetamine (MDMA) |
| 334 | t_½_ (R)-MDE: 7.9 (6-11) h; t_½_ (S)-MDE: 4.0 (3-6) h |
| 335 | 40 h after oral ingestion of appr. 100 mg (0.03 mg/L 60 h after drug intake) |
| 336 | active metabolite phenytoin |
| 337 | prodrug; main active metabolites are morphine and morphine-6-glucuronide (M6G); main (probably) inactive metabolite = morphine-3-glucuronide (M3G) |
| 338 | in maintenance therapy e.g., for heavily dependent opioid addicts. 30 min after i.v- application of 150-300 mg diacetylmorphine: 0.1-0.24 mg/L morphine (t_½_: 1-4 h), 2.6-5.9 mg/L morphine-3-glucuronide (M3G; t_½_: (2-) 3-5 h), 0.5-1.0 mg/L morphine-6-glucuronide (M6G; t_½_: (1-) 2-3 h), and 0.08-0.29 mg/L 6-monoacetylmorphine (6-MAM; t_½_: appr. 2-5 min; N = 4); in another study 30 min after i.v- application of 260-300 mg diacetylmorphine: 0.39-0.75 mg/L morphine, 3.2-5.2 mg/L M3G, 0.5-0.7 mg/L M6G, and 0.08-0.19 mg/L 6-MAM (N = 4) |
| 339 | depending on tolerance and state/severity of pain |
| 340 | metabolites: norbuprenophine (active; t_½_: 35.6 (1.1-66.8) h after i.v., 73.6 (13.4-143) h after buccal, and 83 (10-243) h after sublingual application), buprenorphine-glucuronide, and norbuprenorphine-glucuronide |
| 341 | active enantiomer of zopiclone |
| 342 | active enantiomer of methylphenidate |
| 343 | active enantiomer of citalopram |
| 344 | strongly dependent on pH of urine |
| 345 | symptomatic poisoning in adults is more likely with doses above 90 mg |
| 346 | on hospital day #2 |
| 347 | 19 h post-ingestion of appr. 4 g |
| 348 | 5 h post-ingestion; all patients with a plasma paraquat level above 3.44 mg/L died |
| 349 | prolonged in (paraquat-induced) renal failure to appr. 80-120 (-150) h |
| 350 | active enantiomer of moramide |
| 351 | active enantiomer of fenfluramine |
| 352 | active enantiomer of methadone |
| 353 | after a bolus dose of 0.25 mg/kg body weight (N=10): 2.3 mg/L at 3 min, 0.84 mg/L at 30 min, 0.61 mg/L at 1 h, and 0.44 mg/L at 2 h |
| 354 | active metabolite oxypurinol |
| 355 | 11 h after ingestion |
| 356 | t_½_ in poor metabolisers of cytochrome P450 (CYP) 2D6 is appr. 21 h |
| 357 | metabolite of cocaine |
| 358 | dependent on pH of urine |
| 359 | sum of dibenzepine and desmethyldibenzepine |
| 360 | active metabolite of trimethadione |
| 361 | metabolite: acetone |
| 362 | in non-users of opioids/opiates |
| 363 | active metabolite norlorcainide (t_½_: 28-32 h), therapeutic plasma concentration: 0.1-1.5 mg/L |
| 364 | metabolite of azathioprin |
| 365 | (cis-) isomer of clopenthixol |
| 366 | active metabolites norsibutramine (t_½_: 12-22 h) and dinorsibutramine (t_½_: 14-23 h) |
| 367 | with silver sulphadiazine ointment for burns: 0.06-0.6 mg/L (non-toxic) |
| 368 | active metabolite fexofenadine |
| 369 | as metabolite of chloralhydrate |
| 370 | peak plasma concentration (C_max_) after 1 h chewing khat leaves that supplied 32 mg cathine |
| 371 | higher for poor metabolisers (PM) of cytochrome P450 (CYP) 2C9 |
| 372 | enantiomer of loratadine |
| 373 | 0.001-0.0035 mg/L for the active metabolite 3-hydroxydesloratadine ; t_½_: 17-27 h) |
| 374 | enantiomer of ketoprofen |
| 375 | after a single oral dose of 2 mg |
| 376 | average of 12 victims |
| 377 | serum morphine (active metabolite) levels were appr. 0.013 (extensive metabolisers, EM) and 0.003 (poor metabolisers, PM) |
| 378 | enantiomer of cetiricine |
| 379 | endogenous: -0.00001-0.00009 mg/L |
| 380 | active metabolite of psilocybin |
| 381 | during anaesthesia |
| 382 | measured 14 h post-ingestion |

List of abbreviations:
appr., approximately; t_½_, in general, terminal elimination half-life (if not stated otherwise); C_min_, minimum (trough) plasma/serum concentration (usually at steady state); C_max_ , maximum (peak) plasma/serum concentration; t_max_, time to peak concentration (C_max_); SD, standard deviation; mol wt, molecular weight; AUC, area under the (plasma concentration-time) curve

**References**

1. Yuen GJ, Weller S, Pakes GE: **A review of the pharmacokinetics of abacavir.** *Clin Pharmacokinet* 2008, **47:** 351-371.

2. University of Liverpool and eMedFusion. University of Liverpool and eMedFusion. http://www.hiv-druginteractions.org/ (last accessed May 21, 2012)

3. **Micromedex**® **Healthcare Series.** DRUGDEX® System. Greenwood Village, Colo: Thomson Reuters (Healthcare) Inc. Updated periodically.

4. Hiemke C, Baumann P, Bergemann N, Conca A, Dietmaier O, Egberts K *et al*.: **AGNP consensus guidelines for therapeutic drug monitoring in psychiatry: update 2011.** *Pharmacopsychiatry* 2011, **44:** 195-235.

5. Saivin S, Hulot T, Chabac S, Potgieter A, Durbin P, Houin G: **Clinical pharmacokinetics of acamprosate.** *Clin Pharmacokinet* 1998, **35:** 331-345.

6. Rooney M, Massey KL, Jamali F, Rosin M, Thomson D, Johnson DH: **Acebutolol overdose treated with hemodialysis and extracorporeal membrane oxygenation.** *J Clin Pharmacol* 1996, **36:** 760-763.

7. Schulz M, Meyer W, Schmitz W, Scholz J, Schmoldt A: **Beta-Rezeptorenblocker. Grundlagen zur Arzneimittelauswahl für eine rationale Therapie.** *Arzneimitteltherapie* 1989, **7:** 169-176.

8. Regenthal R, Krueger M, Koeppel C, Preiss R: **Drug levels: therapeutic and toxic serum/plasma concentrations of common drugs.** *J Clin Monit Comput* 1999, **15:** 529-544.

9. Meyer FP: **Indicative therapeutic and toxic drug concentrations in plasma: a tabulation.** *Int J Clin Pharmacol Ther* 1994, **32:** 71-81.

10. Uges DRA: **Referentiewaarden van xenobiotica in humaan material.** *Pharm Weekbl* 1995, **130:** 180-204.

11. Uges DRA: *Orientierende Angaben zu therapeutischen und toxischen Konzentrationen von Arzneimitteln und Giften in Blut, Serum oder Urin*. Weinheim: VCH; 1990.

12. Neels HM, Sierens AC, Naelaerts K, Scharpe SL, Hatfield GM, Lambert WE: **Therapeutic drug monitoring of old and newer anti-epileptic drugs.** *Clin Chem Lab Med* 2004, **42:** 1228-1255.

13. Schulz M, Schmoldt A: **Zusammenstellung therapeutischer und toxischer Plasmakonzentrationsbereicht von Arzneistoffen.** *Anaesthesist* 1994, **43:** 835-844.

14. Schulz M, Schmoldt A: **Therapeutic and toxic blood concentrations of more than 500 drugs.** *Pharmazie* 1997, **52:** 895-911.

15. Regenthal R, Krüger M, Köppel C, Preiß R: **Zu Möglichkeiten und Grenzen von therapeutischen und klinisch-toxikologischen Referenzwerten für Plasma-/Serum-/Vollblutkonzentrationen von Arzneimitteln bei akuten Vergiftungen - eine Übersicht.** *Anästhesiol Intensivmed* 1999, **40:** 129-144.

16. Wang G, Maranelli G, Perbellini L, Raineri E, Brugnone F: **Blood acetone concentration in "normal people" and in exposed workers 16 h after the end of the workshift.** *Int Arch Occup Environ Health* 1994, **65:** 285-289.

17. Zettinig G, Watzinger N, Eber B, Henning G, Klein W: **Überlebte Vergiftung nach Einnahme der zehnfachen Letaldosis von Aceton.** *Dtsch Med Wochenschr* 1997, **122:** 1489-1492.

18. Adams KF, Jr., Patterson JH, Gattis WA, O'Connor CM, Lee CR, Schwartz TA *et al*.: **Relationship of serum digoxin concentration to mortality and morbidity in women in the digitalis investigation group trial: a retrospective analysis.** *J Am Coll Cardiol* 2005, **46:** 497-504.

19. Dobbs RJ, O'Neill CJ, Deshmukh AA, Nicholson PW, Dobbs SM: **Serum concentration monitoring of cardiac glycosides. How helpful is it for adjusting dosage regimens?** *Clin Pharmacokinet* 1991, **20:** 175-193.

20. El Desoky E, Meinshausen J, Buhl K, Engel G, Harings-Kaim A, Drewelow B *et al*.: **Generation of pharmacokinetic data during routine therapeutic drug monitoring: Bayesian approach vs. pharmacokinetic studies.** *Ther Drug Monit* 1993, **15:** 281-288.

21. Hoppe MM, Iafrate RP, Hendeles L, Neims A: **A pediatric drug dosing and monitoring guide.** *Fl J Hosp Pharm* 1988, **8:** 259-269.

22. Josune I, Victoria CM, Mar AM, Dominguez-Gil HA: **Impact of written guidelines on the appropriateness of serum digoxin concentrations.** *Ann Pharmacother* 1993, **27:** 791-792.

23. Mordel A, Halkin H, Zulty L, Almog S, Ezra D: **Quinidine enhances digitalis toxicity at therapeutic serum digoxin levels.** *Clin Pharmacol Ther* 1993, **53:** 457-462.

24. Rathore SS, Curtis JP, Wang Y, Bristow MR, Krumholz HM: **Association of serum digoxin concentration and outcomes in patients with heart failure.** *JAMA* 2003, **289:** 871-878.

25. Terra SG, Washam JB, Dunham GD, Gattis WA: **Therapeutic range of digoxin's efficacy in heart failure: what is the evidence?** *Pharmacotherapy* 1999, **19:** 1123-1126.

26. Ujhelyi MR, Colucci RD, Cummings DM, Green PJ, Robert S, Vlasses PH *et al*.: **Monitoring serum digoxin concentrations during digoxin immune Fab therapy.** *DICP* 1991, **25:** 1047-1049.

27. Wells TG, Young RA, Kearns GL: **Age-related differences in digoxin toxicity and its treatment.** *Drug Saf* 1992, **7:** 135-151.

28. Cham BE, Johns D, Bochner F, Imhoff DM, Rowland M: **Simultaneous liquid-chromatographic quantitation of salicylic acid, salicyluric acid, and gentisic acid in plasma.** *Clin Chem* 1979, **25:** 1420-1425.

29. Herren T, Como F, Krahenbuhl S, Wyss PA: **Die Therapie der akuten Salizylatintoxikation.** *Schweiz Med Wochenschr* 1993, **123:** 1775-1783.

30. Irey NS, Froede RC: **Evaluation of deaths from drug overdose. A clinicopathologic study.** *Am J Clin Pathol* 1974, **61:** 778-784.

31. Mandelli M, Tognoni G: **Monitoring plasma concentrations of salicylate.** *Clin Pharmacokinet* 1980, **5:** 424-440.

32. Martens J, Meyer FP: **Besondere Eignung eines photometrischen Verfahrens zur Bestimmung von Salicylsäure im Therapeutischen Drug Monitoring.** *Pharmazie* 1995, **50:** 41-43.

33. Pond SM, Armstrong JG, Henderson A: **Late diagnosis of chronic salicylate intoxication.** *Lancet* 1993, **342:** 687.

34. Watson JE, Tagupa ET: **Suicide attempt by means of aspirin enema.** *Ann Pharmacother* 1994, **28:** 467-469.

35. Larsen FG, Jakobsen P, Knudsen J, Weismann K, Kragballe K, Nielsen-Kudsk F: **Conversion of acitretin to etretinate in psoriatic patients is influenced by ethanol.** *J Invest Dermatol* 1993, **100:** 623-627.

36. Sommerburg C, Bauer R, Orfanos CE, Petres J, Thiele B, Ulrich REH: **Therapeutische Wirksamkeit und neue Daten zur Pharmakokinetik von Acitretin.** *Dt Dermatol* 1994, **42:** 1316-1327.

37. Friedman D, Weller S, Dix L: **Acyclovir plasma concentrations and duration of herpes zoster pain: higher levels associated with the greater efficacy achieved with Valtrex (valacyclovir HCl).** In: *34. Int. Conf. AAC*; 1993:A72.

38. Morse GD, Shelton MJ, O'Donnell AM: **Comparative pharmacokinetics of antiviral nucleoside analogues.** *Clin Pharmacokinet* 1993, **24:** 101-123.

39. Shibata N, Kitamura A, Yoshikawa Y, Inoue T, Bamba T, Takada K: **Simultaneous determination of aciclovir and ganciclovir in plasma by HPLC and pharmacokinetic interactions.** *Pharm Pharmacol Commun* 2000, **6:** 501-506.

40. Committee for Medicinal Products for Human Use (CHMP). **Humira (adalimumab) EPAR - Product information.** [European Medicines Agency] (last accessed November 17, 2011)

41. Sonntag O: *Arzneimittel-Interferenzen*. Stuttgart - New York: Thieme; 1985.

42. Repetto MR, Repetto M: **Therapeutic, toxic, and lethal concentrations in human fluids of 90 drugs affecting the cardiovascular and hematopoietic systems.** *J Toxicol Clin Toxicol* 1997, **35:** 345-351.

43. Dinnendahl V, Fricke U: *Arzneistoff-Profile. Basisinformation über arzneiliche Wirkstoffe*. Eschborn: Govi; 2006.

44. Marriner SE, Morris DL, Dickson B, Bogan JA: **Pharmacokinetics of albendazole in man.** *Eur J Clin Pharmacol* 1986, **30:** 705-708.

45. Mirfazaelian A, Dadashzadeh S, Rouini MR: **An HPLC method for determination of albendazole main metabolites.** *Pharm Pharmacol Commun* 2000, **6:** 563-566.

46. Zeugin T, Zysset T, Cotting J: **Therapeutic monitoring of albendazole: a high-performance liquid chromatography method for determination of its active metabolite albendazole sulfoxide.** *Ther Drug Monit* 1990, **12:** 187-190.

47. Baselt RC: *Disposition of toxic drugs and chemicals in man*, 9 edn. Seal Beach: Biomedical Publications; 2011.

48. Winek CL, Wahba WW, Winek CL, Jr., Balzer TW: **Drug and chemical blood-level data 2001.** *Forensic Sci Int* 2001, **122:** 107-123.

49. Cremers SC, Pillai G, Papapoulos SE: **Pharmacokinetics/pharmacodynamics of bisphosphonates: use for optimisation of intermittent therapy for osteoporosis.** *Clin Pharmacokinet* 2005, **44:** 551-570.

50. Porras AG, Holland SD, Gertz BJ: **Pharmacokinetics of alendronate.** *Clin Pharmacokinet* 1999, **36:** 315-328.

51. Cocquyt V, Kline WF, Gertz BJ, Van Belle SJ, Holland SD, DeSmet M *et al*.: **Pharmacokinetics of intravenous alendronate1.** *J Clin Pharmacol* 1999, **39:** 385-393.

52. Lemmens HJ: **Pharmacokinetic-pharmacodynamic relationships for opioids in balanced anaesthesia.** *Clin Pharmacokinet* 1995, **29:** 231-242.

53. Maitre PO, Vozeh S, Heykants J, Thomson DA, Stanski DR: **Population pharmacokinetics of alfentanil: the average dose-plasma concentration relationship and interindividual variability in patients.** *Anesthesiology* 1987, **66:** 3-12.

54. Scholz J, Steinfath M, Schulz M: **Clinical pharmacokinetics of alfentanil, fentanyl and sufentanil. An update.** *Clin Pharmacokinet* 1996, **31:** 275-292.

55. Wada DR, Mandema JW: **Context sensitive pharmacokinetics in anesthesia: application to alfentanil [abstract].** *Pharm Res* 1994, **11:** S-424.

56. Druid H, Holmgren P: **A compilation of fatal and control concentrations of drugs in postmortem femoral blood.** *J Forensic Sci* 1997, **42:** 79-87.

57. Repetto MR, Repetto M: **Therapeutic, toxic, and lethal concentrations of 73 drugs affecting respiratory system in human fluids.** *J Toxicol Clin Toxicol* 1998, **36:** 287-293.

58. Uges DRA: TIAFT reference blood level list of therapeutic and toxic substances. [http://www.gtfch.org/cms/images/stories/Updated_TIAFT_list_202005.pdf]

59. Klotz U, Laux G: *Tranquillantien*. Stuttgart: WVG; 1996.

60. Labbate LA, Pollack MH, Otto MW, Tesar GM, Rosenbaum JF: **The relationship of alprazolam and clonazepam dose to steady-state concentration in plasma.** *J Clin Psychopharmacol* 1994, **14:** 274-276.

61. Laurijssens BE, Greenblatt DJ: **Pharmacokinetic-pharmacodynamic relationships for benzodiazepines.** *Clin Pharmacokinet* 1996, **30:** 52-76.

62. Lesser IM, Lydiard RB, Antal E, Rubin RT, Ballenger JC, DuPont R: **Alprazolam plasma concentrations and treatment response in panic disorder and agoraphobia.** *Am J Psychiatry* 1992, **149:** 1556-1562.

63. Michaud K, Augsburger M, Romain N, Giroud C, Mangin P: **Fatal overdose of tramadol and alprazolam.** *Forensic Sci Int* 1999, **105:** 185-189.

64. Flanagan RJ: **Guidelines for the interpretation of analytical toxicology results and unit of measurement conversion factors.** *Ann Clin Biochem* 1998, **35:** 261-267.

65. N.N.: **Aluminium.** *Bundesgesundhbl* 1998, **41:** 271.

66. Repetto MR, Repetto M: **Concentrations in human fluids: 101 drugs affecting the digestive system and metabolism.** *J Toxicol Clin Toxicol* 1999, **37:** 1-9.

67. Butler DR, Kuhn RJ, Chandler MH: **Pharmacokinetics of anti-infective agents in paediatric patients.** *Clin Pharmacokinet* 1994, **26:** 374-395.

68. Stork CM, Hoffman RS: **Characterization of 4-aminopyridine in overdose.** *J Toxicol Clin Toxicol* 1994, **32:** 583-587.

69. Bouillon T, Bartmus D, Schiffmann H, Gundert-Remy U: **Amiodaron zur Therapie ventrikulärer Arrhythmien bei einem Neugeborenen. Computergestützte pharmakokinetische Analyse zur Dosisfindung.** *Arzneimitteltherapie* 1994, **12:** 151-154.

70. Jürgens G, Graudal NA, Kampmann JP: **Therapeutic drug monitoring of antiarrhythmic drugs.** *Clin Pharmacokinet* 2003, **42:** 647-663.

71. Sauro SC, DeCarolis DD, Pierpont GL, Gornick CC: **Comparison of plasma concentrations for two amiodarone products.** *Ann Pharmacother* 2002, **36:** 1682-1685.

72. Mauri MC, Volonteri LS, Colasanti A, Fiorentini A, De Gaspari IF, Bareggi SR: **Clinical pharmacokinetics of atypical antipsychotics: a critical review of the relationship between plasma concentrations and clinical response.** *Clin Pharmacokinet* 2007, **46:** 359-388.

73. Boehnert MT, Lovejoy FH, Jr.: **Value of the QRS duration versus the serum drug level in predicting seizures and ventricular arrhythmias after an acute overdose of tricyclic antidepressants.** *N Engl J Med* 1985, **313:** 474-479.

74. Breyer-Pfaff U, Gaertner HJ: *Antidepressiva. Pharmakologie, therapeutischer Einsatz und Klinik der Depression*. Stuttgart: WVG; 1987.

75. el-Yazigi A, Chaleby K, Gad A, Raines DA: **Steady-state kinetics of fluoxetine and amitriptyline in patients treated with a combination of these drugs as compared with those treated with amitriptyline alone.** *J Clin Pharmacol* 1995, **35:** 17-21.

76. Furlanut M, Benetello P, Spina E: **Pharmacokinetic optimisation of tricyclic antidepressant therapy.** *Clin Pharmacokinet* 1993, **24:** 301-318.

77. Hanzlick RL: **Postmortem blood concentrations of parent tricyclic antidepressant (TCA) drugs in 11 cases of suicide.** *Am J Forensic Med Pathol* 1984, **5:** 11-13.

78. Lieberman JA, Cooper TB, Suckow RF, Steinberg H, Borenstein M, Brenner R *et al*.: **Tricyclic antidepressant and metabolite levels in chronic renal failure.** *Clin Pharmacol Ther* 1985, **37:** 301-307.

79. Lieberman JA, Cooper TB, Suckow RF, Steinberg H, Borenstein M, Brenner R *et al*.: **Tricyclic antidepressant drug and metabolite levels in chronic renal failure.** *Ann N Y Acad Sci* 1986, **463:** 304-306.

80. Linder MW, Keck PE, Jr.: **Standards of laboratory practice: antidepressant drug monitoring. National Academy of Clinical Biochemistry.** *Clin Chem* 1998, **44:** 1073-1084.

81. Miljkovic B, Pokrajac M, Timotijevic I, Varagic V: **Clinical response and plasma concentrations of amitriptyline and its metabolite-nortriptyline in depressive patients.** *Eur J Drug Metab Pharmacokinet* 1996, **21:** 251-255.

82. Preskorn SH, Fast GA: **Therapeutic drug monitoring for antidepressants: efficacy, safety, and cost effectiveness.** *J Clin Psychiatry* 1991, **52 Suppl:23-33.:** 23-33.

83. Ulrich S, Läuter J: **Comprehensive survey of the relationship between serum concentration and therapeutic effect of amitriptyline in depression.** *Clin Pharmacokinet* 2002, **41:** 853-876.

84. Kirsten R, Nelson K, Kirsten D, Heintz B: **Clinical pharmacokinetics of vasodilators. Part I.** *Clin Pharmacokinet* 1998, **34:** 457-482.

85. Koch AR, Vogelaers DP, Decruyenaere JM, Callens B, Verstraete A, Buylaert WA: **Fatal intoxication with amlodipine.** *J Toxicol Clin Toxicol* 1995, **33:** 253-256.

86. Stanek EJ, Nelson CE, DeNofrio D: **Amlodipine overdose.** *Ann Pharmacother* 1997, **31:** 853-856.

87. Musshoff F, Padosch S, Steinborn S, Madea B: **Fatal blood and tissue concentrations of more than 200 drugs.** *Forensic Sci Int* 2004, **142:** 161-210.

88. Sweetman SC: *Martindale. The complete drug reference*, 33 edn. London: Pharmaceutical Press; 2002.

89. Hellriegel ET, Arora S, Nelson M, Robertson P, Jr.: **Steady-state pharmacokinetics and tolerability of modafinil administered alone or in combination with dextroamphetamine in healthy volunteers.** *J Clin Pharmacol* 2002, **42:** 450-460.

90. Cleary JD, Hayman J, Sherwood J, Lasala GP, Piazza-Hepp T: **Amphotericin B overdose in pediatric patients with associated cardiac arrest.** *Ann Pharmacother* 1993, **27:** 715-719.

91. Hay RJ: **Recent advances in the management of fungal infections.** *Q J Med* 1987, **64:** 631-639.

92. Lipp H-P: **Amphotericin B und seine Lipidcarrier. Eine kritische Übersicht.** *Krankenhauspharmazie* 1997, **18:** 104-113.

93. Mohr JF, Hall AC, Ericsson CD, Ostrosky-Zeichner L: **Fatal amphotericin B overdose due to administration of nonlipid formulation instead of lipid formulation.** *Pharmacotherapy* 2005, **25:** 426-428.

94. Hellinger A, Wolter K, Marggraf G, Pentz R, Fritschka E: **Elimination of amrinone during continuous veno-venous haemofiltration after cardiac surgery.** *Eur J Clin Pharmacol* 1995, **48:** 57-59.

95. Kirsten R, Nelson K, Kirsten D, Heintz B: **Clinical pharmacokinetics of vasodilators. Part II.** *Clin Pharmacokinet* 1998, **35:** 9-36.

96. Paxton JW, Kim SN, Whitfield LR: **Pharmacokinetic and toxicity scaling of the antitumor agents amsacrine and CI-921, a new analogue, in mice, rats, rabbits, dogs, and humans.** *Cancer Res* 1990, **50:** 2692-2697.

97. N.N.: **Stoffmonographie und Referenzwerte für monocyclische Aminoaromaten im Urin. Stellungnahme der Kommission Human-Biomonitoring des Umweltbundesamtes.** *Bundesgesundhbl* 2011, **54:** 650-663.

98. Iwersen-Bergmann S, Schmoldt A: **Acute intoxication with aniline: detection of acetaminophen as aniline metabolite.** *Int J Legal Med* 2000, **113:** 171-174.

99. Kütting B, Goen T, Schwegler U, Fromme H, Uter W, Angerer J *et al*.: **Monoarylamines in the general population--a cross-sectional population-based study including 1004 Bavarian subjects.** *Int J Hyg Environ Health* 2009, **212:** 298-309.

100. Schulz C, Angerer J, Ewers U, Heudorf U, Wilhelm M: **Revised and new reference values for environmental pollutants in urine or blood of children in Germany derived from the German environmental survey on children 2003-2006 (GerES IV).** *Int J Hyg Environ Health* 2009, **212:** 637-647.

101. Choong E, Rudaz S, Kottelat A, Guillarme D, Veuthey JL, Eap CB: **Therapeutic drug monitoring of seven psychotropic drugs and four metabolites in human plasma by HPLC-MS.** *J Pharm Biomed Anal* 2009, **50:** 1000-1008.

102. Young MC, Shah N, Cantrell FL, Clark RF: **Risk assessment of isolated aripiprazole exposures and toxicities: a retrospective study.** *Clin Toxicol (Phila)* 2009, **47:** 580-583.

103. Duenas-Laita A, Perez-Miranda M, Gonzalez-Lopez MA, Martin-Escudero JC, Ruiz-Mambrilla M, Blanco-Varela J: **Acute arsenic poisoning.** *Lancet* 2005, **365:** 1982.

104. Oertel R, Rahn R, Kirch W: **Clinical pharmacokinetics of articaine.** *Clin Pharmacokinet* 1997, **33:** 417-425.

105. Biesalski HK: **Antioxidative Vitamine in der Prävention.** *Dt Ärztebl* 1995, **92:** A-1316.

106. Jacob RA: **Assessment of human vitamin C status.** *J Nutr* 1990, **120 Suppl 11:1480-5.:** 1480-1485.

107. Lykkesfeldt J, Prieme H, Loft S, Poulsen HE: **Effect of smoking cessation on plasma ascorbic acid concentration.** *BMJ* 1996, **313:** 91.

108. Wang S, Schram IM, Sund RB: **Determination of plasma ascorbic acid by HPLC: method and stability studies.** *Eur J Pharm Sci* 1995, **3:** 231-239.

109. Snook J, Boothman-Burrell D, Watkins J, Colin-Jones D: **Torsade de pointes ventricular tachycardia associated with astemizole overdose.** *Br J Clin Pract* 1988, **42:** 257-259.

110. **HHS Panel on Antiretroviral Guidelines for Adults and Adolescents [Guidelines for the use of antiretroviral agents in HIV-1-infected adults and adolescents].** [Department of Health & Human Services, USA] (last accessed October 14, 2011)

111. von Hentig N: **Messung von Plasmakonzentrationen antiretroviraler Arzneimittel in der HIV-Therapie.** *Dtsch Med Wochenschr* 2008, **133:** 191-195.

112. Stoschitzky K, Kahr S, Donnerer J, Schumacher M, Luha O, Maier R *et al*.: **Stereoselective increase of plasma concentrations of the enantiomers of propranolol and atenolol during exercise.** *Clin Pharmacol Ther* 1995, **57:** 543-551.

113. Spencer CM, Goa KL: **Atovaquone. A review of its pharmacological properties and therapeutic efficacy in opportunistic infections.** *Drugs* 1995, **50:** 176-196.

114. Tune L, Coyle JT: **Serum levels of anticholinergic drugs in treatment of acute extrapyramidal side effects.** *Arch Gen Psychiatry* 1980, **37:** 293-297.

115. Bahal N, Nahata MC: **The new macrolide antibiotics: azithromycin, clarithromycin, dirithromycin, and roxithromycin.** *Ann Pharmacother* 1992, **26:** 46-55.

116. Lode H: **The pharmacokinetics of azithromycin and their clinical significance.** *Eur J Clin Microbiol Infect Dis* 1991, **10:** 807-812.

117. Peters DH, Friedel HA, McTavish D: **Azithromycin. A review of its antimicrobial activity, pharmacokinetic properties and clinical efficacy.** *Drugs* 1992, **44:** 750-799.

118. Rodvold KA, Gotfried MH, Danziger LH, Servi RJ: **Intrapulmonary steady-state concentrations of clarithromycin and azithromycin in healthy adult volunteers.** *Antimicrob Agents Chemother* 1997, **41:** 1399-1402.

119. Schulz M, Peruche B: **Azithromycin, ein neues Makrolid-Antibiotikum.** *Pharm Ztg* 1994, **139:** 3346-3352.

120. Hiller JL, Benda GI, Rahatzad M, Allen JR, Culver DH, Carlson CV *et al*.: **Benzyl alcohol toxicity: impact on mortality and intraventricular hemorrhage among very low birth weight infants.** *Pediatrics* 1986, **77:** 500-506.

121. Lopez-Herce J, Bonet C, Meana A, Albajara L: **Benzyl alcohol poisoning following diazepam intravenous infusion.** *Ann Pharmacother* 1995, **29:** 632.

122. Hakamäki T, Apoil E, Arstila M, Timmer CJ, Lehtonen A: **Bepridil in the elderly. A pharmacokinetic and clinical monitoring study.** *Curr Ther Res* 1988, **44:** 752-758.

123. DRUGDEX® System. Greenwood Village, Colo: Thomson Reuters (Healthcare) Inc. Updated periodically.

124. Greenberg ER, Baron JA, Karagas MR, Stukel TA, Nierenberg DW, Stevens MM *et al*.: **Mortality associated with low plasma concentration of beta carotene and the effect of oral supplementation.** *JAMA* 1996, **275:** 699-703.

125. Berthault F, Kintz P, Tracqui A, Mangin P: **A fatal case of betaxolol poisoning.** *J Anal Toxicol* 1997, **21:** 228-231.

126. Mahler C, Verhelst J, Denis L: **Clinical pharmacokinetics of the antiandrogens and their efficacy in prostate cancer.** *Clin Pharmacokinet* 1998, **34:** 405-417.

127. Palmer RB, Alakija P, de Baca JE, Nolte KB: **Fatal brodifacoum rodenticide poisoning: autopsy and toxicologic findings.** *J Forensic Sci* 1999, **44:** 851-855.

128. Danel VC, Saviuc PF, Hardy GA, Lafond JL, Mallaret MP: **Bromide intoxication and pseudohyperchloremia.** *Ann Pharmacother* 2001, **35:** 386-387.

129. Hoizey G, Souchon PF, Trenque T, Frances C, Lamiable D, Nicolas A *et al*.: **An unusual case of methyl bromide poisoning.** *J Toxicol Clin Toxicol* 2002, **40:** 817-821.

130. Stein U, Steinecke H, Pragst F, Prügel M, Ulrich P, Gondro T: **Ionenselektive Elektroden und Mikrodestillation.** *Toxichem Krimtech* 1999, **66:** 129-141.

131. Saito T, Takeichi S, Nakajima Y, Yukawa N, Osawa M: **A case of homicidal poisoning involving several drugs.** *J Anal Toxicol* 1997, **21:** 584-586.

132. Hempel V, Lenz G: **Lokalanästhetika - Wirkungsweise, Eigenschaften, Pharmakokinetik und Toxizität.** *Anästh Intesivmed* 1982, **23:** 337-345.

133. Kastrissios H, Triggs EJ, Sinclair F, Moran P, Smithers M: **Plasma concentrations of bupivacaine after wound infiltration of an 0.5% solution after inguinal herniorrhaphy: a preliminary study.** *Eur J Clin Pharmacol* 1993, **44:** 555-557.

134. Lenderink AW, Langen MCJ, Schippers D: **Bupivacain serum levels after intra-abdominal instillation [abstract].** *Pharm World Sci* 1994, **16:** D8.

135. Elkader A, Sproule B: **Buprenorphine: clinical pharmacokinetics in the treatment of opioid dependence.** *Clin Pharmacokinet* 2005, **44:** 661-680.

136. Kuhlman JJ, Jr., Levine B, Johnson RE, Fudala PJ, Cone EJ: **Relationship of plasma buprenorphine and norbuprenorphine to withdrawal symptoms during dose induction, maintenance and withdrawal from sublingual buprenorphine.** *Addiction* 1998, **93:** 549-559.

137. Tracqui A, Kintz P, Ludes B: **Buprenorphine-related deaths among drug addicts in France: a report on 20 fatalities.** *J Anal Toxicol* 1998, **22:** 430-434.

138. Walsh SL, Preston KL, Stitzer ML, Cone EJ, Bigelow GE: **Clinical pharmacology of buprenorphine: ceiling effects at high doses.** *Clin Pharmacol Ther* 1994, **55:** 569-580.

139. Kintz P: **Deaths involving buprenorphine: a compendium of French cases.** *Forensic Sci Int* 2001, **121:** 65-69.

140. Kuhlman JJ, Jr., Lalani S, Magluilo J, Jr., Levine B, Darwin WD: **Human pharmacokinetics of intravenous, sublingual, and buccal buprenorphine.** *J Anal Toxicol* 1996, **20:** 369-378.

141. Lai SH, Yao YJ, Lo DS: **A survey of buprenorphine related deaths in Singapore.** *Forensic Sci Int* 2006, **162:** 80-86.

142. Findlay JWA, Van Wyck FJ, Smith PG, Butz RF, Hinton ML, Blum MR *et al*.: **Pharmacokinetics of bupropion, a novel antidepressant agent, following oral administration to healthy subjects.** *Eur J Clin Pharmacol* 1981, **21:** 127-135.

143. Friel PN, Logan BK, Fligner CL: **Three fatal drug overdoses involving bupropion.** *J Anal Toxicol* 1993, **17:** 436-438.

144. Holm KJ, Spencer CM: **Bupropion: a review of its use in the management of smoking cessation.** *Drugs* 2000, **59:** 1007-1024.

145. Hsyu PH, Singh A, Giargiari TD, Dunn JA, Ascher JA, Johnston JA: **Pharmacokinetics of bupropion and its metabolites in cigarette smokers versus nonsmokers.** *J Clin Pharmacol* 1997, **37:** 737-743.

146. Lai AA, Schroeder DH: **Clinical pharmacokinetics of bupropion: a review.** *J Clin Psychiatry* 1983, **44:** 82-84.

147. Posner J, Bye A, Dean K, Peck AW, Whiteman PD: **The disposition of bupropion and its metabolites in healthy male volunteers after single and multiple doses.** *Eur J Clin Pharmacol* 1985, **29:** 97-103.

148. Sweet RA, Pollock BG, Kirshner M, Wright B, Altieri LP, DeVane CL: **Pharmacokinetics of single- and multiple-dose bupropion in elderly patients with depression.** *J Clin Pharmacol* 1995, **35:** 876-884.

149. Cull G, O'Halloran S, Ilett KF: **Therapeutic drug monitoring for busulfan in plasma during conditioning chemotherapy for autologous stem cell transplantation in relapsed primary cerebral lymphoma.** *Ther Drug Monit* 2010, **32:** 333-337.

150. Juenke JM, Miller KA, McMillin GA, Johnson-Davis KL: **An automated method for supporting busulfan therapeutic drug monitoring.** *Ther Drug Monit* 2011, **33:** 315-320.

151. Malar R, Sjoo F, Rentsch K, Hassan M, Gungor T: **Therapeutic drug monitoring is essential for intravenous busulfan therapy in pediatric hematopoietic stem cell recipients.** *Pediatr Transplant* 2011, **15:** 580-588.

152. Radich JP, Gooley T, Bensinger W, Chauncey T, Clift R, Flowers M *et al*.: **HLA-matched related hematopoietic cell transplantation for chronic-phase CML using a targeted busulfan and cyclophosphamide preparative regimen.** *Blood* 2003, **102:** 31-35.

153. Slattery JT, Clift RA, Buckner CD, Radich J, Storer B, Bensinger WI *et al*.: **Marrow transplantation for chronic myeloid leukemia: the influence of plasma busulfan levels on the outcome of transplantation.** *Blood* 1997, **89:** 3055-3060.

154. Yeh RF, Pawlikowski MA, Blough DK, McDonald GB, O'Donnell PV, Rezvani A *et al*.: **Accurate Targeting of Daily Intravenous Busulfan with 8-Hour Blood Sampling in Hospitalized Adult Hematopoietic Cell Transplant Recipients.** *Biol Blood Marrow Transplant* 2011.

155. Cook DG, Peacock JL, Feyerabend C, Carey IM, Jarvis MJ, Anderson HR *et al*.: **Relation of caffeine intake and blood caffeine concentrations during pregnancy to fetal growth: prospective population based study.** *BMJ* 1996, **313:** 1358-1362.

156. Mizuno A, Uematsu T, Gotoh S, Katoh E, Nakashima M: **The measurement of caffeine concentration in scalp hair as an indicator of liver function.** *J Pharm Pharmacol* 1996, **48:** 660-664.

157. Risselmann B, Rosenbaum F, Roscher S, Schneider V: **Fatal caffeine intoxication.** *Forensic Sci Int* 1999, **103:** S49-S52.

158. Köppel C, Martens F, Schirop T, Ibe K: **Hemoperfusion in acute camphor poisoning.** *Intensive Care Med* 1988, **14:** 431-433.

159. Alderman CP: **Adverse effects of the angiotensin-converting enzyme inhibitors.** *Ann Pharmacother* 1996, **30:** 55-61.

160. Schulz M, Graefe T, Stuby K, Andresen H, Kupfermann N, Schmoldt A: **Case report: acute unintentional carbachol intoxication.** *Crit Care* 2006, **10:** R84.

161. Brodie MJ, Dichter MA: **Antiepileptic drugs.** *N Engl J Med* 1996, **334:** 168-175.

162. Collins DM, Gidal BE, Pitterle ME: **Potential interaction between carbamazepine and loxapine: case report and retrospective review.** *Ann Pharmacother* 1993, **27:** 1180-1187.

163. Elmquist WF, Riad LE, Leppik IE, Sawchuk RJ: **The relationship between urine and plasma concentrations of carbamazepine: implications for therapeutic drug monitoring.** *Pharm Res* 1991, **8:** 282-284.

164. French J: **The long-term therapeutic management of epilepsy.** *Ann Intern Med* 1994, **120:** 411-422.

165. Kale PB, Thomson PA, Provenzano R, Higgins MJ: **Evaluation of plasmapheresis in the treatment of an acute overdose of carbamazepine.** *Ann Pharmacother* 1993, **27:** 866-870.

166. Liu H, Delgado MR: **Therapeutic drug concentration monitoring using saliva samples. Focus on anticonvulsants.** *Clin Pharmacokinet* 1999, **36:** 453-470.

167. Duck BJ, Woolias M: **Reversed-phase high performance liquid chromatographic determination of carbaryl in postmortem specimens.** *J Anal Toxicol* 1985, **9:** 177-179.

168. Stockis A, Deroubaix X, Jeanbaptiste B, Lins R, Allemon AM, Laufen H: **Relative bioavailability of carbinoxamine and phenylephrine from a retard capsule after single and repeated dose administration in healthy subjects.** *Arzneimittelforschung* 1995, **45:** 1009-1012.

169. Schmoldt A, Schulz M, Frese JH: **Klinik und Therapie einer Intoxikation mit Tetrachlorkohlenstoff.** In *Gerichtsmedizin. Festschrift für Wilhelm Holczabek*. Edited by Bauer G. Wien: Franz Deuticke; 1988:529-531.

170. Goldermann L, Gellert J, Teschke R: **Quantitative assessment of carbon tetrachloride levels in human blood by head-space gas chromatography: application in a case of suicidal carbon tetrachloride intoxication.** *Intensive Care Med* 1983, **9:** 131-135.

171. Mathieson PW, Williams G, MacSweeney JE: **Survival after massive ingestion of carbon tetrachloride treated by intravenous infusion of acetylcysteine.** *Hum Toxicol* 1985, **4:** 627-631.

172. Ruprah M, Mant TG, Flanagan RJ: **Acute carbon tetrachloride poisoning in 19 patients: implications for diagnosis and treatment.** *Lancet* 1985, **1:** 1027-1029.

173. Tombolini A, Cingolani M: **Fatal accidental ingestion of carbon tetrachloride: a postmortem distribution study.** *J Forensic Sci* 1996, **41:** 166-168.

174. Danziger LH, Piscitelli SC, Occhipinti DJ, Resnick DJ, Rodvold KA: **Steady-state pharmacokinetics of cefoperazone and sulbactam in patients with acute appendicitis.** *Ann Pharmacother* 1994, **28:** 703-707.

175. Schulz M, Schmoldt A: **Konzentrationen von Cefotiam im Kolongewebe und Plasma nach Applikation zur perioperativen Antibiotikaprophylaxe.** *ZAC Zeitschr antimikr antineopl Chemother* 1992, **10:** 33-37.

176. Lorenz R, Lehn N, Born P, Herrmann M, Neuhaus H: **Antibiotische Prophylaxe mit Cefuroxim bei endoskopischen Eingriffen an den Gallenwegen.** *Dtsch Med Wochenschr* 1996, **121:** 223-230.

177. Pass SE, Miyagawa CI, Healy DP, Ivey TD: **Serum concentrations of cefuroxime after continuous infusion in coronary bypass graft patients.** *Ann Pharmacother* 2001, **35:** 409-413.

178. Koren G: **Therapeutic drug monitoring principles in the neonate. National Academy of CLinical Biochemistry.** *Clin Chem* 1997, **43:** 222-227.

179. Bailey DN: **Blood concentrations and clinical findings following overdose of chlordiazepoxide alone and chlordiazepoxide plus ethanol.** *J Toxicol Clin Toxicol* 1984, **22:** 433-446.

180. Maxa JL, Ogu CC, Adeeko MA, Swaner TG: **Continuous-infusion flumazenil in the management of chlordiazepoxide toxicity.** *Pharmacotherapy* 2003, **23:** 1513-1516.

181. Köppel C, Kristinsson J, Wagemann A, Tenczer J, Martens F: **Chlormezanone plasma and blood levels in patients after single and repeated oral doses and after suicidal drug overdose.** *Eur J Drug Metab Pharmacokinet* 1991, **16:** 43-47.

182. Dell'Aglio DM, Sutter ME, Schwartz MD, Koch DD, Algren DA, Morgan BW: **Acute chloroform ingestion successfully treated with intravenously administered N-acetylcysteine.** *J Med Toxicol* 2010, **6:** 143-146.

183. Croes K, Augstijns P, Sabbe M, Desmet K, Verbeke N: **Diazepam treatment in chloroquine intoxication: a case report [abstract].** *Pharm Weekbl Sci* 1992, **14:** D9.

184. Javaid JI: **Clinical pharmacokinetics of antipsychotics.** *J Clin Pharmacol* 1994, **34:** 286-295.

185. Milton GV, Jann MW: **Emergency treatment of psychotic symptoms. Pharmacokinetic considerations for antipsychotic drugs.** *Clin Pharmacokinet* 1995, **28:** 494-504.

186. Eddleston M, Eyer P, Worek F, Mohamed F, Senarathna L, von ML *et al*.: **Differences between organophosphorus insecticides in human self-poisoning: a prospective cohort study.** *Lancet* 2005, **366:** 1452-1459.

187. Vaughan Williams EM: **Classifying antiarrhythmic actions: by facts or speculation.** *J Clin Pharmacol* 1992, **32:** 964-977.

188. Dumont RJ, Ensom MH: **Methods for clinical monitoring of cyclosporin in transplant patients.** *Clin Pharmacokinet* 2000, **38:** 427-447.

189. Horton RC, Bonser RS: **Interaction between cyclosporin and fluoxetine.** *BMJ* 1995, **311:** 422.

190. Lindholm A: **Cyclosporine A: clinical experience and therapeutic drug monitoring.** *Ther Drug Monit* 1995, **17:** 631-637.

191. Lindholm A, Sawe J: **Pharmacokinetics and therapeutic drug monitoring of immunosuppressants.** *Ther Drug Monit* 1995, **17:** 570-573.

192. Oellerich M, Armstrong VW, Kahan B, Shaw L, Holt DW, Yatscoff R *et al*.: **Lake Louise Consensus Conference on cyclosporin monitoring in organ transplantation: report of the consensus panel.** *Ther Drug Monit* 1995, **17:** 642-654.

193. Tonkin AL, Bochner F: **Therapeutic drug monitoring and patient outcome. A review of the issues.** *Clin Pharmacokinet* 1994, **27:** 169-174.

194. Gugler R, Fuchs G, Dieckmann M, Somogyi AA: **Cimetidine plasma concentration-response relationships.** *Clin Pharmacol Ther* 1981, **29:** 744-748.

195. Shinn AF: **Clinical relevance of cimetidine drug interactions.** *Drug Saf* 1992, **7:** 245-267.

196. Cohen H, Francisco DH: **Twelve-gram overdose of ciprofloxacin with mild symptomatology.** *Ann Pharmacother* 1994, **28:** 805-806.

197. Davis JD, Aarons L, Houston JB: **Relationship between enoxacin and ciprofloxacin plasma concentrations and theophylline disposition.** *Pharm Res* 1994, **11:** 1424-1428.

198. Schentag JJ, Nix DE, Adelman MH: **Mathematical examination of dual individualization principles (I): Relationships between AUC above MIC and area under the inhibitory curve for cefmenoxime, ciprofloxacin, and tobramycin.** *DICP* 1991, **25:** 1050-1057.

199. Staß H, Peltola H, Kuhlmann J, Rahm V: **Single dose and steady state pharmacokinetics of ciprofloxacin (CIP) in pediatric patients following administration of a new oral suspension (10 mg/kg tid) [abstract].** *Naunyn-Schmiedeberg's Arch Pharmacol* 1996, **353:** R153.

200. Friberg LE, Isbister GK, Hackett LP, Duffull SB: **The population pharmacokinetics of citalopram after deliberate self-poisoning: a Bayesian approach.** *J Pharmacokinet Pharmacodyn* 2005, **32:** 571-605.

201. Gutierrez M, Abramowitz W: **Steady-state pharmacokinetics of citalopram in young and elderly subjects.** *Pharmacotherapy* 2000, **20:** 1441-1447.

202. van Harten J: **Clinical pharmacokinetics of selective serotonin reuptake inhibitors.** *Clin Pharmacokinet* 1993, **24:** 203-220.

203. Jaehde U, Sorgel F, Reiter A, Sigl G, Naber KG, Schunack W: **Effect of probenecid on the distribution and elimination of ciprofloxacin in humans.** *Clin Pharmacol Ther* 1995, **58:** 532-541.

204. Öström M, Eriksson A, Thorson J, Spigset O: **Fatal overdose with citalopram.** *Lancet* 1996, **348:** 339-340.

205. Overo KF: **Preliminary studies of the kinetics of citalopram in man.** *Eur J Clin Pharmacol* 1978, **14:** 69-73.

206. Overo KF: **Kinetics of citalopram in man; plasma levels in patients.** *Prog Neuropsychopharmacol Biol Psychiatry* 1982, **6:** 311-318.

207. Overo KF, Toft B, Christophersen L, Gylding-Sabroe JP: **Kinetics of citalopram in elderly patients.** *Psychopharmacology (Berl )* 1985, **86:** 253-257.

208. Beutler E: **Cladribine (2-chlorodeoxyadenosine).** *Lancet* 1992, **340:** 952-956.

209. Johnson SA: **Clinical pharmacokinetics of nucleoside analogues: focus on haematological malignancies.** *Clin Pharmacokinet* 2000, **39:** 5-26.

210. Kath R, Knauf WU, Mitrou PS, Rummel M, Höffken K, Peters HD: **Cladribin (2-CdA). Pharmakologisches Profil und klinische Anwendung.** *Onkologe* 1995, **1:** 626.

211. Boruchoff SE, Sturgill MG, Grasing KW, Seibold JR, McCrea J, Winchell GA *et al*.: **The steady-state disposition of indinavir is not altered by the concomitant administration of clarithromycin.** *Clin Pharmacol Ther* 2000, **67:** 351-359.

212. Rodvold KA: **Clinical pharmacokinetics of clarithromycin.** *Clin Pharmacokinet* 1999, **37:** 385-398.

213. Schulz J: **Clarithromycin - ein neues Makrolid-Antibiotikum.** *Pharm Ztg* 1992, **137:** 1626-1631.

214. Breccia A, Ferri E, Girotti S, Bignanmini AA, Budini RA: **High performance liquid chromatography and capillary gas chromatography-mass spectrometry determination of clemastine in serum: plasma kinetics after dermatologic application.** *Curr Ther Res* 1991, **49:** 622-626.

215. Knapp J, Boknik P, Gumbinger HG, Linck B, Luss H, Muller FU *et al*.: **Quantitation of clobazam in human plasma using high-performance liquid chromatography.** *J Chromatogr Sci* 1999, **37:** 145-149.

216. Klug E, Schneider V: **Vergiftungen durch Clomethiazol.** *Z Rechtsmed* 1984, **93:** 89-94.

217. Ulrich S, Danos P, Baumann B, Muller D, Lehmann D, Treuheit TO *et al*.: **Serum concentration of chlormethiazole and therapeutic effect in acute alcohol withdrawal syndrome: an open clinical trial.** *Ther Drug Monit* 2002, **24:** 446-454.

218. Balant-Gorgia AE, Gex-Fabry M, Balant LP: **Clinical pharmacokinetics of clomipramine.** *Clin Pharmacokinet* 1991, **20:** 447-462.

219. Dale O, Hole A: **Biphasic time-course of serum concentrations of clomipramine and desmethylclomipramine after a near-fatal overdose.** *Vet Hum Toxicol* 1994, **36:** 309-310.

220. Faravelli C, Ballerini A, Ambonetti A, Broadhurst AD, Das M: **Plasma levels and clinical response during treatment with clomipramine.** *J Affect Disord* 1984, **6:** 95-107.

221. Gex-Fabry M, Balant-Gorgia AE, Balant LP: **Clomipramine concentration as a predictor of delayed response: a naturalistic study.** *Eur J Clin Pharmacol* 1999, **54:** 895-902.

222. Kuss HJ, Jungkunz G: **Nonlinear pharmacokinetics of chlorimipramine after infusion and oral administration in patients.** *Prog Neuropsychopharmacol Biol Psychiatry* 1986, **10:** 739-748.

223. Waade RB, Molden E, Refsum H, Hermann M: **Serum concentrations of antidepressants in the elderly.** *Ther Drug Monit* 2012, **34:** 25-30.

224. Rey E, Treluyer JM, Pons G: **Pharmacokinetic optimization of benzodiazepine therapy for acute seizures. Focus on delivery routes.** *Clin Pharmacokinet* 1999, **36:** 409-424.

225. Erickson SJ, Duncan A: **Clonidine poisoning--an emerging problem: epidemiology, clinical features, management and preventative strategies.** *J Paediatr Child Health* 1998, **34:** 280-282.

226. Nichols MH, King WD, James LP: **Clonidine poisoning in Jefferson County, Alabama.** *Ann Emerg Med* 1997, **29:** 511-517.

227. Raber JH, Shinar C, Finkelstein S: **Clonidine patch ingestion in an adult.** *Ann Pharmacother* 1993, **27:** 719-722.

228. Couchman L, Morgan PE, Spencer EP, Flanagan RJ: **Plasma clozapine, norclozapine, and the clozapine:norclozapine ratio in relation to prescribed dose and other factors: data from a therapeutic drug monitoring service, 1993-2007.** *Ther Drug Monit* 2010, **32:** 438-447.

229. Dahl SG: **Pharmacokinetics of antipsychotic drugs in man.** *Acta Psychiatr Scand Suppl* 1990, **358:37-40.:** 37-40.

230. Dettling M, Sachse C, Brockmoller J, Schley J, Muller-Oerlinghausen B, Pickersgill I *et al*.: **Long-term therapeutic drug monitoring of clozapine and metabolites in psychiatric in- and outpatients.** *Psychopharmacology (Berl )* 2000, **152:** 80-86.

231. Fleischhaker C, Schulz E, Clement H-W, Krieg C, Remschmidt H: **Therapeutisches Drug-Monitoring von Clozapin bei Kindern, Jugendlichen und Erwachsenen mit einer schizophrenen Psychose.** *Psychopharmakotherapie* 1999, **6:** 102-105.

232. Guitton C, Kinowski JM, Abbar M, Chabrand P, Bressolle F: **Clozapine and metabolite concentrations during treatment of patients with chronic schizophrenia.** *J Clin Pharmacol* 1999, **39:** 721-728.

233. Ismail Z, Wessels AM, Uchida H, Ng W, Mamo DC, Rajji TK *et al*.: **Age and Sex Impact Clozapine Plasma Concentrations in Inpatients and Outpatients With Schizophrenia.** *Am J Geriatr Psychiatry* 2011.

234. Mahoney MC, Connolly BF, Smith CM: **A clozapine overdose with markedly elevated serum levels.** *J Clin Pharmacol* 1999, **39:** 97-100.

235. Schulte P: **What is an adequate trial with clozapine?: therapeutic drug monitoring and time to response in treatment-refractory schizophrenia.** *Clin Pharmacokinet* 2003, **42:** 607-618.

236. Spina E, Avenoso A, Facciola G, Scordo MG, Ancione M, Madia AG *et al*.: **Relationship between plasma concentrations of clozapine and norclozapine and therapeutic response in patients with schizophrenia resistant to conventional neuroleptics.** *Psychopharmacology (Berl )* 2000, **148:** 83-89.

237. Ulrich S, Wolf R, Staedt J: **Serum level of clozapine and relapse.** *Ther Drug Monit* 2003, **25:** 252-255.

238. Van der Zwaag C, McGee M, McEvoy JP, Freudenreich O, Wilson WH, Cooper TB: **Response of patients with treatment-refractory schizophrenia to clozapine within three serum level ranges.** *Am J Psychiatry* 1996, **153:** 1579-1584.

239. Wohlfarth A, Toepfner N, Hermanns-Clausen M, Auwarter V: **Sensitive quantification of clozapine and its main metabolites norclozapine and clozapine-N-oxide in serum and urine using LC-MS/MS after simple liquid-liquid extraction work-up.** *Anal Bioanal Chem* 2011, **400:** 737-746.

240. Zaleon CR, Guthrie SK: **Antipsychotic drug use in older adults.** *Am J Hosp Pharm* 1994, **51:** 2917-2943.

241. Buechler KF, Moi S, Noar B, McGrath D, Villela J, Clancy M *et al*.: **Simultaneous detection of seven drugs of abuse by the Triage panel for drugs of abuse.** *Clin Chem* 1992, **38:** 1678-1684.

242. Perez-Reyes M, Jeffcoat AR: **Ethanol/cocaine interaction: cocaine and cocaethylene plasma concentrations and their relationship to subjective and cardiovascular effects.** *Life Sci* 1992, **51:** 553-563.

243. Skeith KJ, Brocks DR: **Pharmacokinetic optimisation of the treatment of osteoarthritis.** *Clin Pharmacokinet* 1994, **26:** 233-242.

244. Meyer MR, Maurer HH: **Absorption, distribution, metabolism and excretion pharmacogenomics of drugs of abuse.** *Pharmacogenomics* 2011, **12:** 215-233.

245. Baud FJ, Sabouraud A, Vicaut E, Taboulet P, Lang J, Bismuth C *et al*.: **Brief report: treatment of severe colchicine overdose with colchicine-specific Fab fragments.** *N Engl J Med* 1995, **332:** 642-645.

246. Brvar M, Ploj T, Kozelj G, Mozina M, Noc M, Bunc M: **Case report: fatal poisoning with Colchicum autumnale.** *Crit Care* 2004, **8:** R56-R59.

247. Peters FT, Beyer J, Ewald AH: **Colchicine poisoning after mix-up of Ramsons (Allium ursinum L.) and meadow saffron (Colchicum autumnale L.).** *Toxichem Krimtech* 2004, **71:** 156-160.

248. Rochdi M, Sabouraud A, Baud FJ, Bismuth C, Scherrmann JM: **Toxicokinetics of colchicine in humans: analysis of tissue, plasma and urine data in ten cases.** *Hum Exp Toxicol* 1992, **11:** 510-516.

249. Grobosch T, Angelow B, Lampe D: **Akute Intoxikation mit Coumatetralyl. Simultane Bestimmung von 5 Superwarfarinen und 5 weiteren Vitamin K-Antagonisten in Humanserum mittels LC-ESI-MS.** *Toxichem Krimtech* 2005, **72:** 46-55.

250. Baud FJ, Borron SW, Bavoux E, Astier A, Hoffman JR: **Relation between plasma lactate and blood cyanide concentrations in acute cyanide poisoning.** *BMJ* 1996, **312:** 26-27.

251. Gracia R, Shepherd G: **Cyanide poisoning and its treatment.** *Pharmacotherapy* 2004, **24:** 1358-1365.

252. Hall AH, Rumack BH: **Clinical toxicology of cyanide.** *Ann Emerg Med* 1986, **15:** 1067-1074.

253. Houeto P, Hoffman JR, Imbert M, Levillain P, Baud FJ: **Relation of blood cyanide to plasma cyanocobalamin concentration after a fixed dose of hydroxocobalamin in cyanide poisoning.** *Lancet* 1995, **346:** 605-608.

254. Rindone JP, Sloane EP: **Cyanide toxicity from sodium nitroprusside: risks and management.** *Ann Pharmacother* 1992, **26:** 515-519.

255. Salkowski AA, Penney DG: **Cyanide poisoning in animals and humans: a review.** *Vet Hum Toxicol* 1994, **36:** 455-466.

256. Strehl E: **Cyanid-Intoxikationen. Hydroxycobalamin bereichert das Antidotarium.** *Krankenhauspharmazie* 2000, **21:** 293-297.

257. Winchell GA, King JD, Chavez-Eng CM, Constanzer ML, Korn SH: **Cyclobenzaprine pharmacokinetics, including the effects of age, gender, and hepatic insufficiency.** *J Clin Pharmacol* 2002, **42:** 61-69.

258. Belldina EB, Huang MY, Schneider JA, Brundage RC, Tracy TS: **Steady-state pharmacokinetics and pharmacodynamics of cysteamine bitartrate in paediatric nephropathic cystinosis patients.** *Br J Clin Pharmacol* 2003, **56:** 520-525.

259. USP DI® Volume I: *Drug Information for the Health Care Professional.* 26t^h^ edition. Greenwood Village, CO, USA: Thomson Reuters (Healthcare) Inc.; 2006.

260. Kraemer T, Paul LD, Jochum C, Maurer HH: **Acute poisoning with dapsone - a case report.** *Toxichem Krimtech* 2002, **69:** 80-85.

261. Piscitelli SC, Occhipinti DJ, Danziger LH, Hill C, West DP, Fischer JH: **Therapeutic monitoring and pharmacist intervention in a Hansen's disease clinic.** *Ann Pharmacother* 1993, **27:** 1526-1531.

262. Fabbiani M, Bracciale L, Ragazzoni E, Santangelo R, Cattani P, Di GS *et al*.: **Relationship between antiretroviral plasma concentration and emergence of HIV-1 resistance mutations at treatment failure.** *Infection* 2011, **39:** 563-569.

263. Burke MJ, Harvey AT, Preskorn SH: **Pharmacokinetics of the newer antidepressants.** *Am J Med* 1996, **100:** 119-121.

264. von Moltke LL, Greenblatt DJ, Shader RI: **Clinical pharmacokinetics of antidepressants in the elderly. Therapeutic implications.** *Clin Pharmacokinet* 1993, **24:** 141-160.

265. Richter O, Ern B, Reinhardt D, Becker B: **Pharmacokinetics of dexamethasone in children.** *Pediatr Pharmacol (New York )* 1983, **3:** 329-337.

266. Gunn VL, Taha SH, Liebelt EL, Serwint JR: **Toxicity of over-the-counter cough and cold medications.** *Pediatrics* 2001, **108:** E52.

267. Hanzlick R: **National Association of Medical Examiners Pediatric Toxicology (PedTox) Registry Report 3. Case submission summary and data for acetaminophen, benzene, carboxyhemoglobin, dextromethorphan, ethanol, phenobarbital, and pseudoephedrine.** *Am J Forensic Med Pathol* 1995, **16:** 270-277.

268. Härtter S, Baier D, Dingemanse J, Ziegler G, Hiemke C: **Steady state pharmacokinetics of dextromethorphan [abstract].** *Naunyn-Schmiedeberg's Arch Pharmacol (Suppl )* 1996, **353:** R154.

269. Marinetti L, Lehman L, Casto B, Harshbarger K, Kubiczek P, Davis J: **Over-the-counter cold medications-postmortem findings in infants and the relationship to cause of death.** *J Anal Toxicol* 2005, **29:** 738-743.

270. Repetto MR, Repetto M: **Habitual, toxic, and lethal concentrations of 103 drugs of abuse in humans.** *J Toxicol Clin Toxicol* 1997, **35:** 1-9.

271. Kintz P, Tracqui A, Mangin P, Lugnier AA, Chaumont AJ: **Fatal intoxication by dextromoramide: a report on two cases.** *J Anal Toxicol* 1989, **13:** 238-239.

272. Ufkes JG, de Vos JW, van Brussel GH: **Determination and pharmacokinetics of dextromoramide in methadone maintenance therapy.** *Pharm World Sci* 1998, **20:** 83-87.

273. Iwersen-Bergmann S, Toennes SW, Schmidt K, Köhler W, Zokai A, Kauert GF: **Bewertung von Morphinspiegeln bei fraglich Herointoten - immer noch eine Herausforderung [abstract].** *Rechtsmedizin* 2007, **17:** 259.

274. Teske J, Weller JP, Tröger HD, Koal T, Kaever V, Breyer R *et al*.: **Blutspiegel von Heroinfolgeprodukten bei hochdosierter Heroinapplikation [abstract].** *Rechtsmedizin* 2004, **14:** 315.

275. Halbsguth U, Rentsch KM, Eich-Hochli D, Diterich I, Fattinger K: **Oral diacetylmorphine (heroin) yields greater morphine bioavailability than oral morphine: bioavailability related to dosage and prior opioid exposure.** *Br J Clin Pharmacol* 2008, **66:** 781-791.

276. Perger L, Rentsch KM, Kullak-Ublick GA, Verotta D, Fattinger K: **Oral heroin in opioid-dependent patients: pharmacokinetic comparison of immediate and extended release tablets.** *Eur J Pharm Sci* 2009, **36:** 421-432.

277. Girardin F, Rentsch KM, Schwab MA, Maggiorini M, Pauli-Magnus C, Kullak-Ublick GA *et al*.: **Pharmacokinetics of high doses of intramuscular and oral heroin in narcotic addicts.** *Clin Pharmacol Ther* 2003, **74:** 341-352.

278. Kidd S, Brennan S, Stephen R, Minns R, Beattie T: **Comparison of morphine concentration-time profiles following intravenous and intranasal diamorphine in children.** *Arch Dis Child* 2009, **94:** 974-978.

279. Rentsch KM, Kullak-Ublick GA, Reichel C, Meier PJ, Fattinger K: **Arterial and venous pharmacokinetics of intravenous heroin in subjects who are addicted to narcotics.** *Clin Pharmacol Ther* 2001, **70:** 237-246.

280. Rook EJ, Huitema AD, van den Brink W, van Ree JM, Beijnen JH: **Population pharmacokinetics of heroin and its major metabolites.** *Clin Pharmacokinet* 2006, **45:** 401-417.

281. Rook EJ, van Ree JM, van den Brink W, Hillebrand MJ, Huitema AD, Hendriks VM *et al*.: **Pharmacokinetics and pharmacodynamics of high doses of pharmaceutically prepared heroin, by intravenous or by inhalation route in opioid-dependent patients.** *Basic Clin Pharmacol Toxicol* 2006, **98:** 86-96.

282. Bever CT, Jr., Leslie J, Camenga DL, Panitch HS, Johnson KP: **Preliminary trial of 3,4-diaminopyridine in patients with multiple sclerosis.** *Ann Neurol* 1990, **27:** 421-427.

283. Divoll M, Greenblatt DJ, Lacasse Y, Shader RI: **Benzodiazepine overdosage: plasma concentrations and clinical outcome.** *Psychopharmacology (Berl )* 1981, **73:** 381-383.

284. Friedman H, Greenblatt DJ, Peters GR, Metzler CM, Charlton MD, Harmatz JS *et al*.: **Pharmacokinetics and pharmacodynamics of oral diazepam: effect of dose, plasma concentration, and time.** *Clin Pharmacol Ther* 1992, **52:** 139-150.

285. Klotz U, Avant GR, Hoyumpa A, Schenker S, Wilkinson GR: **The effects of age and liver disease on the disposition and elimination of diazepam in adult man.** *J Clin Invest* 1975, **55:** 347-359.

286. Traeger SM, Haug MT, III: **Reduction of diazepam serum half life and reversal of coma by activated charcoal in a patient with severe liver disease.** *J Toxicol Clin Toxicol* 1986, **24:** 329-337.

287. Davies NM, Anderson KE: **Clinical pharmacokinetics of diclofenac. Therapeutic insights and pitfalls.** *Clin Pharmacokinet* 1997, **33:** 184-213.

288. Fowler PD, Dawes PT, John VA, Shotton PA: **Plasma and synovial fluid concentrations of diclofenac sodium and its hydroxylated metabolites during once-daily administration of a 100 mg slow-release formulation.** *Eur J Clin Pharmacol* 1986, **31:** 469-472.

289. Fowler PD, Shadforth MF, Crook PR, John VA: **Plasma and synovial fluid concentrations of diclofenac sodium and its major hydroxylated metabolites during long-term treatment of rheumatoid arthritis.** *Eur J Clin Pharmacol* 1983, **25:** 389-394.

290. Burger DM, Meenhorst PL, Beijnen JH: **Concise overview of the clinical pharmacokinetics of dideoxynucleoside antiretroviral agents.** *Pharm World Sci* 1995, **17:** 25-30.

291. Burger DM, Meenhorst PL, ten Napel CHH, Mulder JW, Henrichs JH, Frissen PHJ *et al*.: **Limited sampling models for the antiretroviral agent didanosine.** *J Pharm Sci* 1995, **3:** 7-13.

292. Bolla S, Boinpally RR, Poondru S, Devaraj R, Jasti BR: **Pharmacokinetics of diethylcarbamazine after single oral dose at two different times of day in human subjects.** *J Clin Pharmacol* 2002, **42:** 327-331.

293. White S, Wong SH: **Standards of laboratory practice: analgesic drug monitoring. National Academy of Clinical Biochemistry.** *Clin Chem* 1998, **44:** 1110-1123.

294. Dollery C: *Therapeutic Drugs*. Edinburgh: Churchill Livingstone; 1991.

295. Gschwantler M, Gulz W, Brownstone E, Feichtenschlager T, Pulgram T, Schrutka-Kolbl C *et al*.: **Digitoxin-induzierte Thrombozytopenie.** *Wien Klin Wochenschr* 1993, **105:** 500-502.

296. Daldrup T, Pier S: **Tödliche Vergiftung durch Orphenadrin/Diphenhydramin.** *Toxichem Krimtech* 1994, **61:** 9.

297. Isabelle C, Warner A: **Long-term heavy use of diphenhydramine without anticholinergic delirium.** *Am J Health Syst Pharm* 1999, **56:** 555-557.

298. Oikkonen M, Karkela J, Seppala T: **CSF concentrations and clinical effects following intravenous dixyrazine premedication.** *Eur J Clin Pharmacol* 1995, **47:** 445-447.

299. Barone JA: **Domperidone: a peripherally acting dopamine2-receptor antagonist.** *Ann Pharmacother* 1999, **33:** 429-440.

300. Heykants J, Hendriks R, Meuldermans W, Michiels M, Scheygrond H, Reyntjens H: **On the pharmacokinetics of domperidone in animals and man. IV. The pharmacokinetics of intravenous domperidone and its bioavailability in man following intramuscular, oral and rectal administration.** *Eur J Drug Metab Pharmacokinet* 1981, **6:** 61-70.

301. Huang YC, Colaizzi JL, Bierman RH, Woestenborghs R, Heykants JJ: **Pharmacokinetics and dose proportionality of domperidone in healthy volunteers.** *J Clin Pharmacol* 1986, **26:** 628-632.

302. Michiels M, Hendriks R, Heykants J: **On the pharmacokinetics of domperidone in animals and man II. Tissue distribution, placental and milk transfer of domperidone in the Wistar rat.** *Eur J Drug Metab Pharmacokinet* 1981, **6:** 37-48.

303. Imbimbo BP: **Pharmacodynamic-tolerability relationships of cholinesterase inhibitors for Alzheimer's disease.** *CNS Drugs* 2001, **15:** 375-390.

304. Rogers SL, Friedhoff LT: **The efficacy and safety of donepezil in patients with Alzheimer's disease: results of a US Multicentre, Randomized, Double-Blind, Placebo-Controlled Trial. The Donepezil Study Group.** *Dementia* 1996, **7:** 293-303.

305. Keller T, Schneider A, Tutsch-Bauer E: **Fatal intoxication due to dothiepin.** *Forensic Sci Int* 2000, **109:** 159-166.

306. Schulz M, Schmoldt A: **Successful physostigmine treatment of acute dothiepin intoxication.** *Pharmazie* 1994, **49:** 614.

307. Barbe F, Hansen C, Badonnel Y, Legagneur H, Vert P, Boutroy MJ: **Severe side effects and drug plasma concentrations in preterm infants treated with doxapram.** *Ther Drug Monit* 1999, **21:** 547-552.

308. Apple FS: **Postmortem tricyclic antidepressant concentrations: assessing cause of death using parent drug to metabolite ratio.** *J Anal Toxicol* 1989, **13:** 197-198.

309. Ereshefsky L, Tran-Johnson T, Davis CM, LeRoy A: **Pharmacokinetic factors affecting antidepressant drug clearance and clinical effect: evaluation of doxepin and imipramine--new data and review.** *Clin Chem* 1988, **34:** 863-880.

310. Kretzschmar M: **Intoxikationen mit schlaffördernden Mitteln.** *Z Arztl Fortbild Qual sich* 2001, **95:** 45-49.

311. Bockholdt B, Klug E, Schneider V: **Suicide through doxylamine poisoning.** *Forensic Sci Int* 2001, **119:** 138-140.

312. Johansson E, Agurell S, Hollister LE, Halldin MM: **Prolonged apparent half-life of delta 1-tetrahydrocannabinol in plasma of chronic marijuana users.** *J Pharm Pharmacol* 1988, **40:** 374-375.

313. Ohlsson A, Lindgren JE, Wahlen A, Agurell S, Hollister LE, Gillespie HK: **Plasma delta-9 tetrahydrocannabinol concentrations and clinical effects after oral and intravenous administration and smoking.** *Clin Pharmacol Ther* 1980, **28:** 409-416.

314. Sawyer CA, Baker AB, Ramzan I, Regaglia F: **Droperidol elimination after cardiopulmonary bypass surgery.** *J Clin Pharmacol* 1998, **38:** 160-165.

315. Committee for Medicinal Products for Human Use (CHMP). **Xigris (drotrecogin alfa) EPAR - Product information.** [European Medicines Agency] (last accessed April 22, 2010)

316. Aquilonius SM, Hartvig P: **Clinical pharmacokinetics of cholinesterase inhibitors.** *Clin Pharmacokinet* 1986, **11:** 236-249.

317. Iseman MD: **Treatment of multidrug-resistant tuberculosis.** *N Engl J Med* 1993, **329:** 784-791.

318. Brent J, McMartin K, Phillips S, Burkhart KK, Donovan JW, Wells M *et al*.: **Fomepizole for the treatment of ethylene glycol poisoning. Methylpyrazole for Toxic Alcohols Study Group.** *N Engl J Med* 1999, **340:** 832-838.

319. Fraser AD: **Clinical toxicologic implications of ethylene glycol and glycolic acid poisoning.** *Ther Drug Monit* 2002, **24:** 232-238.

320. Divanon F, Leroyer R, Leprince MC, Riby JP, Collet C: **A propos d'une intoxication par l'éthylène glycol.** *J Pharm Clin* 1997, **16:** 177-182.

321. Hantson P, Vanbinst R, Mahieu P: **Determination of ethylene glycol tissue content after fatal oral poisoning and pathologic findings.** *Am J Forensic Med Pathol* 2002, **23:** 159-161.

322. Hoffmann U, Abel P, Neurath H: **Acute ethylene glycol poisoning after intentional ingestion.** *Toxichem Krimtech* 2008, **75:** 130-133.

323. Leikin JB, Toerne T, Burda A, McAllister K, Erickson T: **Summertime cluster of intentional ethylene glycol ingestions.** *JAMA* 1997, **278:** 1406.

324. Porter WH, Rutter PW, Bush BA, Pappas AA, Dunnington JE: **Ethylene glycol toxicity: the role of serum glycolic acid in hemodialysis.** *J Toxicol Clin Toxicol* 2001, **39:** 607-615.

325. Reddy NJ, Lewis LD, Gardner TB, Osterling W, Eskey CJ, Nierenberg DW: **Two cases of rapid onset Parkinson's syndrome following toxic ingestion of ethylene glycol and methanol.** *Clin Pharmacol Ther* 2007, **81:** 114-121.

326. Sivilotti ML, Burns MJ, McMartin KE, Brent J: **Toxicokinetics of ethylene glycol during fomepizole therapy: implications for management. For the Methylpyrazole for Toxic Alcohols Study Group.** *Ann Emerg Med* 2000, **36:** 114-125.

327. Wildsmith JA, Tucker GT, Cooper S, Scott DB, Covino BG: **Plasma concentrations of local anaesthetics after interscalene brachial plexus block.** *Br J Anaesth* 1977, **49:** 461-466.

328. Boni J, Korth-Bradley J, McGoldrick K, Appel A, Cooper S: **Pharmacokinetic and pharmacodynamic action of etodolac in patients after oral surgery.** *J Clin Pharmacol* 1999, **39:** 729-737.

329. Kirchner GI, Meier-Wiedenbach I, Manns MP: **Clinical pharmacokinetics of everolimus.** *Clin Pharmacokinet* 2004, **43:** 83-95.

330. Kovarik JM, Kaplan B, Tedesco SH, Kahan BD, Dantal J, Vitko S *et al*.: **Exposure-response relationships for everolimus in de novo kidney transplantation: defining a therapeutic range.** *Transplantation* 2002, **73:** 920-925.

331. Kovarik JM, Eisen H, Dorent R, Mancini D, Vigano M, Rouilly M *et al*.: **Everolimus in de novo cardiac transplantation: pharmacokinetics, therapeutic range, and influence on cyclosporine exposure.** *J Heart Lung Transplant* 2003, **22:** 1117-1125.

332. Starling RC, Hare JM, Hauptman P, McCurry KR, Mayer HW, Kovarik JM *et al*.: **Therapeutic drug monitoring for everolimus in heart transplant recipients based on exposure-effect modeling.** *Am J Transplant* 2004, **4:** 2126-2131.

333. Ezzet F, Krishna G, Wexler DB, Statkevich P, Kosoglou T, Batra VK: **A population pharmacokinetic model that describes multiple peaks due to enterohepatic recirculation of ezetimibe.** *Clin Ther* 2001, **23:** 871-885.

334. Ezzet F, Wexler D, Statkevich P, Kosoglou T, Patrick J, Lipka L *et al*.: **The plasma concentration and LDL-C relationship in patients receiving ezetimibe.** *J Clin Pharmacol* 2001, **41:** 943-949.

335. AHFS: *AHFS Drug Information*. Bethesda: American Society of Health-System Pharmacists; 2002.

336. Yoshimoto K, Saima S, Echizen H, Nakamura Y, Kondo T, Yagishita Y *et al*.: **Famotidine-associated central nervous system reactions and plasma and cerebrospinal drug concentrations in neurosurgical patients with renal failure.** *Clin Pharmacol Ther* 1994, **55:** 693-700.

337. Graves NM: **Felbamate.** *Ann Pharmacother* 1993, **27:** 1073-1081.

338. Troupin AS, Montouris G, Hussein G: **Felbamate: Therapeutic range and other kinetic information.** *Epilepsy* 1997, **10:** 26-31.

339. Wagner ML: **Felbamate: a new antiepileptic drug.** *Am J Hosp Pharm* 1994, **51:** 1657-1666.

340. Bolten W, Salzmann G, Goldmann R, Miehlke K: **Plasma- und Gewebekonzentrationen von Biphenylessigsäure nach einwöchiger oraler Fenbufenmedikation bzw. topischer Anwendung von Felbinac-Gel am Kniegelenk.** *Z Rheumatol* 1989, **48:** 317-322.

341. Blychert E, Edgar B, Elmfeldt D, Hedner T: **Plasma concentration--effect relationships for felodipine: a meta analysis.** *Clin Pharmacol Ther* 1992, **52:** 80-89.

342. Lössner A, Banditt P, Troger U: **Rapid and simple method for detection of fenofibric acid in human serum by high-performance liquid chromatography.** *Pharmazie* 2001, **56:** 50-51.

343. Hercegova A, Polonsky J: **Determination of non-steroidal anti-inflammatory drugs in biological fluids.** *Pharmazie* 1999, **54:** 479-486.

344. Hug CC: **Fentanyl and sufentanil anesthesia revisted: establish an effective plasma concentration and achieve it at the right time.** *Anesthesiology* 1991, **74:** 390.

345. Lehmann KA, Freier J, Daub D: **Fentanyl-Pharmakokinetik und postoperative Atemdepression.** *Anaesthesist* 1982, **31:** 111-118.

346. Philbin DM, Rosow CE, Schneider RC, Koski G, D'Ambra MN: **Fentanyl and sufentanil anesthesia revisted: establish an effective plasma concentration and achieve it atthe right time.** *Anesthesiology* 1991, **74:** 389-390.

347. Singleton MA, Rosen JI, Fisher DM: **Plasma concentrations of fentanyl in infants, children and adults.** *Can J Anaesth* 1987, **34:** 152-155.

348. Smialek JE, Levine B, Chin L, Wu SC, Jenkins AJ: **A fentanyl epidemic in Maryland 1992.** *J Forensic Sci* 1994, **39:** 159-164.

349. Stanley TH, Bailey PL: **Fentanyl and sufentanil anesthesia revisited: establish an effective plasma concentration and achieve it at the right time.** *Anesthesiology* 1991, **74:** 388-390.

350. Yerasi AB, Butts JD, Butts JD: **Disposal of used fentanyl patches.** *Am J Health Syst Pharm* 1997, **54:** 85-86.

351. Andresen H, Gullans A, Veselinovic M, Anders S, Schmoldt A, Iwersen-Bergmann S *et al*.: **Fentanyl: toxic or therapeutic? Postmortem and antemortem blood concentrations after transdermal fentanyl application.** *J Anal Toxicol* 2012, **36:** 182-194.

352. Simons FE, Bergman JN, Watson WT, Simons KJ: **The clinical pharmacology of fexofenadine in children.** *J Allergy Clin Immunol* 1996, **98:** 1062-1064.

353. Steiner JF: **Clinical pharmacokinetics and pharmacodynamics of finasteride.** *Clin Pharmacokinet* 1996, **30:** 16-27.

354. Evers J, Eichelbaum M, Kroemer HK: **Unpredictability of flecainide plasma concentrations in patients with renal failure: relationship to side effects and sudden death?** *Ther Drug Monit* 1994, **16:** 349-351.

355. Debruyne D, Ryckelynck JP: **Clinical pharmacokinetics of fluconazole.** *Clin Pharmacokinet* 1993, **24:** 10-27.

356. Debruyne D: **Clinical pharmacokinetics of fluconazole in superficial and systemic mycoses.** *Clin Pharmacokinet* 1997, **33:** 52-77.

357. Reuman PD, Neiberger R, Kondor DA: **Intraperitoneal and intravenous fluconazole pharmokinetics in a pediatric patient with end stage renal disease.** *Pediatr Infect Dis J* 1992, **11:** 132-133.

358. Scholz J, Schulz M, Steinfath M, Hover S, Bause H: **Fluconazole is removed by continuous venovenous hemofiltration in a liver transplant patient.** *J Mol Med* 1995, **73:** 145-147.

359. Bond A, Seijas D, Dawling S, Lader M: **Systemic absorption and abuse liability of snorted flunitrazepam.** *Addiction* 1994, **89:** 821-830.

360. Pak CY, Sakhaee K, Rubin CD, Zerwekh JE: **Sustained-release sodium fluoride in the management of established postmenopausal osteoporosis.** *Am J Med Sci* 1997, **313:** 23-32.

361. Pitt P, Berry H: **Fluoride treatment in osteoporosis.** *Postgrad Med J* 1991, **67:** 323-326.

362. von Werder K, Schulz M: *Prophylaxe und Therapie der Osteoporose*. Stuttgart: WVG; 1991.

363. Henry JA: **Toxicity of antidepressants: comparisons with fluoxetine.** *Int Clin Psychopharmacol* 1992, **6 Suppl 6:22-7.:** 22-27.

364. Renshaw PF, Guimaraes AR, Fava M, Rosenbaum JF, Pearlman JD, Flood JG *et al*.: **Accumulation of fluoxetine and norfluoxetine in human brain during therapeutic administration.** *Am J Psychiatry* 1992, **149:** 1592-1594.

365. Niebch G, Borbe HO, Hummel T, Kobal G: **Dose-proportional plasma levels of the analgesic flupirtine maleate in man. Application of a new HPLC assay.** *Arzneimittelforschung* 1992, **42:** 1343-1345.

366. Forland SC, Wechter WJ, Witchwoot S, Clifford KH, Arnett RL, Cutler RE: **Human plasma concentrations of R, S, and racemic flurbiprofen given as a toothpaste.** *J Clin Pharmacol* 1996, **36:** 546-553.

367. Schulz M, Schmoldt A, Donn F, Becker H: **The pharmacokinetics of flutamide and its major metabolites after a single oral dose and during chronic treatment.** *Eur J Clin Pharmacol* 1988, **34:** 633-636.

368. Grimsley SR, Jann MW: **Paroxetine, sertraline, and fluvoxamine: new selective serotonin reuptake inhibitors.** *Clin Pharm* 1992, **11:** 930-957.

369. Wood DM, Rajalingam Y, Greene SL, Morgan PE, Gerrie D, Jones AL *et al*.: **Status epilepticus following intentional overdose of fluvoxamine: a case report with serum fluvoxamine concentration.** *Clin Toxicol (Phila)* 2007, **45:** 791-793.

370. Andrews CO, Fischer JH: **Gabapentin: a new agent for the management of epilepsy.** *Ann Pharmacother* 1994, **28:** 1188-1196.

371. Blum RA, Comstock TJ, Sica DA, Schultz RW, Keller E, Reetze P *et al*.: **Pharmacokinetics of gabapentin in subjects with various degrees of renal function.** *Clin Pharmacol Ther* 1994, **56:** 154-159.

372. Bockbrader HN: **Clinical pharmacokinetics of gabapentin.** *Drugs Today* 1995, **31:** 613-619.

373. Bockbrader HN, Wesche D, Miller R, Chapel S, Janiczek N, Burger P: **A comparison of the pharmacokinetics and pharmacodynamics of pregabalin and gabapentin.** *Clin Pharmacokinet* 2010, **49:** 661-669.

374. Btaiche IF, Woster PS: **Gabapentin and lamotrigine: novel antiepileptic drugs.** *Am J Health Syst Pharm* 1995, **52:** 61-69.

375. Knörle R, Feuerstein TJ, Schulze-Bonhage A: **Determination of gabapentin-lactam in serum of patients under gabapentin therapy.** *Arzneimittelforschung* 2004, **54:** 139-142.

376. Peruche B, Schulz M: **Gabapentin, ein neues Antiepileptikum.** *Pharm Ztg* 1996, **141:** 1396-1404.

377. Radulovic LL, Taylor CP, Walker RM: **The preclinical pharmacology, pharmacokinetics and toxicology of gabapentin.** *Drugs Today* 1995, **31:** 597-611.

378. Sivenius J, Kalviainen R, Ylinen A, Riekkinen P: **Double-blind study of Gabapentin in the treatment of partial seizures.** *Epilepsia* 1991, **32:** 539-542.

379. Spiller HA, Dunaway MD, Cutino L: **Massive gabapentin and presumptive quetiapine overdose.** *Vet Hum Toxicol* 2002, **44:** 243-244.

380. Stewart BH, Kugler AR, Thompson PR, Bockbrader HN: **A saturable transport mechanism in the intestinal absorption of gabapentin is the underlying cause of the lack of proportionality between increasing dose and drug levels in plasma.** *Pharm Res* 1993, **10:** 276-281.

381. Tomson T, Johannessen SI: **Therapeutic monitoring of the new antiepileptic drugs.** *Eur J Clin Pharmacol* 2000, **55:** 697-705.

382. Wilson EA, Sills GJ, Forrest G, Brodie MJ: **High dose gabapentin in refractory partial epilepsy: clinical observations in 50 patients.** *Epilepsy Res* 1998, **29:** 161-166.

383. Bickel U, Thomsen T, Weber W, Fischer JP, Bachus R, Nitz M *et al*.: **Pharmacokinetics of galanthamine in humans and corresponding cholinesterase inhibition.** *Clin Pharmacol Ther* 1991, **50:** 420-428.

384. Scott LJ, Goa KL: **Galantamine: a review of its use in Alzheimer's disease.** *Drugs* 2000, **60:** 1095-1122.

385. Frese JH, Rohland L, Schulz M, Schmoldt A: **Intoxikation mit Gallopamil. Verlauf und Therapie.** *Dtsch Med Wochenschr* 1988, **113:** 770-772.

386. Andresen H, Sprys N, Schmoldt A, Mueller A, Iwersen-Bergmann S: **Gamma-hydroxybutyrate in urine and serum: additional data supporting current cut-off recommendations.** *Forensic Sci Int* 2010, **200:** 93-99.

387. Andresen H, Aydin BE, Mueller A, Iwersen-Bergmann S: **An overview of gamma-hydroxybutyric acid: pharmacodynamics, pharmacokinetics, toxic effects, addiction, analytical methods, and interpretation of results.** *Drug Test Anal* 2011, **3:** 560-568.

388. Jung D, Griffy K, Wong R, Colburn W, Hulse J: **Steady-state relative bioavailability of three oral ganciclovir dosage regimens delivering 6,000 mg/day in patients with human immunodeficiency virus.** *J Clin Pharmacol* 1998, **38:** 1021-1024.

389. Brier ME, Zurada JM, Aronoff GR: **Neural network predicted peak and trough gentamicin concentrations.** *Pharm Res* 1995, **12:** 406-412.

390. Edwards DJ: **Therapeutic drug monitoring of aminoglycosides and vancomycin: guidelines and controversies.** *J Pharm Pract* 1991, **IV:** 211-224.

391. Inciardi JF, Willits NH: **Setting confidence intervals for drug concentrations from pharmacokinetic parameters.** *Ann Pharmacother* 1992, **26:** 1070-1074.

392. Kozyrskyj A, Masih M, Hahn J, Ho C, Wong M, Sirdevan M: **New neonatal gentamicin dosing guidelines: results of an evaluation of serum concentrations.** *Can J Hosp Pharm* 1994, **47:** 262-267.

393. Modi N, Maggs AF, Clarke C, Chapman C, Swann RA: **Gentamicin concentration and toxicity.** *Lancet* 1998, **352:** 70.

394. Watling SM, Kisor DF: **Population pharmacokinetics: development of a medical intensive care unit-specific gentamicin dosing nomogram.** *Ann Pharmacother* 1993, **27:** 151-154.

395. Lam YW, Jann MW, Chang WH, Yu HS, Lin SK, Chen H *et al*.: **Intra- and interethnic variability in reduced haloperidol to haloperidol ratios.** *J Clin Pharmacol* 1995, **35:** 128-136.

396. Potkin SG, Shen Y, Pardes H, Phelps BH, Zhou D, Shu L *et al*.: **Haloperidol concentrations elevated in Chinese patients.** *Psychiatry Res* 1984, **12:** 167-172.

397. Ulrich S, Wurthmann C, Brosz M, Meyer FP: **The relationship between serum concentration and therapeutic effect of haloperidol in patients with acute schizophrenia.** *Clin Pharmacokinet* 1998, **34:** 227-263.

398. Ulrich S, Meyer FP: **Reduzierte Haloperidol und Haloperidolpyridinium-Metaboliten bei schizophrenen Patienten.** *Psychopharmakotherapie* 1999, **6:** 100-102.

399. Challapalli R, Lefkovits J, Topol EJ: **Clinical trials of recombinant hirudin in acute coronary syndromes.** *Coron Artery Dis* 1996, **7:** 429-437.

400. Hagen N, Thirlwell MP, Dhaliwal HS, Babul N, Harsanyi Z, Darke AC: **Steady-state pharmacokinetics of hydromorphone and hydromorphone-3-glucuronide in cancer patients after immediate and controlled-release hydromorphone.** *J Clin Pharmacol* 1995, **35:** 37-44.

401. Mason PE, Kerns WP: **Gamma hydroxybutyric acid (GHB) intoxication.** *Acad Emerg Med* 2002, **9:** 730-739.

402. Steinecke H: **Beitrag zur Bewertung von Gamma-Hydroxybuttersäure (GBH) - Konzentrationen im Blut lebender Personen sowie in postmortalem Blut.** *Toxichem Krimtech* 2007, **74:** 150-154.

403. Kunze K, Kauert U, Schmoldt A: **Drug induced myopathy by hydroxychloroquine.** *Vet Hum Toxicol* 1987, **29:** 59-60.

404. Abraham TT, Barnes AJ, Lowe RH, Kolbrich Spargo EA, Milman G, Pirnay SO *et al*.: **Urinary MDMA, MDA, HMMA, and HMA excretion following controlled MDMA administration to humans.** *J Anal Toxicol* 2009, **33:** 439-446.

405. Barnes AJ, Scheidweiler KB, Kolbrich-Spargo EA, Gorelick DA, Goodwin RS, Huestis MA: **MDMA and metabolite disposition in expectorated oral fluid after controlled oral MDMA administration.** *Ther Drug Monit* 2011, **33:** 602-608.

406. Kolbrich EA, Goodwin RS, Gorelick DA, Hayes RJ, Stein EA, Huestis MA: **Plasma pharmacokinetics of 3,4-methylenedioxymethamphetamine after controlled oral administration to young adults.** *Ther Drug Monit* 2008, **30:** 320-332.

407. Forsyth DR, Jayasinghe KS, Roberts CJ: **Do nizatidine and cimetidine interact with ibuprofen?** *Eur J Clin Pharmacol* 1988, **35:** 85-88.

408. Holubek WJ, Wetter A, Howland MA, Hoffman RS, Nelson LS: **Death from a massive ibuprofen overdose [abstract].** *Clin Toxicol* 2006, **44:** 488.

409. Pisano P, Durand A, Autret E, Desnuelle C, Pinsard N, Serratrice G *et al*.: **Plasma concentrations and pharmacokinetics of idebenone and its metabolites following single and repeated doses in young patients with mitochondrial encephalomyopathy.** *Eur J Clin Pharmacol* 1996, **51:** 167-169.

410. Druker BJ, Talpaz M, Resta DJ, Peng B, Buchdunger E, Ford JM *et al*.: **Efficacy and safety of a specific inhibitor of the BCR-ABL tyrosine kinase in chronic myeloid leukemia.** *N Engl J Med* 2001, **344:** 1031-1037.

411. Lyseng-Williamson K, Jarvis B: **Imatinib.** *Drugs* 2001, **61:** 1765-1774.

412. Tegeder I, Bremer F, Oelkers R, Schüttler J, Brune K, Geisslinger G: **Therapeutic drug monitoring of imipenem during continuous veno-venous hemofiltrationn [abstract].** *Naunyn-Schmiedeberg's Arch Pharmacol* 1996, **355:** R129.

413. Jorgensen OS, Lober M, Christiansen J, Gram LF: **Plasma concentration and clinical effect in imipramine treatment of childhood enuresis.** *Clin Pharmacokinet* 1980, **5:** 386-393.

414. Rayner CR, Galbraith KJ, Marriott JL, Duncan GJ: **A critical evaluation of the therapeutic range of indinavir.** *Ann Pharmacother* 2002, **36:** 1230-1237.

415. Gilman JT, Gal P: **Pharmacokinetic and pharmacodynamic data collection in children and neonates. A quiet frontier.** *Clin Pharmacokinet* 1992, **23:** 1-9.

416. Mistry GC, Jensen BK, Rakhit A, Huselton CA, Patel IH: **Systemic availability of retinoids following excessive topical application of isotrex to patients with acne vulgaris [abstract].** *Pharm Res* 1995, **12:** S-414.

417. Nulman I, Berkovitch M, Klein J, Pastuszak A, Lester RS, Shear N *et al*.: **Steady-state pharmacokinetics of isotretinoin and its 4-oxo metabolite: implications for fetal safety.** *J Clin Pharmacol* 1998, **38:** 926-930.

418. Chellingsworth MD, Willis JV, Jack DB, Kendall MJ: **Pharmacokinetics and pharmacodynamics of isradipine (PN200-110) in young and elderly patients.** *Am J Med* 1988, **72:** 72-79.

419. Romano MJ, Gaylor A, Sang CJ, Jr.: **Life-threatening isradipine poisoning in a child.** *Pharmacotherapy* 2002, **22:** 766-770.

420. Tse FL, Jaffe JM: **Pharmacokinetics of PN 200-110 (isradipine), a new calcium antagonist, after oral administration in man.** *Eur J Clin Pharmacol* 1987, **32:** 361-365.

421. Barone JA, Moskovitz BL, Guarnieri J, Hassell AE, Colaizzi JL, Bierman RH *et al*.: **Food interaction and steady-state pharmacokinetics of itraconazole oral solution in healthy volunteers.** *Pharmacotherapy* 1998, **18:** 295-301.

422. Lipp H-P: **Klinische Pharmakokinetik von Itraconazol.** *Krankenhauspharmazie* 1996, **17:** 388-395.

423. Neuvonen PJ, Varhe A, Olkkola KT: **The effect of ingestion time interval on the interaction between itraconazole and triazolam.** *Clin Pharmacol Ther* 1996, **60:** 326-331.

424. Slain D, Rogers PD, Cleary JD, Chapman SW: **Intravenous itraconazole.** *Ann Pharmacother* 2001, **35:** 720-729.

425. Okonkwo PO, Ogbuokiri JE, Ofoegbu E, Klotz U: **Protein binding and ivermectin estimations in patients with onchocerciasis.** *Clin Pharmacol Ther* 1993, **53:** 426-430.

426. Bondesson U, Hartvig P, Danielsson B: **Quantitative determination of the urinary excretion of ketobemidone and four of its metabolites after intravenous and oral administration in man.** *Drug Metab Dispos* 1981, **9:** 376-380.

427. Hartvig P, Valtysson J, Lindner KJ, Kristensen J, Karlsten R, Gustafsson LL *et al*.: **Central nervous system effects of subdissociative doses of (S)-ketamine are related to plasma and brain concentrations measured with positron emission tomography in healthy volunteers.** *Clin Pharmacol Ther* 1995, **58:** 165-173.

428. Steentoft A, Worm K: **Cases of fatal intoxication with Ketogan.** *J Forensic Sci Soc* 1994, **34:** 181-185.

429. Ballerini R, Casini A, Chinol M, Mannucci C, Giaccai L, Salvi M: **Study on the absorption of ketoprofen topically administered in man: comparison between tissue and plasma levels.** *Int J Clin Pharmacol Res* 1986, **6:** 69-72.

430. Dionne RA, Gordon SM, Tahara M, Rowan J, Troullos E: **Analgesic efficacy and pharmacokinetics of ketoprofen administered into a surgical site.** *J Clin Pharmacol* 1999, **39:** 131-138.

431. Ishizaki T, Sasaki T, Suganuma T, Horai Y, Chiba K, Watanabe M *et al*.: **Pharmacokinetics of ketoprofen following single oral, intramuscular and rectal doses and after repeated oral administration.** *Eur J Clin Pharmacol* 1980, **18:** 407-414.

432. Lewellen ORW, Templeton RT: **The pharmacokinetics of ketoprofen in man during and after repeated oral dosing (50 mg q.i.d.) with Orudis(R) [abstract].** *Scand J Rheumatol* 1976, **14:** 53-62.

433. Netter P, Bannwarth B, Lapicque F, Harrewyn JM, Frydman A, Tamisier JN *et al*.: **Total and free ketoprofen in serum and synovial fluid after intramuscular injection.** *Clin Pharmacol Ther* 1987, **42:** 555-561.

434. Johnson MA, Moore KH, Yuen GJ, Bye A, Pakes GE: **Clinical pharmacokinetics of lamivudine.** *Clin Pharmacokinet* 1999, **36:** 41-66.

435. Rambeck B, Wolf P: **Lamotrigine clinical pharmacokinetics.** *Clin Pharmacokinet* 1993, **25:** 433-443.

436. Schapel GJ, Beran RG, Vajda FJ, Berkovic SF, Mashford ML, Dunagan FM *et al*.: **Double-blind, placebo controlled, crossover study of lamotrigine in treatment resistant partial seizures.** *J Neurol Neurosurg Psychiatry* 1993, **56:** 448-453.

437. French LK, McKeown NJ, Hendrickson RG: **Complete heart block and death following lamotrigine overdose.** *Clin Toxicol (Phila)* 2011, **49:** 330-333.

438. Schaller K-H, Angerer H, Lehnert G: **Bio-Monitoring in der Arbeits- und Umweltmedizin.** *Dt Ärztebl* 1993, **90:** C-1430-C-1435.

439. Schmid I, Paulweber B, Pechböck W, Oberkofler H, Patsch W: **Eine spät erkannte Bleiintoxikation.** *Toxichem Krimtech* 2000, **67:** 96-97.

440. Beaman JM, Hackett LP, Luxton G, Illett KF: **Effect of hemodialysis on leflunomide plasma concentrations.** *Ann Pharmacother* 2002, **36:** 75-77.

441. Goldenberg MM: **Leflunomide, a novel immunomodulator for the treatment of active rheumatoid arthritis.** *Clin Ther* 1999, **21:** 1837-1852.

442. Lucien J, Dias VC, LeGatt DF, Yatscoff RW: **Blood distribution and single-dose pharmacokinetics of leflunomide.** *Ther Drug Monit* 1995, **17:** 454-459.

443. Barrueto F, Jr., Williams K, Howland MA, Hoffman RS, Nelson LS: **A case of levetiracetam (Keppra) poisoning with clinical and toxicokinetic data.** *J Toxicol Clin Toxicol* 2002, **40:** 881-884.

444. Heykants J, Van PA, Van dV, V, Snoeck E, Meuldermans W, Woestenborghs R: **The pharmacokinetic properties of topical levocabastine. A review.** *Clin Pharmacokinet* 1995, **29:** 221-230.

445. Baas H, Harder S, Burklin F, Demisch L, Fischer PA: **Pharmacodynamics of levodopa coadministered with apomorphine in parkinsonian patients with end-of-dose motor fluctuations.** *Clin Neuropharmacol* 1998, **21:** 86-92.

446. Harder S, Baas H, Rietbrock S: **Concentration-effect relationship of levodopa in patients with Parkinson's disease.** *Clin Pharmacokinet* 1995, **29:** 243-256.

447. Harder S, Baas H, Bergemann N, Demisch L, Rietbrock S: **Concentration-effect relationship of levodopa in patients with Parkinson's disease after oral administration of an immediate release and a controlled release formulation.** *Br J Clin Pharmacol* 1995, **39:** 39-44.

448. Harder S, Baas H: **Concentration-response relationship of levodopa in patients at different stages of Parkinson's disease.** *Clin Pharmacol Ther* 1998, **64:** 183-191.

449. Sturner WQ, Garriott JC: **L-dopa poisoning.** *J Forensic Sci* 1972, **17:** 440-443.

450. Schall U, Katta T, Pries E, Klöppel A, Gastpar M: **Dosierung von Levomethadon in der Substitutionsbehandlung i.v.-Opiatabhängiger.** *Dt Ärztebl* 1994, **91:** C-556-C-557.

451. Foy JL, Eastman RC, Nealon RC, Bowen PM, Pengelly ML, Drass JA *et al*.: **Automated therapeutic drug monitoring in an ambulatory care endocrine clinic.** *Ann Pharmacother* 1992, **26:** 675-678.

452. Dawling S, Flanagan RJ, Widdop B: **Fatal lignocaine poisoning: report of two cases and review of the literature.** *Hum Toxicol* 1989, **8:** 389-392.

453. den Hartigh J, Hilders CG, Schoemaker RC, Hulshof JH, Cohen AF, Vermeij P: **Tinnitus suppression by intravenous lidocaine in relation to its plasma concentration.** *Clin Pharmacol Ther* 1993, **54:** 415-420.

454. Wu FL, Razzaghi A, Souney PF: **Seizure after lidocaine for bronchoscopy: case report and review of the use of lidocaine in airway anesthesia.** *Pharmacotherapy* 1993, **13:** 72-78.

455. Alderman CP, Lindsay KS: **Increased serum lithium concentration secondary to treatment with tiaprofenic acid and fosinopril.** *Ann Pharmacother* 1996, **30:** 1411-1413.

456. Goff DC, Baldessarini RJ: **Drug interactions with antipsychotic agents.** *J Clin Psychopharmacol* 1993, **13:** 57-67.

457. Grobosch T, Schönberg L, Lampe D: **Toxikologisches Monitoring von Risperidon und Lithium bei einer akuten Intoxikation.** *Toxichem Krimtech* 2004, **71:** 10-16.

458. Müller N, Kapfhammer HP, Spatz R, Hippius H: **Die Lithium-Prophylaxe: hohe Effizienz und geringes Risiko bei regelmäßiger Überwachung.** *Dt Ärztebl* 1995, **92:** C-236-C-239.

459. Killinger JM, Weintraub HS, Fuller BL: **Human pharmacokinetics and comparative bioavailability of loperamide hydrochloride.** *J Clin Pharmacol* 1979, **19:** 211-218.

460. Radwanski E, Hilbert J, Symchowicz S, Zampaglione N: **Loratadine: multiple-dose pharmacokinetics.** *J Clin Pharmacol* 1987, **27:** 530-533.

461. Zhong D, Blume H: **HPLC-Bestimmung von Loratadin und seinem aktiven Metaboliten Descarboethoxyloratadin in Humanplasma.** *Pharmazie* 1994, **49:** 736-739.

462. Henry DW, Burwinkle JW, Klutman NE: **Determination of sedative and amnestic doses of lorazepam in children.** *Clin Pharm* 1991, **10:** 625-629.

463. Reiter PD, Stiles AD: **Lorazepam toxicity in a premature infant.** *Ann Pharmacother* 1993, **27:** 727-729.

464. Gillis AM, Kates RE: **Clinical pharmacokinetics of the newer antiarrhythmic agents.** *Clin Pharmacokinet* 1984, **9:** 375-403.

465. Mead RH, Keefe DL, Kates RE, Winkle RA: **Chronic lorcainide therapy for symptomatic premature ventricular complexes: efficacy, pharmacokinetics and evidence for norlorcainide antiarrhythmic effect.** *Am J Cardiol* 1985, **55:** 72-78.

466. Somani P, Simon V, Gupta RK, King P, Shapiro RS, Stockard H: **Lorcainide kinetics and protein binding in patients with end-stage renal disease.** *Int J Clin Pharmacol Ther Toxicol* 1984, **22:** 121-125.

467. Ludewig R: *Akute Vergiftungen*. Stuttgart: WVG; 1999.

468. Wilimzig C, Latz R, Vierling W, Mutschler E, Trnovec T, Nyulassy S: **Increase in magnesium plasma level after orally administered trimagnesium dicitrate.** *Eur J Clin Pharmacol* 1996, **49:** 317-323.

469. Burgmann H, Winkler S, Uhl F, Feucht M, Hellgren U, Bergqvist Y *et al*.: **Mefloquin und Sulfadoxin/Pyrimethamin-Überdosierung bei Malaria tropica.** *Wien Klin Wochenschr* 1993, **105:** 61-63.

470. Hellgren U, Jastrebova J, Jerling M, Krysen B, Bergqvist Y: **Comparison between concentrations of racemic mefloquine, its separate enantiomers and the carboxylic acid metabolite in whole blood serum and plasma.** *Eur J Clin Pharmacol* 1996, **51:** 171-173.

471. Noble S, Balfour JA: **Meloxicam.** *Drugs* 1996, **51:** 424-430.

472. Hui WK, Mitchell LB, Kavanagh KM, Gillis AM, Wyse DG, Manyari DE *et al*.: **Melperone: electrophysiologic and antiarrhythmic activity in humans.** *J Cardiovasc Pharmacol* 1990, **15:** 144-149.

473. Stein S, Schmoldt A, Schulz M: **Fatal intoxication with melperone.** *Forensic Sci Int* 2000, **113:** 409-413.

474. N.N.: **Quecksilber-Referenzwerte.** *Bundesgesundhbl* 1998, **41:** 270.

475. Singer AJ, Mofenson HC, Caraccio TR, Ilasi J: **Mercuric chloride poisoning due to ingestion of a stool fixative.** *Clin Toxicol* 1994, **32:** 577-582.

476. Bernhoft RA: **Mercury toxicity and treatment: a review of the literature.** *J Environ Public Health* 2012, **2012:** 460508.

477. Akyildiz BN, Kondolot M, Kurtoglu S, Konuskan B: **Case series of mercury toxicity among children in a hot, closed environment.** *Pediatr Emerg Care* 2012, **28:** 254-258.

478. Dargan PI, Giles LJ, Wallace CI, House IM, Thomson AH, Beale RJ *et al*.: **Case report: severe mercuric sulphate poisoning treated with 2,3-dimercaptopropane-1-sulphonate and haemodiafiltration.** *Crit Care* 2003, **7:** R1-R6.

479. Klotz U, Stracciari GL: **Steady state disposition of 5-aminosalicyclic acid following oral dosing.** *Arzneim -Forsch /Drug Res* 1993, **43:** 1357-1359.

480. Desel H, Stedtler U, Behrens A, Neuratz H: **Mischintoxikation mit Metformin.** *Toxichem Krimtech* 2000, **67:** 4-8.

481. Reeker W, Schneider G, Felgenhauer N, Tempel G, Kochs E: **Metformin-induzierte Laktazidose.** *Dtsch Med Wochenschr* 2000, **125:** 249-251.

482. Chugh SS, Socoteanu C, Reinier K, Waltz J, Jui J, Gunson K: **A community-based evaluation of sudden death associated with therapeutic levels of methadone.** *Am J Med* 2008, **121:** 66-71.

483. Horns WH, Rado M, Goldstein A: **Plasma levels and symptom complaints in patients maintained on daily dosage of methadone hydrochloride.** *Clin Pharmacol Ther* 1975, **17:** 636-649.

484. Inturrisi CE, Colburn WA, Kaiko RF, Houde RW, Foley KM: **Pharmacokinetics and pharmacodynamics of methadone in patients with chronic pain.** *Clin Pharmacol Ther* 1987, **41:** 392-401.

485. Inturrisi CE, Verebely K: **The levels of methadone in the plasma in methadone maintenance.** *Clin Pharmacol Ther* 1972, **13:** 633-637.

486. Schmidt N, Sittl R, Brune K, Geisslinger G: **Rapid determination of methadone in plasma, cerebrospinal fluid, and urine by gas chromatography and its application to routine drug monitoring.** *Pharm Res* 1993, **10:** 441-444.

487. Ufkes JG, de Vos JW, Geerlings PJ, van Wilgenburg H: **Determination of methadone and its primary metabolite in twenty opiate addicts [abstract].** *Pharm World Sci* 1994, **16:** D6.

488. Heinemann A, Iwersen-Bergmann S, Stein S, Schmoldt A, Puschel K: **Methadone-related fatalities in Hamburg 1990-1999: implications for quality standards in maintenance treatment?** *Forensic Sci Int* 2000, **113:** 449-455.

489. Karch SB: **Is it time to reformulate racemic methadone?** *J Addict Med* 2011, **5:** 229-231.

490. Epker JL, Bakker J: **Accidental methanol ingestion: case report.** *BMC Emerg Med* 2010, **10:** 3.

491. Stefan H: **Epilepsietherapie. Teil 1: Konservative Behandlung.** *Dt Ärztebl* 1998, **95:** C-2204-C-2210.

492. Schmoldt A, Iwersen S, Schluter W: **Massive ingestion of the herbicide 2-methyl-4-chlorophenoxyacetic acid (MCPA).** *J Toxicol Clin Toxicol* 1997, **35:** 405-408.

493. Kraemer T, Maurer HH: **Toxicokinetics of amphetamines: metabolism and toxicokinetic data of designer drugs, amphetamine, methamphetamine, and their N-alkyl derivatives.** *Ther Drug Monit* 2002, **24:** 277-289.

494. Moore KA, Mozayani A, Fierro MF, Poklis A: **Distribution of 3,4-methylenedioxymethamphetamine (MDMA) and 3,4-methylenedioxyamphetamine (MDA) stereoisomers in a fatal poisoning.** *Forensic Sci Int* 1996, **83:** 111-119.

495. Buechler J, Schwab M, Mikus G, Fischer B, Hermle L, Marx C *et al*.: **Enantioselective quantitation of the ecstasy compound (R)- and (S)-N-ethyl-3,4-methylenedioxyamphetamine and its major metabolites in human plasma and urine.** *J Chromatogr B Analyt Technol Biomed Life Sci* 2003, **793:** 207-222.

496. Freudenmann RW, Spitzer M: **The Neuropsychopharmacology and Toxicology of 3,4-methylenedioxy-N-ethyl-amphetamine (MDEA).** *CNS Drug Rev* 2004, **10:** 89-116.

497. Meyer MR, Peters FT, Maurer HH: **The role of human hepatic cytochrome P450 isozymes in the metabolism of racemic 3,4-methylenedioxyethylamphetamine and its single enantiomers.** *Drug Metab Dispos* 2009, **37:** 1152-1156.

498. Cami J, de la Torre R, Ortuno J, Farre M, Mas M, Roset PN *et al*.: **Pharmacokinetics of ecstasy (MDMA) in healthy subjects [abstract].** *Eur J Clin Pharmacol* 1997, **52:** A168.

499. Fallon JK, Kicman AT, Henry JA, Milligan PJ, Cowan DA, Hutt AJ: **Stereospecific analysis and enantiomeric disposition of 3, 4-methylenedioxymethamphetamine (Ecstasy) in humans.** *Clin Chem* 1999, **45:** 1058-1069.

500. de Boer D., Egberts T, Maes RA: **Para-methylthioamphetamine, a new amphetamine designer drug of abuse.** *Pharm World Sci* 1999, **21:** 47-48.

501. Elliott SP: **Fatal poisoning with a new phenylethylamine: 4-methylthioamphetamine (4-MTA).** *J Anal Toxicol* 2000, **24:** 85-89.

502. Poortman AJ, Lock E: **Analytical profile of 4-methylthioamphetamine (4-MTA), a new street drug.** *Forensic Sci Int* 1999, **100:** 221-233.

503. Tarbah FA, Zweipfennig P, Pier S, Temme O, Daldrup T: **Tödliche Vergiftung mit dem Amphetaminderivat 4-MTA.** *Toxichem Krimtech* 2001, **68:** 21.

504. Weise M: **Das traurige Ende einer "Geburtstagsfeier" - Fatale Intoxikation mit 4-Methylthioamphetamin.** *Toxichem Krimtech* 2001, **68:** 38-42.

505. Meibohm B, Wegener S: **Mexiletin-Theophyllin-Interaktion. Pharmakokinetische Auswirkungen und klinische Relevanz.** *Krankenhauspharmazie* 1992, **13:** 331-333.

506. Backman JT, Olkkola KT, Neuvonen PJ: **Rifampin drastically reduces plasma concentrations and effects of oral midazolam.** *Clin Pharmacol Ther* 1996, **59:** 7-13.

507. Blumer JL: **Clinical pharmacology of midazolam in infants and children.** *Clin Pharmacokinet* 1998, **35:** 37-47.

508. Hughes J, Gill AM, Mulhearn H, Powell E, Choonara I: **Steady-state plasma concentrations of midazolam in critically ill infants and children.** *Ann Pharmacother* 1996, **30:** 27-30.

509. Heikinheimo O: **Clinical pharmacokinetics of mifepristone.** *Clin Pharmacokinet* 1997, **33:** 7-17.

510. Stimmel GL, Dopheide JA, Stahl SM: **Mirtazapine: an antidepressant with noradrenergic and specific serotonergic effects.** *Pharmacotherapy* 1997, **17:** 10-21.

511. Tang OS, Schweer H, Seyberth HW, Lee SW, Ho PC: **Pharmacokinetics of different routes of administration of misoprostol.** *Hum Reprod* 2002, **17:** 332-336.

512. Tang OS, Schweer H, Lee SW, Ho PC: **Pharmacokinetics of repeated doses of misoprostol.** *Hum Reprod* 2009, **24:** 1862-1869.

513. Committee for Medicinal Products for Human Use (CHMP). **Lysodren (mitotane) EPAR - Product information.** [European Medicines Agency] (last accessed May 6, 2011)

514. Fulton B, Benfield P: **Moclobemide. An update of its pharmacological properties and therapeutic use.** *Drugs* 1996, **52:** 450-474.

515. Hackett LP, Joyce DA, Hall RW, Dusci LJ, Ilett KF: **Disposition and clinical effects of moclobemide and three of its metabolites following overdose.** *Drug Invest* 1993, **5:** 281-284.

516. Iwersen S, Schmoldt A: **Three suicide attempts with moclobemide.** *J Toxicol Clin Toxicol* 1996, **34:** 223-225.

517. Mayersohn M, Guentert TW: **Clinical pharmacokinetics of the monoamine oxidase-A inhibitor moclobemide.** *Clin Pharmacokinet* 1995, **29:** 292-332.

518. Myrenfors PG, Eriksson T, Sandsted CS, Sjoberg G: **Moclobemide overdose.** *J Intern Med* 1993, **233:** 113-115.

519. Neuvonen PJ, Pohjola-Sintonen S, Tacke U, Vuori E: **Five fatal cases of serotonin syndrome after moclobemide-citalopram or moclobemide-clomipramine overdoses.** *Lancet* 1993, **342:** 1419.

520. Moachon G, Kanmacher I, Clenet M, Matinier D: **Pharmacokinetic profile of modafinil.** *Drugs Today* 1996, **32:** 327-337.

521. Zhao JJ, Rogers JD, Holland SD, Larson P, Amin RD, Haesen R *et al*.: **Pharmacokinetics and bioavailability of montelukast sodium (MK-0476) in healthy young and elderly volunteers.** *Biopharm Drug Dispos* 1997, **18:** 769-777.

522. Aderjan R, Schmitt G, Hofmann S: **Morphin und dessen Glucuronide im Serum von Heroinabhängigen.** *Toxichem Krimtech* 1994, **61:** 24-29.

523. Glare PA, Walsh TD: **Clinical pharmacokinetics of morphine.** *Ther Drug Monit* 1991, **13:** 1-23.

524. June HL, Stitzer ML, Cone E: **Acute physical dependence: time course and relation to human plasma morphine concentrations.** *Clin Pharmacol Ther* 1995, **57:** 270-280.

525. Lugo RA, Kern SE: **Clinical pharmacokinetics of morphine.** *J Pain Palliat Care Pharmacother* 2002, **16:** 5-18.

526. Behrend M: **Mycophenolate mofetil: suggested guidelines for use in kidney transplantation.** *BioDrugs* 2001, **15:** 37-53.

527. Fulton B, Markham A: **Mycophenolate mofetil. A review of its pharmacodynamic and pharmacokinetic properties and clinical efficacy in renal transplantation.** *Drugs* 1996, **51:** 278-298.

528. Hübner GI, Eismann R, Sziegoleit W: **Relationship between mycophenolate mofetil side effects and mycophenolic acid plasma trough levels in renal transplant patients.** *Arzneim -Forsch /Drug Res* 2000, **50:** 936-940.

529. Sanquer S, Breil M, Baron C, Dahmane D, Astier A, Lang P: **Trough blood concentrations in long-term treatment with mycophenolate mofetil.** *Lancet* 1998, **351:** 1557.

530. Willkens RF: **An overview of the long-term safety experience of nabumetone.** *Drugs* 1990, **40:** 34-37.

531. Derungs A, Schietzel S, Meyer MR, Maurer HH, Krahenbuhl S, Liechti ME: **Sympathomimetic toxicity in a case of analytically confirmed recreational use of naphyrone (naphthylpyrovalerone).** *Clin Toxicol (Phila)* 2011, **49:** 691-693.

532. Davies NM, Anderson KE: **Clinical pharmacokinetics of naproxen.** *Clin Pharmacokinet* 1997, **32:** 268-293.

533. Marzo A, Dal BL, Wool C, Cerutti R: **Bioavailability, food effect and tolerability of S-naproxen betainate sodium salt monohydrate in steady state.** *Arzneimittelforschung* 1998, **48:** 935-940.

534. Heinroth KM, Kuhn C, Walper R, Busch I, Winkler M, Prondzinsky R: **Akute Intoxikation mit dem ß1-selektiven ß-Rezeptorenblocker Nebivolol in suizidaler Absicht.** *Dtsch Med Wochenschr* 1999, **124:** 1230-1234.

535. Himmelmann A, Hedner T, Snoeck E, Lundgren B, Hedner J: **Haemodynamic effects and pharmacokinetics of oral d- and l-nebivolol in hypertensive patients.** *Eur J Clin Pharmacol* 1996, **51:** 259-264.

536. McNeely W, Goa KL: **Nebivolol in the management of essential hypertension: a review.** *Drugs* 1999, **57:** 633-651.

537. Barbhaiya RH, Shukla UA, Chaikin P, Greene DS, Marathe PH: **Nefazodone pharmacokinetics: assessment of nonlinearity, intra-subject variability and time to attain steady-state plasma concentrations after dose escalation and de-escalation.** *Eur J Clin Pharmacol* 1996, **50:** 101-107.

538. Davis R, Whittington R, Bryson HM: **Nefazodone. A review of its pharmacology and clinical efficacy in the management of major depression.** *Drugs* 1997, **53:** 608-636.

539. Gaffney PN, Schuckman HA, Beeson MS: **Nefazodone overdose.** *Ann Pharmacother* 1998, **32:** 1249-1250.

540. Greene DS, Barbhaiya RH: **Clinical pharmacokinetics of nefazodone.** *Clin Pharmacokinet* 1997, **33:** 260-275.

541. Kaul S, Shukla UA, Barbhaiya RH: **Nonlinear pharmacokinetics of nefazodone after escalating single and multiple oral doses.** *J Clin Pharmacol* 1995, **35:** 830-839.

542. Salazar DE, Marathe PH, Fulmor IE, Lee JS, Raymond RH, Uderman HD: **Pharmacokinetic and pharmacodynamic evaluation during coadministration of nefazodone and propranolol in healthy men.** *J Clin Pharmacol* 1995, **35:** 1109-1118.

543. Calvey TN, Wareing M, Williams NE, Chan K: **Pharmacokinetics and pharmacological effects of neostigmine in man.** *Br J Clin Pharmacol* 1979, **7:** 149-155.

544. Kadoya C, Domino EF, Matsuoka S: **Relationship of electroencephalographic and cardiovascular changes to plasma nicotine levels in tobacco smokers.** *Clin Pharmacol Ther* 1994, **55:** 370-377.

545. Schneider S, Diederich N, Appenzeller B, Schartz A, Lorang C, Wennig R: **Internet suicide guidelines: Report of a life threatening poisoning using tobacco extract.** *Toxichem Krimtech* 2008, **75:** 134-136.

546. Bernareggi A: **Clinical pharmacokinetics of nimesulide.** *Clin Pharmacokinet* 1998, **35:** 247-274.

547. Bakdash A, Ganswindt M, Herre S, Nakulski T, Pragst F: **Lethal poisoning with p-nitroaniline.** *Toxichem Krimtech* 2006, **73:** 61-65.

548. Moller Jensen K, Berg Dahl J: **Plasma concentrations of glyceryl trinitrate and its dinitrate metabolites after sublingual administration to volunteers. Simultaneous determination of glyceryl trinitrate and its dinitrate metabolites.** *Arzneim Forsch /Drug Res* 1994, **44:** 951-953.

549. Thiermann H, Mast U, Eyer P, Hilber A, Pfab R, Felgenhauer J *et al*.: **Parathion poisoning: pharmacokinetics and laboratory findings during continuous infusion of obidoime and atropine [abstract].** *Naunyn-Schmiedeberg's Arch Pharmacol* 1996, **353:** R146.

550. Lamp KC, Bailey EM, Rybak MJ: **Ofloxacin clinical pharmacokinetics.** *Clin Pharmacokinet* 1992, **22:** 32-46.

551. Callaghan JT, Bergstrom RF, Ptak LR, Beasley CM: **Olanzapine. Pharmacokinetic and pharmacodynamic profile.** *Clin Pharmacokinet* 1999, **37:** 177-193.

552. Citrome L, Stauffer VL, Chen L, Kinon BJ, Kurtz DL, Jacobson JG *et al*.: **Olanzapine plasma concentrations after treatment with 10, 20, and 40 mg/d in patients with schizophrenia: an analysis of correlations with efficacy, weight gain, and prolactin concentration.** *J Clin Psychopharmacol* 2009, **29:** 278-283.

553. Elian AA: **Fatal overdose of olanzepine.** *Forensic Sci Int* 1998, **91:** 231-235.

554. Perry PJ, Lund BC, Sanger T, Beasley C: **Olanzapine plasma concentrations and clinical response: acute phase results of the North American Olanzapine Trial.** *J Clin Psychopharmacol* 2001, **21:** 14-20.

555. Shrestha M, Hendrickson RG, Henretig FM: **Striking extrapyramidal movements seen in large olanzapine overdose.** *Clin Toxicol* 2001, **39:** 282.

556. Kees F, Jehkul A, Bucher M, Mair G, Kiermaier J, Grobecker H: **Bioavailability of opipramol from a film-coated tablet, a sugar-coated tablet and an aqueous solution in healthy volunteers.** *Arzneimittelforschung* 2003, **53:** 87-92.

557. Van Herreweghe I, Mertens K, Maes V, Ramet J: **Orphenadrine poisoning in a child: clinical and analytical data.** *Intensive Care Med* 1999, **25:** 1134-1136.

558. Gonzalez-Esquivel DF, Ortega-Gavilan M, Alcantara-Lopez G, Jung-Cook H: **Plasma level monitoring of oxcarbazepine in epileptic patients.** *Arch Med Res* 2000, **31:** 202-205.

559. May TW, Korn-Merker E, Rambeck B: **Clinical pharmacokinetics of oxcarbazepine.** *Clin Pharmacokinet* 2003, **42:** 1023-1042.

560. Theisohn M, Heimann G: **Disposition of the antiepileptic oxcarbazepine and its metabolites in healthy volunteers.** *Eur J Clin Pharmacol* 1982, **22:** 545-551.

561. Cremers S, Sparidans R, den HJ, Hamdy N, Vermeij P, Papapoulos S: **A pharmacokinetic and pharmacodynamic model for intravenous bisphosphonate (pamidronate) in osteoporosis.** *Eur J Clin Pharmacol* 2002, **57:** 883-890.

562. Vandenbrom RH, Wierda JM: **Pancuronium bromide in the intensive care unit: a case of overdose.** *Anesthesiology* 1988, **69:** 996-997.

563. Bond GR, Krenzelok EP, Normann SA, Tendler JD, Morris-Kukoski CL, McCoy DJ *et al*.: **Acetaminophen ingestion in childhood--cost and relative risk of alternative referral strategies.** *J Toxicol Clin Toxicol* 1994, **32:** 513-525.

564. Graudins A, Aaron CK, Linden CH: **Overdose of extended-release acetaminophen.** *N Engl J Med* 1995, **333:** 196.

565. Kamali F, Edwards C, Rawlins MD: **The effect of pirenzepine on gastric emptying and salivary flow rate: constraints on the use of saliva paracetamol concentrations for the determination of paracetamol pharmacokinetics.** *Br J Clin Pharmacol* 1992, **33:** 309-312.

566. van der Marel CD, van Lingen RA, Pluim MA, Scoones G, van DM, Vaandrager JM *et al*.: **Analgesic efficacy of rectal versus oral acetaminophen in children after major craniofacial surgery.** *Clin Pharmacol Ther* 2001, **70:** 82-90.

567. Nielsen JC, Bjerring P, rendt-Nielsen L: **A comparison of the hypoalgesic effect of paracetamol in slow-release and plain tablets on laser-induced pain.** *Br J Clin Pharmacol* 1991, **31:** 267-270.

568. Rose SR: **Subtleties of managing acetaminophen poisoning.** *Am J Hosp Pharm* 1994, **51:** 3065-3068.

569. Schiodt FV, Ott P, Christensen E, Bondesen S: **The value of plasma acetaminophen half-life in antidote-treated acetaminophen overdosage.** *Clin Pharmacol Ther* 2002, **71:** 221-225.

570. Smilkstein MJ, Douglas DR, Daya MR: **Acetaminophen poisoning and liver function.** *N Engl J Med* 1994, **331:** 1310-1311.

571. Vale JA, Proudfoot AT: **Paracetamol (acetaminophen) poisoning.** *Lancet* 1995, **346:** 547-552.

572. Hart TB, Nevitt A, Whitehead A: **A new statistical approach to the prognostic significance of plasma paraquat concentrations.** *Lancet* 1984, **2:** 1222-1223.

573. Bismuth C, Garnier R, Baud FJ, Muszynski J, Keyes C: **Paraquat poisoning. An overview of the current status.** *Drug Saf* 1990, **5:** 243-251.

574. Dinis-Oliveira RJ, Duarte JA, Sanchez-Navarro A, Remiao F, Bastos ML, Carvalho F: **Paraquat poisonings: mechanisms of lung toxicity, clinical features, and treatment.** *Crit Rev Toxicol* 2008, **38:** 13-71.

575. Fairshter RD, Dabir-Vaziri N, Smith WR, Glauser FL, Wilson AF: **Paraquat poisoning: an analytical toxicologic study of three cases.** *Toxicology* 1979, **12:** 259-266.

576. Gil HW, Kang MS, Yang JO, Lee EY, Hong SY: **Association between plasma paraquat level and outcome of paraquat poisoning in 375 paraquat poisoning patients.** *Clin Toxicol (Phila)* 2008, **46:** 515-518.

577. Gawarammana IB, Buckley NA: **Medical management of paraquat ingestion.** *Br J Clin Pharmacol* 2011, **72:** 745-757.

578. Houze P, Baud FJ, Mouy R, Bismuth C, Bourdon R, Scherrmann JM: **Toxicokinetics of paraquat in humans.** *Hum Exp Toxicol* 1990, **9:** 5-12.

579. Kang MS, Gil HW, Yang JO, Lee EY, Hong SY: **Comparison between kidney and hemoperfusion for paraquat elimination.** *J Korean Med Sci* 2009, **24 Suppl:** S156-S160.

580. Lheureux P, Leduc D, Vanbinst R, Askenasi R: **Survival in a case of massive paraquat ingestion.** *Chest* 1995, **107:** 285-289.

581. Kuhs H, Rudolf GA: **Cardiovascular effects of paroxetine.** *Psychopharmacology (Berl )* 1990, **102:** 379-382.

582. Sindrup SH, Gram LF, Brosen K, Eshoj O, Mogensen EF: **The selective serotonin reuptake inhibitor paroxetine is effective in the treatment of diabetic neuropathy symptoms.** *Pain* 1990, **42:** 135-144.

583. Sindrup SH, Grodum E, Gram LF, Beck-Nielsen H: **Concentration-response relationship in paroxetine treatment of diabetic neuropathy symptoms: a patient-blinded dose-escalation study.** *Ther Drug Monit* 1991, **13:** 408-414.

584. Gosciniak H-T: **Suizid mit Perazin.** *Psychopharmakotherapie* 1997, **4:** 105.

585. Amoah AG, Gould BJ, Parke DV, Lockhart JD: **Further studies on the pharmacokinetics of perhexiline maleate in humans.** *Xenobiotica* 1986, **16:** 63-68.

586. Jones TE, Morris RG, Horowitz JD: **Concentration-time profile for perhexiline and hydroxyperhexiline in patients at steady state.** *Br J Clin Pharmacol* 2004, **57:** 263-269.

587. Linnet K, Wiborg O: **Steady-state serum concentrations of the neuroleptic perphenazine in relation to CYP2D6 genetic polymorphism.** *Clin Pharmacol Ther* 1996, **60:** 41-47.

588. Armstrong PJ, Bersten A: **Normeperidine toxicity.** *Anesth Analg* 1986, **65:** 536-538.

589. Baumann TJ, Smythe MA, Marikis B, Bivins BA: **Meperidine serum concentrations and analgesic response in postsurgical patients.** *DICP* 1991, **25:** 724-727.

590. Hagmeyer KO, Mauro LS, Mauro VF: **Meperidine-related seizures associated with patient-controlled analgesia pumps.** *Ann Pharmacother* 1993, **27:** 29-32.

591. Holmberg L, Odar-Cederlof I, Boreus LO, Heyner L, Ehrnebo M: **Comparative disposition of pethidine and norpethidine in old and young patients.** *Eur J Clin Pharmacol* 1982, **22:** 175-179.

592. Kaiko RF, Foley KM, Grabinski PY, Heidrich G, Rogers AG, Inturrisi CE *et al*.: **Central nervous system excitatory effects of meperidine in cancer patients.** *Ann Neurol* 1983, **13:** 180-185.

593. Anderson GD, Pak C, Doane KW, Griffy KG, Temkin NR, Wilensky AJ *et al*.: **Revised Winter-Tozer equation for normalized phenytoin concentrations in trauma and elderly patients with hypoalbuminemia.** *Ann Pharmacother* 1997, **31:** 279-284.

594. Brandolese R, Scordo MG, Spina E, Gusella M, Padrini R: **Severe phenytoin intoxication in a subject homozygous for CYP2C9*3.** *Clin Pharmacol Ther* 2001, **70:** 391-394.

595. Frey OR, von Brenndorff AI, Probst W: **Comparison of phenytoin serum concentrations in premature neonates following intravenous and oral administration.** *Ann Pharmacother* 1998, **32:** 300-303.

596. Hayes G, Kootsikas ME: **Reassessing the lower end of the phenytoin therapeutic range: a review of the literature.** *Ann Pharmacother* 1993, **27:** 1389-1392.

597. Howard CE, Roberts RS, Ely DS, Moye RA: **Use of multiple-dose activated charcoal in phenytoin toxicity.** *Ann Pharmacother* 1994, **28:** 201-203.

598. Mlynarek ME, Peterson EL, Zarowitz BJ: **Predicting unbound phenytoin concentrations in the critically ill neurosurgical patient.** *Ann Pharmacother* 1996, **30:** 219-223.

599. Murphy JM, Motiwala R, Devinsky O: **Phenytoin intoxication.** *South Med J* 1991, **84:** 1199-1204.

600. Privitera MD: **Clinical rules for phenytoin dosing.** *Ann Pharmacother* 1993, **27:** 1169-1173.

601. Asthana S, Greig NH, Hegedus L, Holloway HH, Raffaele KC, Schapiro MB *et al*.: **Clinical pharmacokinetics of physostigmine in patients with Alzheimer's disease.** *Clin Pharmacol Ther* 1995, **58:** 299-309.

602. Kietzmann D, Hamm C, Bouillon T, Kettler D, Gundert-Remy U: **Concentration-effect relationship of piritramid (Dipidolor(R)) in a postoperative pain model [abstract].** *Naunyn-Schmiedeberg's Arch Pharmacol* 1994, **349:** R139.

603. Heinz W, Trebesch I, Ulrich A, Klöser C, Kl: **Ist ein therapeutisches Drug-Monitoring bei Azolen sinnvoll? [abstract].** *Med Klin* 2008, **103:** 63.

604. Hohmann C, Kang EM, Jancel T: **Rifampin and posaconazole coadministration leads to decreased serum posaconazole concentrations.** *Clin Infect Dis* 2010, **50:** 939-940.

605. Walsh TJ, Raad I, Patterson TF, Chandrasekar P, Donowitz GR, Graybill R *et al*.: **Treatment of invasive aspergillosis with posaconazole in patients who are refractory to or intolerant of conventional therapy: an externally controlled trial.** *Clin Infect Dis* 2007, **44:** 2-12.

606. Shoji S, Suzuki M, Tomono Y, Bockbrader HN, Matsui S: **Population pharmacokinetics of pregabalin in healthy subjects and patients with post-herpetic neuralgia or diabetic peripheral neuropathy.** *Br J Clin Pharmacol* 2011, **72:** 63-76.

607. Wood DM, Berry DJ, Glover G, Eastwood J, Dargan PI: **Significant pregabalin toxicity managed with supportive care alone.** *J Med Toxicol* 2010, **6:** 435-437.

608. Goggin M, Crowley K, O'Malley K, Barry P, Kelly G, Blake J: **Serum concentrations of prilocaine following retrobulbar block.** *Br J Anaesth* 1990, **64:** 107-109.

609. Moffett BS, Cannon BC, Friedman RA, Kertesz NJ: **Therapeutic levels of intravenous procainamide in neonates: a retrospective assessment.** *Pharmacotherapy* 2006, **26:** 1687-1693.

610. Edstein MD, Veenendaal JR, Scott HV, Rieckmann KH: **Steady-state kinetics of proguanil and its active metabolite, cycloguanil, in man.** *Chemotherapy* 1988, **34:** 385-392.

611. Gandia P, Saivin S, Le-Traon AP, Guell A, Houin G: **Influence of simulated weightlessness on the intramuscular and oral pharmacokinetics of promethazine in 12 human volunteers.** *J Clin Pharmacol* 2006, **46:** 1008-1016.

612. Strenkoski-Nix LC, Ermer J, DeCleene S, Cevallos W, Mayer PR: **Pharmacokinetics of promethazine hydrochloride after administration of rectal suppositories and oral syrup to healthy subjects.** *Am J Health Syst Pharm* 2000, **57:** 1499-1505.

613. Janousek J, Paul T, Reimer A, Kallfelz HC: **Usefulness of propafenone for supraventricular arrhythmias in infants and children.** *Am J Cardiol* 1993, **72:** 294-300.

614. Hartvig P, Roos BE, Ahs U, Ryde M: **Pharmacokinetics of propiomazine following intravenous, intramuscular and oral administration with special reference to the elimination phase.** *Curr Ther Res* 1981, **29:** 351-362.

615. Arndt GA, Reiss WG, Bathke KA, Springman SR, Kenny G: **The estimated plasma concentration (EPC) at which patients are induced and awake from general anesthesia with propofol.** *Clin Pharmacol Ther* 1993, **53:** 224.

616. Iwersen-Bergmann S, Rosner P, Kuhnau HC, Junge M, Schmoldt A: **Death after excessive propofol abuse.** *Int J Legal Med* 2001, **114:** 248-251.

617. Brooks DE, Wallace KL: **Acute propylene glycol ingestion.** *J Toxicol Clin Toxicol* 2002, **40:** 513-516.

618. Centers for Disease Control and Prevention (CDC): **Infant deaths associated with cough and cold medications--two states, 2005.** *MMWR Morb Mortal Wkly Rep* 2007, **56:** 1-4.

619. Wingert WE, Mundy LA, Collins GL, Chmara ES: **Possible role of pseudoephedrine and other over-the-counter cold medications in the deaths of very young children.** *J Forensic Sci* 2007, **52:** 487-490.

620. Douglas JG, McLeod MJ: **Pharmacokinetic factors in the modern drug treatment of tuberculosis.** *Clin Pharmacokinet* 1999, **37:** 127-146.

621. White MC, De SP, Havard CW: **Plasma pyridostigmine levels in myasthenia gravis.** *Neurology* 1981, **31:** 145-150.

622. Williams NE, Calvey TN, Chan K: **Plasma concentration of pyridostigmine during the antagonism of neuromuscular block.** *Br J Anaesth* 1983, **55:** 27-31.

623. Arvanitis LA, Miller BG: **Multiple fixed doses of "Seroquel" (quetiapine) in patients with acute exacerbation of schizophrenia: a comparison with haloperidol and placebo. The Seroquel Trial 13 Study Group.** *Biol Psychiatry* 1997, **42:** 233-246.

624. DeVane CL, Nemeroff CB: **Clinical pharmacokinetics of quetiapine: an atypical antipsychotic.** *Clin Pharmacokinet* 2001, **40:** 509-522.

625. Harmon TJ, Benitez JG, Krenzelok EP, Cortes-Belen E: **Loss of consciousness from acute quetiapine overdosage.** *J Toxicol Clin Toxicol* 1998, **36:** 599-602.

626. Isbister GK, Friberg LE, Hackett LP, Duffull SB: **Pharmacokinetics of quetiapine in overdose and the effect of activated charcoal.** *Clin Pharmacol Ther* 2007, **81:** 821-827.

627. Nudelman E, Vinuela LM, Cohen CI: **Safety in overdose of quetiapine: a case report.** *J Clin Psychiatry* 1998, **59:** 433.

628. Pollak PT, Zbuk K: **Quetiapine fumarate overdose: clinical and pharmacokinetic lessons from extreme conditions.** *Clin Pharmacol Ther* 2000, **68:** 92-97.

629. von Düsterlho J, Homann J: **Lebensbedrohliche Herzrhythmusstörungen unter Chinin-Medikation.** *Dtsch Med Wochenschr* 1995, **120:** 542-543.

630. Paintaud G, Alvan G, Berninger E, Gustafsson LL, Idrizbegovic E, Karlsson KK *et al*.: **The concentration-effect relationship of quinine-induced hearing impairment.** *Clin Pharmacol Ther* 1994, **55:** 317-323.

631. Mignon M, Chau NP, Nguyen-Phuoc BK, Sauvage M, Leguy F, Bonfils S: **Ranitidine upon meal-induced gastric secretion: oral pharmacokinetics and plasma concentration effect relationships.** *Br J Clin Pharmacol* 1982, **14:** 187-193.

632. Fleishaker JC: **Clinical pharmacokinetics of reboxetine, a selective norepinephrine reuptake inhibitor for the treatment of patients with depression.** *Clin Pharmacokinet* 2000, **39:** 413-427.

633. Härtter S: **Moderne Antidepressiva: Pharmakokinetik, Interaktionspotenzial und TDM.** *Pharm Unserer Zeit* 2004, **33:** 296-303.

634. Anderson JL, Reddy CP, Myerburg RJ, Waxman HL, de Vane PJ: **Antiarrhythmic and pharmacokinetic evaluation of intravenous recainam in patients with frequent ventricular premature complexes and unsustained ventricular tachycardia.** *Am J Cardiol* 1993, **71:** 686-694.

635. Jostell KG, Lapierre YD: **Plasma concentration of remoxipride in relation to antipsychotic effect and adverse symptoms. The Canadian Remoxipride Study Group.** *Acta Psychiatr Scand Suppl* 1990, **358:48-50.:** 48-50.

636. Lappenberg-Pelzer M, Baudisch H: **Ein Todesfall nach Remoxiprideinnahme.** *Toxichem Krimtech* 1994, **61:** 10.

637. Michaelsson K, Lithell H, Vessby B, Melhus H: **Serum retinol levels and the risk of fracture.** *N Engl J Med* 2003, **348:** 287-294.

638. Kopferschmitt J, Flesch F, Lugnier A, Sauder P, Jaeger A, Mantz JM: **Acute voluntary intoxication by ricin.** *Hum Toxicol* 1983, **2:** 239-242.

639. Plomp TA, Battista HJ, Unterdorfer H, van Ditmarsch WC, Maes RA: **A case of fatal poisoning by rifampicin.** *Arch Toxicol* 1981, **48:** 245-252.

640. Peruche B, Schulz M: **Risperidon, ein neues atypisches Neuroleptikum.** *Pharm Ztg* 1996, **141:** 2920-2924.

641. Seto K, Dumontet J, Ensom MH: **Risperidone in schizophrenia: is there a role for therapeutic drug monitoring?** *Ther Drug Monit* 2011, **33:** 275-283.

642. Hsu A, Granneman GR, Bertz RJ: **Ritonavir. Clinical pharmacokinetics and interactions with other anti-HIV agents.** *Clin Pharmacokinet* 1998, **35:** 275-291.

643. Darreh-Shori T, Jelic V: **Safety and tolerability of transdermal and oral rivastigmine in Alzheimer's disease and Parkinson's disease dementia.** *Expert Opin Drug Saf* 2010, **9:** 167-176.

644. Dhillon S: **Rivastigmine transdermal patch: a review of its use in the management of dementia of the Alzheimer's type.** *Drugs* 2011, **71:** 1209-1231.

645. Kaye CM, Nicholls B: **Clinical pharmacokinetics of ropinirole.** *Clin Pharmacokinet* 2000, **39:** 243-254.

646. Emanuelsson B-M, Norsten-Höög C, Sandberg R, Sjövall J: **Ropivacaine and its 2H3-labelled analogue - bioanalysis and disposition in healthy volunteers.** *Eur J Pharm Sci* 1997, **5:** 171-177.

647. Markham A, Faulds D: **Ropivacaine. A review of its pharmacology and therapeutic use in regional anaesthesia.** *Drugs* 1996, **52:** 429-449.

648. Scott DB, Lee A, Fagan D, Bowler GM, Bloomfield P, Lundh R: **Acute toxicity of ropivacaine compared with that of bupivacaine.** *Anesth Analg* 1989, **69:** 563-569.

649. Lewis LD, Essex E, Volans GN, Cochrane GM: **A study of self poisoning with oral salbutamol--laboratory and clinical features.** *Hum Exp Toxicol* 1993, **12:** 397-401.

650. Moser M, Buchberger W: **Selenium status of elderly Austrians suffering from circulation disorders.** *Wien Klin Wochenschr* 1993, **105:** 497-499.

651. Müller D, Desel H: **Problematik, Klinik und Beispiele der Spurenelementvergiftung - Selen.** *Toxichem Krimtech* 2012, **79:** 5-16.

652. Aldosary BM, Sutter ME, Schwartz M, Morgan BW: **Case series of selenium toxicity from a nutritional supplement.** *Clin Toxicol (Phila)* 2012, **50:** 57-64.

653. Milner DA, Hall M, Davis GG, Brissie RM, Robinson CA: **Fatal multiple drug intoxication following acute sertraline use.** *J Anal Toxicol* 1998, **22:** 545-548.

654. Kahan BD, Napoli KL: **Role of therapeutic drug monitoring of rapamycin.** *Transplant Proc* 1998, **30:** 2189-2191.

655. MacDonald A, Scarola J, Burke JT, Zimmerman JJ: **Clinical pharmacokinetics and therapeutic drug monitoring of sirolimus.** *Clin Ther* 2000, **22 Suppl B:** B101-B121.

656. Mahalati K, Kahan BD: **Clinical pharmacokinetics of sirolimus.** *Clin Pharmacokinet* 2001, **40:** 573-585.

657. Zimmerman JJ, Kahan BD: **Pharmacokinetics of sirolimus in stable renal transplant patients after multiple oral dose administration.** *J Clin Pharmacol* 1997, **37:** 405-415.

658. Edvardsson N, Varnauskas E: **Clinical course serum concentrations and elimination rate in a case of massive sotalol intoxication.** *Pharmacokinetics* 1989, **6:** 558.

659. Steinecke H, Stein U, Lang J, Kluge S, Hentschel H, Muth G: **Intoxikation mit Sotalol.** *Toxichem Krimtech* 1999, **66:** 100-102.

660. Brook I: **Pharmacodynamics and pharmacokinetics of spiramycin and their clinical significance.** *Clin Pharmacokinet* 1998, **34:** 303-310.

661. Edmunds M, Sheehan TM, Van't Hoff W: **Strychnine poisoning: clinical and toxicological observations on a non-fatal case.** *J Toxicol Clin Toxicol* 1986, **24:** 245-255.

662. Heiser JM, Daya MR, Magnussen AR, Norton RL, Spyker DA, Allen DW *et al*.: **Massive strychnine intoxication: serial blood levels in a fatal case.** *J Toxicol Clin Toxicol* 1992, **30:** 269-283.

663. Oberpaur B, Donoso A, Claveria C, Valverde C, Azocar M: **Strychnine poisoning: an uncommon intoxication in children.** *Pediatr Emerg Care* 1999, **15:** 264-265.

664. Palatnick W, Meatherall R, Sitar D, Tenenbein M: **Toxicokinetics of acute strychnine poisoning.** *J Toxicol Clin Toxicol* 1997, **35:** 617-620.

665. Rosano TG, Hubbard JD, Meola JM, Swift TA: **Fatal strychnine poisoning: application of gas chromatography and tandem mass spectrometry.** *J Anal Toxicol* 2000, **24:** 642-647.

666. Winek CL, Wahba WW, Esposito FM, Collom WD: **Fatal strychnine ingestion.** *J Anal Toxicol* 1986, **10:** 120-121.

667. Wood DM, Webster E, Martinez D, Dargan PI, Jones AL: **Case report: Survival after deliberate strychnine self-poisoning, with toxicokinetic data.** *Critical Care* 2002, **6:** 456-459.

668. Bailey JM, Schwieger IM, Hug CC, Jr.: **Evaluation of sufentanil anesthesia obtained by a computer-controlled infusion for cardiac surgery.** *Anesth Analg* 1993, **76:** 247-252.

669. Borenstein M, Shupak RC, Barnette RE, Cooney GF, Tzeng T-B: **Cardiovascular effects of different infusion rate of sufentanil (S) in patients undergoing coronary surgery (CS).** *Clin Pharmacol Ther* 1994, **55:** 129.

670. Haynes G, Brahen NH, Hill HF: **Plasma sufentanil concentration after intranasal administration to paediatric outpatients.** *Can J Anaesth* 1993, **40:** 286.

671. Scholz J, Bause H, Schulz M, Klotz U, Krishna DR, Pohl S *et al*.: **Pharmacokinetics and effects on intracranial pressure of sufentanil in head trauma patients.** *Br J Clin Pharmacol* 1994, **38:** 369-372.

672. Paap CM, Nahata MC: **Clinical use of trimethoprim/sulfamethoxazole during renal dysfunction.** *DICP* 1989, **23:** 646-654.

673. Davies NM, Watson MS: **Clinical pharmacokinetics of sulindac. A dynamic old drug.** *Clin Pharmacokinet* 1997, **32:** 437-459.

674. Larsen AK: **Suramin: an anticancer drug with unique biological effects.** *Cancer Chemother Pharmacol* 1993, **32:** 96-98.

675. Hooks MA: **Tacrolimus, a new immunosuppressant--a review of the literature.** *Ann Pharmacother* 1994, **28:** 501-511.

676. Jusko WJ, Thomson AW, Fung J, McMaster P, Wong SH, Zylber-Katz E *et al*.: **Consensus document: therapeutic monitoring of tacrolimus (FK-506).** *Ther Drug Monit* 1995, **17:** 606-614.

677. Mukherjee A, Le K, Lieberman R: **Relevance of preclinical PK-PD to the clinical immunomodulatory (IMMOD) effects of FK-506 in transplantation (TX) and autoimmunity (AI).** *Clin Pharmacol Ther* 1994, **55:** 149.

678. Takahara S, Kokado Y, Kameoka H, Takano Y, Jiang H, Moutabarrik A *et al*.: **Monitoring of FK 506 blood levels in kidney transplant recipients.** *Transplant Proc* 1994, **26:** 2106-2108.

679. Undre N, Moller A: **Pharmacokinetic interpretation of FK 506 levels in blood and in plasma during a European randomised study in primary liver transplant patients. The FK 506 European Study Group.** *Transpl Int* 1994, **7 Suppl 1:S15-21.:** S15-S21.

680. Venkataramanan R, Lever J, Jain A, Burckart G, Flowers J, Warty V *et al*.: **FK506: Correlation between trough concentrations and AUC's in transplant patients [abstract].** *Pharm Res* 1993, **10:** S-390.

681. Venkataramanan R, Swaminathan A, Prasad T, Jain A, Zuckerman S, Warty V *et al*.: **Clinical pharmacokinetics of tacrolimus.** *Clin Pharmacokinet* 1995, **29:** 404-430.

682. Venkataramanan R, Shaw LM, Sarkozi L, Mullins R, Pirsch J, MacFarlane G *et al*.: **Clinical utility of monitoring tacrolimus blood concentrations in liver transplant patients.** *J Clin Pharmacol* 2001, **41:** 542-551.

683. Wallemacq PE, Verbeeck RK: **Comparative clinical pharmacokinetics of tacrolimus in paediatric and adult patients.** *Clin Pharmacokinet* 2001, **40:** 283-295.

684. Winkler M, Christians U: **A risk-benefit assessment of tacrolimus in transplantation.** *Drug Saf* 1995, **12:** 348-357.

685. Terhaag B, Grünert A, Richter K, Bahlmann G, Gloris A: **Zur Effektivität der Hämoperfusion bei einer Talinolol-Intoxikation - ein Fallbericht.** *Z Klin Med* 1987, **42:** 1463-1464.

686. Terhaag B, Möbus U, Oertel R, Richter K, Feller K: **Zur Pharmakokinetik von Talinolol (Cordanum(R)) nach wiederholter 7tägiger oraler Gabe am gesunden Probanden.** *Medicamentum* 1992, **2:** 40-44.

687. Janknegt R: **Teicoplanin in perspective. A critical comparison with vancomycin.** *Pharm Weekbl Sci* 1991, **13:** 153-160.

688. Wilson AP: **Clinical pharmacokinetics of teicoplanin.** *Clin Pharmacokinet* 2000, **39:** 167-183.

689. Forrest AR, Marsh I, Bradshaw C, Braich SK: **Fatal temazepam overdoses.** *Lancet* 1986, **2:** 226.

690. Heintz R, Stebler T, Lunell NO, Mueller S, Guentert TW: **Excretion of tenoxicam and 5'-hydroxy-tenoxicam into human milk.** *J Pharm Med* 1993, **3:** 57-64.

691. Nilsen OG: **Clinical pharmacokinetics of tenoxicam.** *Clin Pharmacokinet* 1994, **26:** 16-43.

692. Woosley RL, Chen Y, Freiman JP, Gillis RA: **Mechanism of the cardiotoxic actions of terfenadine.** *JAMA* 1993, **269:** 1532-1536.

693. Brodersen HP, Korsten S, Larbig D: **Eliminationsverfahren bei potentiell letaler Thalliumintoxikation.** *Dtsch Med Wochenschr* 1995, **120:** 1301.

694. Klemm M, Meißner D: **Problematik, Klinik und Beispiele der Spurenelementvergiftung - Thallium.** *Toxichem Krimtech* 2012, **79:** 17-22.

695. Dager WE, Albertson TE: **Impact of therapeutic drug monitoring of intravenous theophylline regimens on serum theophylline concentrations in the medical intensive care unit.** *Ann Pharmacother* 1992, **26:** 1287-1291.

696. Dettloff RW, Touchette MA, Zarowitz BJ: **Vasopressor-resistant hypotension following a massive ingestion of theophylline.** *Ann Pharmacother* 1993, **27:** 781-784.

697. Elias-Jones AC, Larcher VF, Shaw PN: **An investigation into the relationship between liver impairment and theophylline pharmacokinetics in children.** *Pharm Pharmacol Lett* 1992, **2:** 115-118.

698. Hallas J, Davidsen O, Grodum E, Damsbo N, Gram LF: **Drug-related illness as a cause of admission to a department of respiratory medicine.** *Respiration* 1992, **59:** 30-34.

699. Holford N, Black P, Couch R, Kennedy J, Briant R: **Theophylline target concentration in severe airways obstruction - 10 or 20 mg/L? A randomised concentration-controlled trial.** *Clin Pharmacokinet* 1993, **25:** 495-505.

700. Kirk JK, Dupuis RE, Miles MV, Gaddy GD, Miranda-Massari JR, Williams DM: **Salivary theophylline monitoring: reassessment and clinical considerations.** *Ther Drug Monit* 1994, **16:** 58-66.

701. Phillips BA, Chrystyn H: **The role of bayesian analysis to interpret serum theophylline concentrations in the community sector.** *Pharm J* 1991, **247:** R18.

702. Sanathanan LP, Peck CC: **The randomized concentration-controlled trial: an evaluation of its sample size efficiency.** *Control Clin Trials* 1991, **12:** 780-794.

703. Shannon M: **Predictors of major toxicity after theophylline overdose.** *Ann Intern Med* 1993, **119:** 1161-1167.

704. Sullivan P, Bekir S, Jaffar Z, Page C, Jeffery P, Costello J: **Anti-inflammatory effects of low-dose oral theophylline in atopic asthma.** *Lancet* 1994, **343:** 1006-1008.

705. Wessel T, Unger W, Wilhelms E: **Theophyllin: Drug monitoring beim internistischen Patienten sinnvoll? Versuch einer Bewertung für Arzt und Apotheker.** *Krankenhauspharmazie* 1996, **17:** 335-339.

706. Ahearn DJ, Grim CE: **Treatment of malignant hypertension with sodium nitroprusside.** *Arch Intern Med* 1974, **133:** 187-191.

707. Koniaris LG, Zimmers TA, Lubarsky DA, Sheldon JP: **Inadequate anaesthesia in lethal injection for execution.** *Lancet* 2005, **365:** 1412-1414.

708. Adkins JC, Noble S: **Tiagabine. A review of its pharmacodynamic and pharmacokinetic properties and therapeutic potential in the management of epilepsy.** *Drugs* 1998, **55:** 437-460.

709. Leach JP, Stolarek I, Brodie MJ: **Deliberate overdose with the novel anticonvulsant tiagabine.** *Seizure* 1995, **4:** 155-157.

710. Leach JP, Brodie MJ: **Tiagabine.** *Lancet* 1998, **351:** 203-207.

711. Luer MS, Rhoney DH: **Tiagabine: a novel antiepileptic drug.** *Ann Pharmacother* 1998, **32:** 1173-1180.

712. Perucca E, Bialer M: **The clinical pharmacokinetics of the newer antiepileptic drugs. Focus on topiramate, zonisamide and tiagabine.** *Clin Pharmacokinet* 1996, **31:** 29-46.

713. Davies NM: **Clinical pharmacokinetics of tiaprofenic acid and its enantiomers.** *Clin Pharmacokinet* 1996, **31:** 331-347.

714. Pottier J, Cousty-Berlin D, Busigny M: **Human pharmacokinetics of tiaprofenic acid.** *Rheumatology (Oxford)* 1982, **7:** 70-77.

715. Brennscheidt U, Brunnmuller U, Proppe D, Thomann P, Seiler KU: **Pharmacokinetics of tilidine and naloxone in patients with severe hepatic impairment.** *Arzneimittelforschung* 2007, **57:** 106-111.

716. Hajda JP, Jahnchen E, Oie S, Trenk D: **Sequential first-pass metabolism of nortilidine: the active metabolite of the synthetic opioid drug tilidine.** *J Clin Pharmacol* 2002, **42:** 1257-1261.

717. Schwietert HR, Peeters PA, Dingemanse J, Thiercelin JF, Necciari J, de BH *et al*.: **Multiple dose pharmacokinetics of tiludronate in healthy volunteers.** *Eur J Clin Pharmacol* 1996, **51:** 175-181.

718. Gillet P, Gavriloff C, Hercelin B, Salles MF, Nicolas A, Netter P: **Pharmacokinetics of tiopronin after repeated oral administration in rheumatoid arthritis.** *Fundam Clin Pharmacol* 1995, **9:** 205-206.

719. Keam SJ, Keating GM: **Tiotropium bromide. A review of its use as maintenance therapy in patients with COPD.** *Treat Respir Med* 2004, **3:** 247-268.

720. Barnfield C, Kemmenoe AV: **A sudden death due to tocainide overdose.** *Hum Toxicol* 1986, **5:** 337-340.

721. Latini R, Maggioni AP, Cavalli A: **Therapeutic drug monitoring of antiarrhythmic drugs. Rationale and current status.** *Clin Pharmacokinet* 1990, **18:** 91-103.

722. Barthel W, Hüller G, Böhm C, Haustein K-O: **Tolbutamid: Zwei Präparate im Vergleich.** *Pharm Ztg* 2007, **141:** 4686.

723. Furman WL, Baker SD, Pratt CB, Rivera GK, Evans WE, Stewart CF: **Escalating systemic exposure of continuous infusion topotecan in children with recurrent acute leukemia.** *J Clin Oncol* 1996, **14:** 1504-1511.

724. Herben VM, ten Bokkel Huinink WW, Beijnen JH: **Clinical pharmacokinetics of topotecan.** *Clin Pharmacokinet* 1996, **31:** 85-102.

725. Besson JM, Vickers MD: **Tramadol analgesia. Synergy in research and therapy.** *Drugs* 1994, **47 Suppl 1:** 1-2.

726. Lehmann KA, Kratzenberg U, Schroeder-Bark B, Horrichs-Haermeyer G: **Postoperative patient-controlled analgesia with tramadol: analgesic efficacy and minimum effective concentrations.** *Clin J Pain* 1990, **6:** 212-220.

727. Iwersen S, Schmoldt A: **One fatal and one nonfatal intoxication with tranylcypromine. Absence of amphetamines as metabolites.** *J Anal Toxicol* 1996, **20:** 301-304.

728. Meyer FP: **Trapidil (Rocornal).** *Internist Prax* 1993, **33:** 611-614.

729. Ohkubo T, Osanai T, Sugawara K, Ishida M, Otani K, Mihara K *et al*.: **High-performance liquid chromatographic determination of trazodone and 1-m-chlorophenylpiperazine with ultraviolet and electrochemical detector.** *J Pharm Pharmacol* 1995, **47:** 340-344.

730. Hukkinen SK, Varhe A, Olkkola KT, Neuvonen PJ: **Plasma concentrations of triazolam are increased by concomitant ingestion of grapefruit juice.** *Clin Pharmacol Ther* 1995, **58:** 127-131.

731. Freeman CD, Quintiliani R, Nightingale CH: **Vancomycin therapeutic drug monitoring: is it necessary?** *Ann Pharmacother* 1993, **27:** 594-598.

732. Leader WG, Chandler MH, Castiglia M: **Pharmacokinetic optimisation of vancomycin therapy.** *Clin Pharmacokinet* 1995, **28:** 327-342.

733. MacGowan A, Lovering A, White L, Reeves D: **Why monitor peak vancomycin concentrations?** *Lancet* 1995, **345:** 645-647.

734. Fernandez de Gatta MD, Calvo MV, Hernandez JM, Caballero D, San Miguel JF, Dominguez-Gil A: **Cost-effectiveness analysis of serum vancomycin concentration monitoring in patients with hematologic malignancies.** *Clin Pharmacol Ther* 1996, **60:** 332-340.

735. Pecar A, Lindner W, Mönch V, Münch G, Roos R: **Pharmacokinetics of vancomycin in preterm and term neonates.** *Krankenhauspharmazie* 1992, **13:** 591-593.

736. Welty TE, Copa AK: **Impact of vancomycin therapeutic drug monitoring on patient care.** *Ann Pharmacother* 1994, **28:** 1335-1339.

737. Klamerus KJ, Maloney K, Rudolph RL, Sisenwine SF, Jusko WJ, Chiang ST: **Introduction of a composite parameter to the pharmacokinetics of venlafaxine and its active O-desmethyl metabolite.** *J Clin Pharmacol* 1992, **32:** 716-724.

738. Lüscher TF, Noll G, Sturmer T, Huser B, Wenk M: **Calcium gluconate in severe verapamil intoxication.** *N Engl J Med* 1994, **330:** 718-720.

739. Schwab M, Oetzel C, Jägle C, Mörike K, Gleiter CH, Eichelbaum M: **Using generic names is important to prevent iatrogenic drug overdose. A case report [abstract].** *Naunyn-Schmiedeberg's Arch Pharmacol (Suppl )* 2001, **363:** R132.

740. Malabanan A, Veronikis IE, Holick MF: **Redefining vitamin D insufficiency.** *Lancet* 1998, **351:** 805-806.

741. Heaney RP: **Assessing vitamin D status.** *Curr Opin Clin Nutr Metab Care* 2011, **14:** 440-444.

742. Mata-Granados JM, Luque de Castro MD, Quesada Gomez JM: **Inappropriate serum levels of retinol, alpha-tocopherol, 25 hydroxyvitamin D3 and 24,25 dihydroxyvitamin D3 levels in healthy Spanish adults: simultaneous assessment by HPLC.** *Clin Biochem* 2008, **41:** 676-680.

743. Pazaitou-Panayiotou K, Papapetrou PD, Chrisoulidou A, Konstantinidou S, Doumala E, Georgiou E *et al*.: **Height, Whole Body Surface Area, Gender, Working Outdoors, and Sunbathing in Previous Summer are Important Determinants of Serum 25-hydroxyvitamin D Levels.** *Exp Clin Endocrinol Diabetes* 2012, **120:** 14-22.

744. Potoski BA, Brown J: **The safety of voriconazole.** *Clin Infect Dis* 2002, **35:** 1273-1275.

745. Tan K, Brayshaw N, Tomaszewski K, Troke P, Wood N: **Investigation of the potential relationships between plasma voriconazole concentrations and visual adverse events or liver function test abnormalities.** *J Clin Pharmacol* 2006, **46:** 235-243.

746. White RH, Zhou H, Romano P, Mungall D: **Changes in plasma warfarin levels and variations in steady-state prothrombin times.** *Clin Pharmacol Ther* 1995, **58:** 588-593.

747. Xu Z-X, Naadimuthu A, Lockwood G, Berger B, Maier G, Dukivic D: **Pharmacokinetic/pharmacodynamic studies of zanoterone [abstract].** *Pharm Res* 1994, **11:** S-334.

748. Fletcher CV, Balfour HH, Jr.: **Variability in zidovudine serum concentrations.** *Pharmacotherapy* 1996, **16:** 1154-1158.

749. Fletcher CV, Acosta EP, Henry K, Page LM, Gross CR, Kawle SP *et al*.: **Concentration-controlled zidovudine therapy.** *Clin Pharmacol Ther* 1998, **64:** 331-338.

750. Hoetelmans RM, Burger DM, Meenhorst PL, Beijnen JH: **Pharmacokinetic individualisation of zidovudine therapy. Current state of pharmacokinetic-pharmacodynamic relationships.** *Clin Pharmacokinet* 1996, **30:** 314-327.

751. Chen T, Berenson J, Vescio R, Swift R, Gilchick A, Goodin S *et al*.: **Pharmacokinetics and pharmacodynamics of zoledronic acid in cancer patients with bone metastases.** *J Clin Pharmacol* 2002, **42:** 1228-1236.

752. Skerjanec A, Berenson J, Hsu C, Major P, Miller WH, Jr., Ravera C *et al*.: **The pharmacokinetics and pharmacodynamics of zoledronic acid in cancer patients with varying degrees of renal function.** *J Clin Pharmacol* 2003, **43:** 154-162.

753. De Luca A., Lamura L, Gallo M, Daniele G, D'Alessio A, Giordano P *et al*.: **Pharmacokinetic evaluation of zoledronic acid.** *Expert Opin Drug Metab Toxicol* 2011, **7:** 911-918.

754. Debailleul G, Khalil FA, Lheureux P: **HPLC quantification of zolpidem and prothipendyl in a voluntary intoxication.** *J Anal Toxicol* 1991, **15:** 35-37.

755. Garnier R, Guerault E, Muzard D, Azoyan P, Chaumet-Riffaud AE, Efthymiou ML: **Acute zolpidem poisoning--analysis of 344 cases.** *J Toxicol Clin Toxicol* 1994, **32:** 391-404.

756. Winek CL, Wahba WW, Janssen JK, Rozin L, Rafizadeh V: **Acute overdose of zolpidem.** *Forensic Sci Int* 1996, **78:** 165-168.

757. Kochak GM, Page JG, Buchanan RA, Peters R, Padgett CS: **Steady-state pharmacokinetics of zonisamide, an antiepileptic agent for treatment of refractory complex partial seizures.** *J Clin Pharmacol* 1998, **38:** 166-171.

758. Mimaki T: **Clinical pharmacology and therapeutic drug monitoring of zonisamide.** *Ther Drug Monit* 1998, **20:** 593-597.

759. Schulz M, Schmoldt A: **Therapeutic and toxic blood concentrations of more than 800 drugs and other xenobiotics.** *Pharmazie* 2003, **58:** 447-474.

760. Bircher J: **Verbesserte Dosierung von Medikamenten durch Messung ihrer Plasmakonzentrationen.** *Ther Umsch* 1977, **34:** 830-834.

761. Bircher J, Sommer W: *Klinisch-pharmakologische Datenbsammlung*, 2 edn. Stuttgart: WVG; 1999.

762. Brosen K: **Drug-metabolizing enzymes and therapeutic drug monitoring in psychiatry.** *Ther Drug Monit* 1996, **18:** 393-396.

763. Caccia S, Garattini S: **Formation of active metabolites of psychotropic drugs. An updated review of their significance.** *Clin Pharmacokinet* 1990, **18:** 434-459.

764. Deom A: **L'intoxication d'origine inconnue, l'apport du laboratoire pour le clinicien.** *Ther Umsch* 1986, **43:** 259-268.

765. Deom A: **Valeurs usuelles des taus sanguins, urinaires et autres lors de traitements ou lors d'intoxications chez l'homme.** *Ther Umsch* 1986, **43:** 261-268.

766. Dinovo EC, Gottschalk LA, McGuire FL, Birch H, Heiser JF: **Analysis of results of toxicological examinations performed by coroners' or medical examiners' laboratories in 2000 drug-involved deaths in nine major U. S. cities.** *Clin Chem* 1976, **22:** 847-850.

767. Drayer DE: **Pharmacologically active drug metabolites: therapeutic and toxic activities, plasma and urine data in man, accumulation in renal failure.** *Clin Pharmacokinet* 1976, **1:** 426-443.

768. Drayer DE: **Problems in therapeutic drug monitoring: the dilemma of enantiomeric drugs in man.** *Ther Drug Monit* 1988, **10:** 1-7.

769. Gottschalk LA, Cravey RH: *Toxicological and pathological studies on psychoactive drug-involved deaths*. Davis: Biomedical Publishing; 1980.

770. Jack DB: *Handbook of clinical pharmacokinetic data*. Basingstoke: Macmillan; 1992.

771. Moffat AC, Jackson JV, Moss MS, Widdop B: *Clarke's isolation and identification of drugs in pharmaceuticals, body fluids, and post-mortem material*, 2 edn. London: Pharmaceutical Press; 1986.

772. Ochs HR, Gugler R: **Drug Monitoring in der Intensivmedizin.** *Internist (Berl )* 1984, **25:** 336-340.

773. Oellerich M, Sybrecht GW, Klein H: **Therapieüberwachung durch Bestimmung von Plasmakonzentrationen im Serum.** *Internist (Berl )* 1982, **23:** 174-181.

774. Paterson SC: **Drug levels found in cases of fatal self-poisoning.** *Forensic Sci Int* 1985, **27:** 129-133.

775. Pentz B, Strubelt O, Gehlhoff C: **Therapeutische, toxische und letale Arzneimittelkonzentrationen im menschlichen Plasma.** *Dt Ärztebl* 1979, **43:** 2815-2820.

776. Roots I: **Wann ist die Bestimmung von Arzneimittelkonzentrationen im Plasma nützlich und notwendig?** *Internist (Berl )* 1986, **27:** 40-52.

777. Rosenkranz B, Frölich JC: **Plasmakonzentrationen von Arzneimitteln: Wann messen, wie interpretieren?** *Dt Ärztebl* 1985, **82:** B-1769-B-1778.

778. Spector R, Park GD, Johnson GF, Vesell ES: **Therapeutic drug monitoring.** *Clin Pharmacol Ther* 1988, **43:** 345-353.

779. Stead AH, Moffat AC: **A collection of therapeutic, toxic and fatal blood drug concentrations in man.** *Hum Toxicol* 1983, **2:** 437-464.
